# Scalable Manufacturing and Precise Patterning of Perovskites for Light-Emitting Diodes

**DOI:** 10.1007/s40820-025-02012-8

**Published:** 2026-01-05

**Authors:** Shuaiqi Liu, Hao Jiang, Jizhuang Wang, Li Liu, Zhiwen Zhou, Mojun Chen

**Affiliations:** 1 Smart Manufacturing Thrust, The Hong Kong University of Science and Technology (Guangzhou), Systems Hub, Guangzhou, 511458 People’s Republic of China; 2 Department of Electronic Engineering, The Chinese University of Hong Kong, Shatin, 999077 Hong Kong, SAR People’s Republic of China; 3 School of Materials Science and Engineering, Harbin Institute of Technology (Shenzhen), Shenzhen, 518055 People’s Republic of China; 4 National Key Laboratory of Precision Hot Processing of Metals, Harbin Institute of Technology, Harbin, 150001 People’s Republic of China; 5 College of Chemistry and Materials Science, Guangdong Provincial Key Laboratory of Supramolecular Coordination Chemistry, Jinan University, Guangzhou, 510632 People’s Republic of China; 6 Department of Mechanical and Aerospace Engineering, The Hong Kong University of Science and Technology, Clear Water Bay, Kowloon, 999077 Hong Kong, People’s Republic of China

**Keywords:** Perovskite materials, Scalable manufacturing, Precise patterning, Light-emitting diodes

## Abstract

Owing to the exceptional optoelectronic properties, metal halide perovskites have emerged as leading semiconductor materials for next-generation display technologies, providing perovskite light-emitting diodes (PeLEDs) great potential for high-quality color displays with a wide color gamut and pure color emission. Although laboratory-scale PeLEDs have achieved near-theoretical efficiencies, challenges such as achieving uniform large-area films, improving material stability, and enhancing patterning precision remain barriers to commercialization. This review presents a systematic analysis of scalable manufacturing and precision patterning strategies for PeLEDs, focusing on their applications in large-area lighting and full-color displays. Fabrication methods are categorized into film deposition techniques (spin-coating, blade-coating, and thermal evaporation) and patterning strategies, including top-down (photolithography, laser/e-beam lithography, and nanoimprinting) and bottom-up (patterned crystal growth, inkjet printing, and electrohydrodynamic jet printing) approaches. In this review, we discuss the advantages and limitations of each strategy, highlight current challenges, and outlook possible pathways towards scalable, high-performance PeLEDs for advanced optoelectronic applications.

## Highlights

- This review provides a comprehensive exploration of advanced film and patterning fabrication techniques for high-performance perovskite light-emitting diodes (PeLEDs).
- This review examines both top-down and bottom-up techniques, such as photolithography and inkjet printing to achieve precise patterning of PeLEDs for full-color displays.
- This review discusses critical challenges, including device stability, scalable manufacturing, and microscale pixel patterning, as well as promising strategies to overcome these obstacles for the commercialization of PeLEDs.

## Graphical abstract

**Figure:**
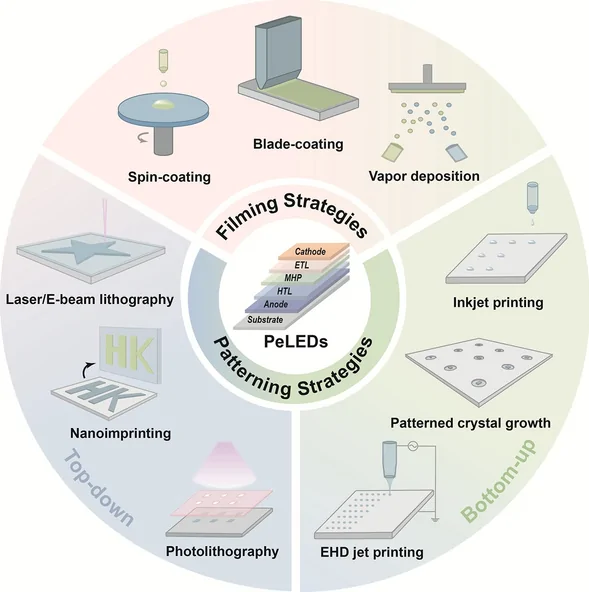

## Introduction

Metal halide perovskites (MHPs) are a class of emerging semiconductor materials with exceptional potential for next-generation optoelectronic technologies. The typical structure of MHPs follows the ABX₃ formula, where A represents monovalent cations (such as Cs⁺, Rb⁺, methylammonium (MA⁺), and formamidinium (FA⁺), B represents divalent metal cations (such as Sn^2^⁺ and Pb^2^⁺), and X denotes halide anions (such as Cl⁻, Br⁻, and I⁻) [1–6]. MHPs exhibit unique optoelectronic properties, primarily including high absorption coefficients, broad bandgap tunability, long carrier diffusion lengths, and excellent defect tolerance [7–9], which make them highly promising for a variety of applications, especially in the field of solar cells [10–15]. Over just a decade, perovskite solar cells (PSCs) have achieved impressive power conversion efficiencies (PCE) of 27.3% (perovskite single junction) [16], 33.89% (perovskite/silicon) [17], 29.1% (all perovskite tandem) [18], marking a significant breakthrough and driving forward the research and commercialization of MHP materials in the photovoltaic industry.

Perovskite light-emitting diodes (PeLEDs), a promising next-generation display technology, have also attracted widespread attention as the photovoltaic application. Compared to traditional liquid crystal displays (LCDs) and organic light-emitting diodes (OLEDs), PeLEDs offer numerous advantages, including narrower emission linewidths (15–20 nm), wide color gamuts, and high contrast ratios [5, 19–21]. These features enable PeLEDs to reach up to 140% of the national television system committee (NTSC) color standard, making them highly promising for applications in displays [3, 21, 22]. Moreover, the diverse chemical compositions of MHPs allow their optical properties to be highly tunable, enabling continuous spectral tuning from the blue-violet to near-infrared regions, further enhancing their appeal in optoelectronic applications [2, 3, 23, 24]. In 2014, Tan et al. [25] successfully demonstrated the first room-temperature green and near-infrared emitting PeLEDs, opening a new chapter in LED research. Since then, the performance of PeLEDs has rapidly improved, with external quantum efficiencies (EQE) for green, red, and near-infrared emitting devices exceeding 25%, and the EQE of blue PeLEDs surpassing 20% [26–31]. These breakthroughs have positioned PeLEDs on par with the most advanced organic and quantum dot LEDs. Beyond display technologies, PeLEDs hold significant promise for flexible and wearable electronics due to the inherent mechanical flexibility of perovskite materials [32, 33]. Flexible PeLEDs could enable innovations such as foldable displays [34–36] and biocompatible imaging devices [21, 32, 37].

Despite the remarkable progress in PeLED technology, several challenges remain before PeLEDs can be widely adopted in consumer products. First, translating high-performance perovskite films from small laboratory devices to large-area substrates introduces significant non-uniformities in thickness, crystallinity, and defect density, which degrade device efficiency and shorten operational lifetime. Ensuring reproducible film quality across such scales is therefore critical for achieving consistent, industry-scale performance of PeLEDs [38–41]. Second, the development of high-resolution patterning techniques for full-color displays adds further complexity. Achieving precise pixel control down to tens of micrometers while maintaining the optoelectronic properties of the perovskite material is critical for the success integration of PeLEDs into practical applications [42, 43]. Moreover, integrating pure red, green, and blue (RGB) emitting units into subpixels with precise control of color and pattern remains a critical challenge for commercialization, since full-color displays with high resolution and wide color gamut are essential for consumer electronics such as smartphones and virtual reality (VR) devices [21, 34]. This requires microscale alignment of subpixels and suppression of cross-talk between adjacent emission layers, both of which are hindered by material instability and fabrication complexity [43]. Together, these dual requirements of wide-area uniformity and precise pixel-level patterning define the engineering benchmark for scalable, high-resolution PeLED manufacturing. Moreover, other key issues including long-term operational stability, light outcoupling efficiency, and resistence to thermal and environmental degradation, also need to be addressed to extend the applicability of PeLEDs [44–46].

In this review, we aim to provide a comprehensive overview of representative fabrication strategies of perovskite materials, with a particular focus on large-area film fabrication and high-resolution patterning strategies in light-emitting diodes. According to the target morphology and processing requirements of MHP, we classify the fabrication techniques for perovskite into two main sections: film fabrication and patterning techniques. The first section covers three typical film fabrication methods, including spin-coating, blade-coating, and thermal evaporation. In the second section, we focus on patterning techniques, which are further categorized into two main approaches based on their processing mechanisms. Top-down methods include photolithography, laser/e-beam lithography, and nanoimprinting, whereas bottom-up methods involve patterned crystal growth, inkjet printing, and electrohydrodynamic (EHD) jet printing. The principles, advantages, and drawbacks of each fabrication method are analyzed, with specific emphasis on their applications in full-color displays. Finally, we discuss future directions for large-area fabrication and patterning technologies, highlighting their roles in advancing high-performance PeLEDs toward industry-level commercialization.

## Film Fabrication Strategies

**Fig. 1:**
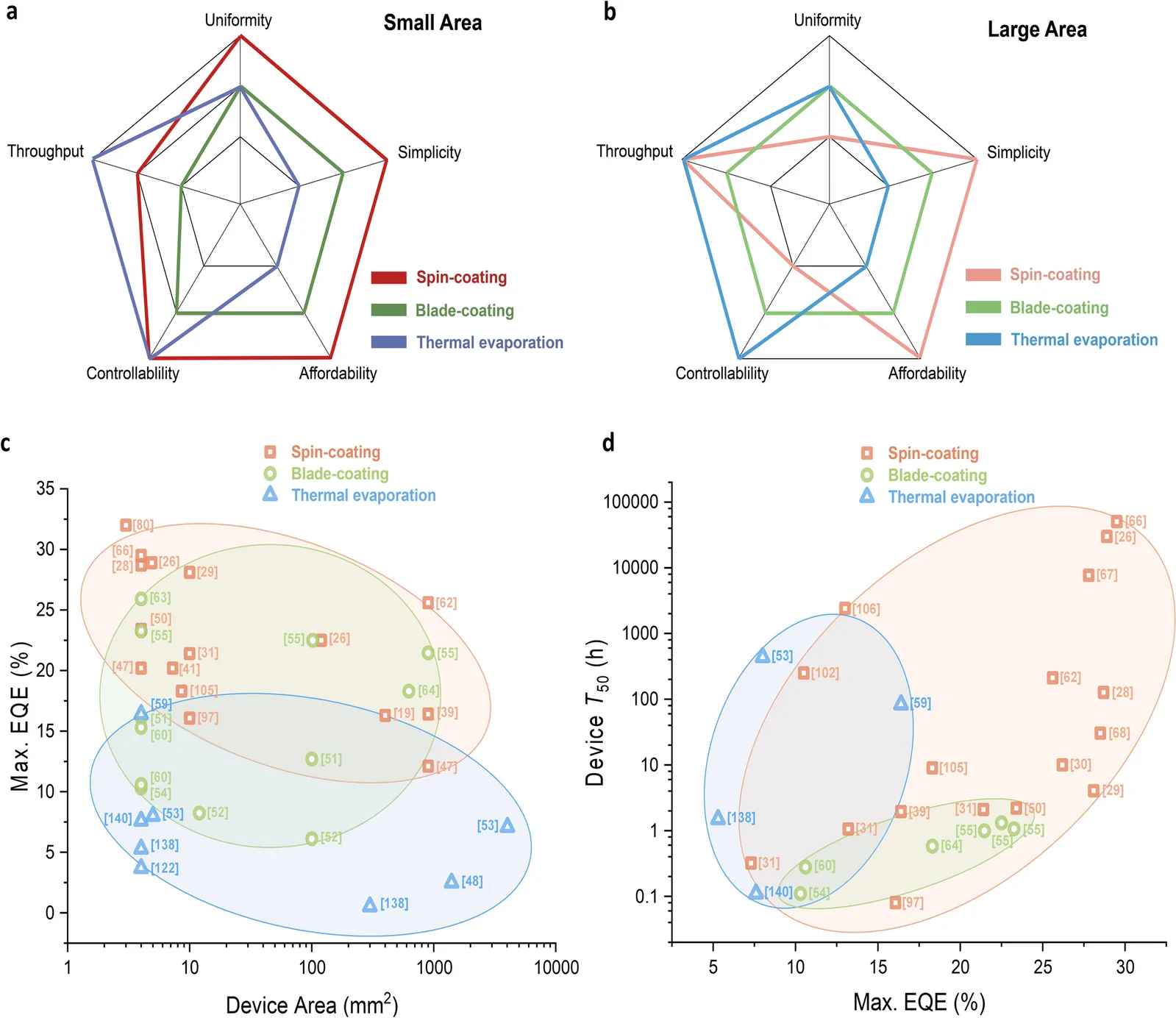
Perovskite filming strategies. **a** Radar plots comparing different perovskite film deposition techniques for small-area devices (< 10 mm^2^). **b** Radar plots comparing different perovskite film deposition techniques for large-area devices (≥ 100 mm^2^). “Affordability” refers to the cost-effectiveness of fabrication strategy. “Controllability” refers to the ability to achieve the desired thickness, area, and quality of perovskite films. **c** Statistical scatter plot showing the maximum EQE versus device area for PeLEDs studies conducted over the past 5 years. **d** Statistical scatter plot showing the device half-operation lifetime (@100 cd m^− 2^) versus maximum EQE for PeLEDs studies conducted over the past 5 years

Deposition of high-quality and scalable MHP emission layers is critical for PeLED manufacturing. Figure 1a, b compares three main deposition strategies: spin-coating, blade-coating, and thermal evaporation, for small- and large-area PeLEDs. In this work, we define small-area PeLEDs as devices with active area < 10 mm^2^ and large-area PeLEDs as those with active area ≥ 100 mm^2^. This empirical partition follows the distribution of published device areas (Fig. 1c), where most reports cluster below ~ 10 mm^2^ (laboratory studies) or above ~ 100 mm^2^ (scale-up demonstrations), while the intermediate range (~ 10–100 mm^2^) remains comparatively underexplored. The scarcity of studies in the 10–100 mm^2^ window is mainly due to (i) the incompatibility of many lab-scale deposition/patterning methods with intermediate-area substrates without substantial re-engineering, (ii) scale-dependent drying and flow dynamics that change nucleation/uniformity when moving away from small pixels, and (iii) practical applications focus either to small pixel-scale demonstrations or to large-area proof-of-concept panels [44]. Spin-coating is highly effective for small-area films, offering excellent quality, controllability, and low cost. This is attributed to rapid and uniform solvent evaporation driven by centrifugal forces, which ensures controlled nucleation and homogeneous crystallization. However, when extended to large areas, spin-coating faces challenges in scalability and uniformity, resulting in less consistent film quality and thickness, due to uneven solvent evaporation and centrifugal force distribution at larger radii. Blade-coating, by contrast, is cost-effective for large-area applications, providing good scalability and controllability. Its effectiveness at large scale arises from continuous blade movement that facilitates controlled film spreading. But variations in solvent evaporation rates and local concentration gradients during coating lead to less uniform nucleation and crystallization, thus sacrificing crystallinity and uniformity over small area compared to spin-coating. Thermal evaporation stands out for its superior scalability and film uniformity, making it ideal for industrial-scale production. The high uniformity originates from the precisely controlled vapor-phase deposition process under vacuum conditions, ensuring consistent molecular flux and film thickness distribution. Nevertheless, its low material utilization and the requirement for vacuum environments increase complexity and costs.

We further compare three methods in EQE, device area, and half-operation lifetime (@ 100 cd m^−2^) aspects in Fig. 1c, d and summarized key metrics of large-area PeLEDs in Table 1. Spin-coating, the most commonly employed technique for perovskite thin-film preparation, achieves remarkably high EQE and long lifetime due to its advanced defect passivation strategies [66–68]. However, its scalability to larger areas is limited, reducing its practicality for industrial-scale manufacturing. Blade-coating offers a more balanced approach, enabling cost-effective deposition over medium-to-large areas. While its EQE and operational lifetime are typically lower than those of spin-coated small-area devices, which mainly reasoned by reduced uniformity and crystallinity, its superior scalability makes it more suitable for larger-scale applications [69, 70]. In contrast, thermal evaporation excels in large-area perovskite film fabrication, offering outstanding scalability and precise thickness control. However, devices produced by this method typically exhibit relatively low EQEs (mostly below 10%). This limited performance primarily arises from the inconsistent deposition temperatures of bromide precursors, which lead to a high density of defects. Additionally, the inherently weak surface interactions during vapor-phase deposition often result in sparse nucleation and rough film morphologies, further diminishing optoelectronic performance [40]. Overall, spin-coating is best suited for high-performance, small-area devices, while thermal evaporation is advantageous for large-scale production, and blade-coating provides a practical middle ground between performance and scalability.

**Table 1:** The summary of main characteristics of large-area (≥ 100 mm^2^) PeLEDs over the past five years

Year	Strategy	Emission layer (wavelength)	Maximum device area (mm^2^)	Maximum EQE (%, @maximum area)	*T*_50_ (h, @maximum area, 100 cd m^− 2^)	Refs
2020	Spin-coating	FAPbI_3_ (799 nm)	900	12.1	10 (@10 mA cm^− 2^)	[47]
2020	Spin-coating	FAPbBr_3_ (531 nm)	400	16.3	2.9 (*T*_80_)	[19]
2020	Thermal evaporation	CsPbBr_3_ (520 nm)	1400	~ 2	7 (@500 cd m^− 2^)	[48]
2021	Spin-coating	PEA_2_(FA_0.7_Cs_0.3_)_2_Pb_3_Br_10_ (520 nm)	900	16.4	1.95	[39]
2021	Spin-coating	CsPb(Br/Cl)_3_ (490 nm)	400	6.1	0.16	[49]
2021	Spin-coating	FA_0.9_GA_0.1_PbBr_3_ PeNCs (535 nm)	900	-	-	[50]
2021	Blade-coating	MAPbI_3_ (725 nm)	2800	-	10.1 (@3 mA cm^− 2^)	[51]
2021	Blade-coating	(PEA)_2_Cs_n-1_Pb_n_Br_3n+1_ (530 nm)	1225	-	-	[52]
2021	Thermal evaporation	CsPbBr_3_ (508 nm)	4020	7.1	7.3	[53]
2022	Spin-coating	(FA_0.7_MA_0.1_GA_0.2_)_0.87_ Cs_0.13_PbBr_3_ (540 nm)	120	22.5	-	[26]
2022	Blade-coating	CsPb(Br_0.84_Cl_0.16_)_3_ (489 nm)	2800	-	-	[54]
2022	Blade-coating	FA_x_GA_1-x_PbBr_3_ (530 nm)	900	21.46	1	[55]
2022	Thermal evaporation	MAPbBr_3_ (517 nm)	7850	-	-	[56]
2023	Spin-coating	CsPbBr_3-x_Cl_x_/CsPbBr_3_/CsPbBr_3-x_I_x_ (478/512/630 nm)	1050	-	-	[57]
2023	Spin-coating	(PEA)_2_Cs_n-1_Pb_n_Br_3n+1_ (516 nm)	1225	12.1	-	[58]
2023	Thermal evaporation	CsPbBr_3_ (523 nm)	7995	-	-	[59]
2024	Spin-coating	CsPbI_3_ (645 nm)	900	28.7	126.8	[28]
2024	Blade-coating	CsPbI_3_ (650 nm)	2800	-	-	[60]
2025	Spin-coating	CsPbBr_2.5_Cl_0.5_ (508 nm)	400	2.81	2.25	[61]
2025	Spin-coating	CsPbI_3_ (630 nm)	900	25.6	211	[62]
2025	Blade-coating	(PEA,Cs)PbBr_3_ (495 nm)	3500	-	-	[63]
2025	Blade-coating	FAPbI_3_ (780 nm)	625	18.3	0.58	[64]
2025	Thermal evaporation	FA_x_Cs_1−x_PbI_y_Br_3−y_ (675 nm)	2500	-	-	[65]

### Spin-coating

Spin-coating is a widely used technique where a small volume of solution is deposited onto a substrate fixed on a rotating stage. It spreads the solution radially onto the surface of a rotating substrate through centrifugal force (proportional to the angular velocity and plate radius), and its uniformity depends on the dynamic balance between the viscosity gradient caused by solvent evaporation and the distribution of centrifugal force. Although the centrifugal force at the edge is greater, rapid solvent evaporation can suppress the radial thickness difference and form a uniform gel-like layer. By adjusting the rotational speed (controlling centrifugal force), time (affecting evaporation rate), and antisolvent addition (promoting uniform nucleation), the morphology, crystallinity, and thickness uniformity of the film can be optimized [71–75]. Beyond solvent/antisolvent control, spin-coated PeLED films benefit markedly from rational additive engineering: (i) Lewis-base/ligand additives (e.g., H_2_O microadditives, phosphine-oxide, or amide donors) coordinate Pb^2+^ and stabilize solvate intermediates, slowing crystallization to yield denser, more uniform films over centimeter scales [76, 77]; (ii) ionic/bulky A-site additives (e.g., PEACl, MACl, FABr; pseudohalides) regulate halide homogenization and promote vertically oriented quasi-2D/3D stacks, suppressing phase segregation and non-radiative traps [78, 79]. Synergistic dual-additive strategies can simultaneously accelerate radiative recombination and passivate defects—particularly valuable for red/near-infrared (NIR) compositions where exciton–phonon coupling is stronger and spectral broadening is more pronounced [80, 81]. At the interfaces, self-assembled monolayers (SAMs) and ultrathin passivation layers provide complementary levers for scalable uniformity: (i)SAMs (e.g., PACz-type or co-adsorbed ionic SAMs) tune surface energy and dipoles to improve wetting, increase nucleation density, and reduce interfacial trap-assisted quenching; (ii)SAM-assisted blue PeLEDs, for instance, show order-of-magnitude EQE gains by suppressing non-radiative loss at the perovskite/hole transport layer (HTL) interface [82]. Through multiple optimization strategies, spin-coating is particularly effective for producing MHP films with uniform structures and high crystallinity at small scales, making it a standard approach for fabricating PeLEDs at the laboratory [5, 30, 50, 83–85]. However, it is limited by edge flow effects and radial thickness gradients, making it difficult to directly extend to large-scale applications [69].

The electroluminescent (EL) linewidth of PeLEDs remains distinctly narrow in the visible yet broadens toward the near-infrared, with trends that follow both intrinsic (exciton–phonon) and extrinsic (compositional/structural) broadening mechanisms [86, 87]. State-of-the-art green PeLEDs based on bromide 3D/NC films routinely show full width at half maximum (FWHM) ~ 18–20 nm, while blue/deep-blue emitters (typically Br/Cl mixed or low-dimensional phases) are usually ~ 20–30 nm, which suffer color drift and line broadening from field-accelerated halide segregation and phase redistribution unless halide homogenization and defect passivation are enforced [44, 67, 88–91]. Red devices (iodide-rich) commonly exhibit ~ 28–35 nm, though exceptional systems (e.g., highly passivated α-CsPbI_3_) can approach ~ 21–28 nm [92]. By contrast, NIR PeLEDs generally display broader lines ~ 40–50 nm (representative reports ~ 41–46 nm) owing to stronger exciton–phonon coupling at lower bandgaps and greater inhomogeneity from grain/strain distributions [93, 94].

**Fig. 2:**
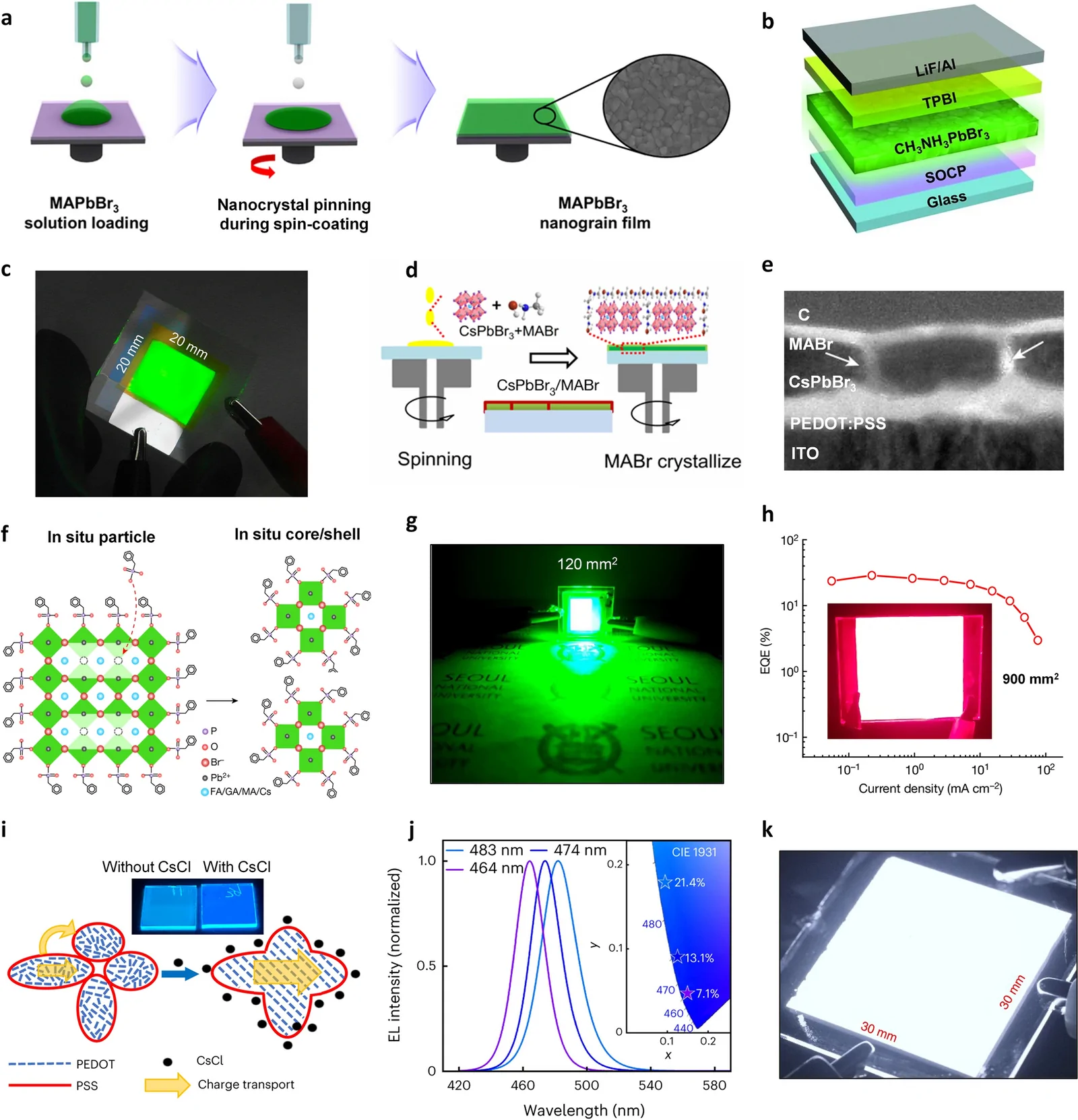
Spin-coating strategy for PeLEDs. **a** Schematics of NCP process during spin-coating to fabricate MAPbBr_3_ nanograin films, **b** the corresponding device structure, and **c** a photograph of large-area (20 mm by 20 mm pixel) PeLEDs [95]. **d** Schematic formation process of the CsPbBr_3_/MABr quasi-core/shell structure. **e** Cross-sectional TEM image of the quasi-core/shell CsPbBr_3_/MABr structure. White arrows indicate the MABr shell (the grain boundary) [96]. **f** Schematic illustration of the transformation process of in situ particle into in situ core/shell structures by BPA treatment. **g** Photograph of an operating large-area device (pixel size: 120 mm^2^) [26]. **h** EQE versus current density curve of PeLEDs based on MM-MOPA perovskite; the inset is an optical photograph of a 900 mm^2^ PeLED under 4 V voltage [28]. **i** Proposed schematic of the structural evolution of PEDOT:PSS after CsCl incorporation. The inset shows the emission images under ultraviolet excitation (365 nm) [97]. **j** EL spectra of PPNCl-containing PeLEDs with different Cl^–^/Br^–^ ratios (driven by 4.5 V). The inset shows the corresponding CIE coordinates [31]. **k** NIR image of the 900 mm^2^ large-area PeLED on glass [47]

In 2014, Tan et al. [25] demonstrated the first room-temperature PeLED device fabricated using the spin-coating method. The perovskite layer in their devices was designed to be extremely thin (~ 15 nm) to spatially confine electrons and holes, thereby enhancing radiative recombination efficiency. Despite achieving a modest EQE of only 0.1%, the low cost and versatility of this approach sparked significant research interest for further exploration. A subsequent breakthrough was achieved by Cho et al., who improved both the EQE and current efficiency through stoichiometric adjustments and a nanocrystal pinning (NCP) process (Fig. 2a) [95]. To further reduce the grain size, they incorporated an organic molecule of 2,2′,2″-(1,3,5-benzinetriyl)-tris (1-phenyl-1-H-benzimidazole) (TPBI) as an additive in the NCP solvent. By using a self-organized conducting polymer (SOCP) as the anode, they successfully fabricated a flexible large-area PeLED device with a 20 mm by 20 mm pixel for the first time (Fig. 2b, c). This method facilitated the in situ formation of MAPbBr_3_ nanograins, which confined excitons within the nanograins, thereby suppressing exciton quenching. As a result, their green PeLEDs achieved a maximum EQE value of 8.53%.

To enhance LED performance, two strategies subsequently emerged: direct spin-coating of colloidal perovskite nanocrystals (PeNCs) [98, 99] and engineering perovskite precursor solutions for bulk films [83, 100, 101]. The first approach utilized the high photoluminescence quantum yield (PLQY) of PeNCs, along with their tunable optical properties achieved through compositional adjustments and control of crystal size. In contrast, the second strategy focuses on depositing bulk perovskite films by optimizing the composition of precursor solutions, allowing for precise tailoring of the material properties to enhance film quality and device performance. Beyond these two approaches, a significant advancement was reported by Lin et al. in 2018, who achieved an impressive EQE of 20.3% using a CsPbBr_3_/MABr quasi-core/shell structure [96]. The MABr shell could passivate the non-radiative defects that often present in CsPbBr_3_ and enhance the charge injection balance. This quasi-core/shell structure, formed by blending pre-synthesized CsPbBr_3_ perovskite powder with an MABr additive (Fig. 2d), demonstrated great benefits for crystallinity improvement and defect passivation of the emitting layer, making it a promising approach for enhancing the performance and longevity of PeLEDs. The cross-section TEM image (Fig. 2e) evidenced well-aligned layer-by-layer structure and the boundaries formed by MABr. Another remarkable milestone was reached by Kim et al., who combined a three-dimensional (3D) perovskite film formed via an additive-based nanocrystal pinning (A-NCP) technique with in situ formation of a core/shell structure using benzylphosphonic acid (BPA) solution combined with tetrahydrofuran (THF) post-treatment [26]. Specifically, the in situ particle structure was created by forming covalent bonds between the surficial undercoordinated Pb^2+^ of the 3D perovskite matrixes with BPA additives. The strong acidity and small size of the BPA additive enable the penetration and intercalation of BPA molecules into larger perovskite crystals, leading to the cleavage of the large crystal domains and formation of a nanosized core/shell structure. This was achieved by exposing the particle structure to a BPA/THF solution, which reduced the large crystals into nanosized cores and shells surrounded by BPA (Fig. 2f). As a result, the grain size distribution of the perovskite structure was significantly minimized, decreasing from 205 ± 97 nm (for the 3D structure) to 10 ± 2 nm (for the in situ core/shell). Ultimately, this innovative approach yielded a maximum EQE of 28.9% and a peak current efficiency of 151 cd A^− 1^, while maintaining over 20% efficiency under ultra-high brightness conditions exceeding 400,000 cd m^− 2^. Furthermore, leveraging the core/shell structure, they successfully fabricated a 120 mm^2^ PeLED that exhibited high brightness, excellent uniformity, and a maximum EQE of 22.5% (Fig. 2g).

Compared with green PeLEDs, although several works achieved great improvement in EQE of red PeLEDs, it is still challenging to realize pure-red (620–650 nm) LEDs with good stability [102–106]. To enhance the performance of PeLEDs in the red spectrum, Kong et al. [28] developed a method to stabilize the I–Pb–I octahedron by introducing 3-methoxyphenethylammonium (MOPA) into reduced-dimensional perovskite structures. Unlike conventional ammonium ligands, which provide weak hydrogen bonding to anchor the lattice, MOPA features a double-ended anchored ligand that includes both ammonium and methoxy groups. This structure enhances hydrogen bonding interactions and effectively coordinates lead ions, thereby suppressing I^−^ migration under an electric field and reducing non-radiative recombination. These enhancements led to the ultralong operation lifetime (7610 min @100 cd m^− 2^) for the fabricated PeLEDs with a maximum EQE of 28.7%, achieving bright and uniform pure red emission at 638 nm over an active area of 30 mm × 30 mm (Fig. 2h).

Despite significant advancements in the performance of green- and red-emitting PeLEDs using bromine- and iodine-based perovskites, achieving efficient and stable PeLEDs with blue emission (between 450 and 490 nm) using chlorine-based materials remains challenging due to poor charge injection, high amounts of non-radiative recombination, and random phase distribution [49, 107–109]. Addressing the issue of inefficient charge injection, Chu et al. incorporated CsCl into the hole injection layer (HIL), poly(3,4-ethylenedioxythiophene) polystyrene sulfonate (PEDOT:PSS), resulting in the improving of charge-carrier transport and better band alignment with the perovskite emissive layers (Fig. 2i). Based on such strategy, fabricated sky-blue PeLEDs centered at 486 nm achieved an EQE of 16.07%. Moreover, Yuan et al. recently developed efficient blue-emitting LEDs using a reduced-dimensional mixed-halide perovskite with the additive bis(triphenylphosphine)iminium chloride (PPNCl), which improves phase tuning toward highly emissive quasi-3D structures and reduces defects by forming bonds with organic and lead components during crystallization. The resulting films achieve over 88% PLQY, enabling blue PeLEDs with a maximum EQE of 21.4%, 13.2%, and 7.3 peaking at 483, 474, and 464 nm, respectively (Fig. 2j). This work offers a promising approach for fabricating next-generation high-performance blue PeLEDs.

However, large-area PeLEDs fabricated using the spin-coating method typically exhibit degraded EQEs, brightness, current efficiency, and uniformity compared to their small-area counterparts [19]. This performance shortfall is primarily due to the decreased uniformity in film flatness and the uncontrollable crystallization process that leads to inhomogeneous diffusion and crystallization between the edge and center of the film. Although the increased defect density and reduced homogeneity pose challenges for scaling spin-coating techniques to larger PeLED devices, it remains a valuable approach for developing perovskite precursors and additive decorations for large-area films.

Without the need for further purification or ligand-exchange steps, spin-coating can yield high-quality quasi-2D films, which have been successful in small-area PeLEDs thanks to their self-assembled multiple-quantum-well structure [39]. However, the simple one-step spin-coating deposition strategy, even when enhanced by “antisolvent-assisted” techniques—shown to be highly effective for fabricating emitting quasi-2D films—faces challenges in producing large-area homogeneous films. Investigating the underlying issues, Sun et al. discovered that the antisolvent does not diffuse uniformly to the edge regions, where bulk 3D and layered 2D phases dominate. To address this, they introduced L-norvaline (NVAL) into the precursor solution to replace the PEA^+^ ligand, creating a COO^−^-coordinated intermediate phase with lower formation enthalpy. This approach enabled the production of a large-area (2500 mm^2^) quasi-2D film with high homogeneity, bright emission, and high PLQY. Consequently, the large-area green PeLED achieved a maximum EQE of 16.4% with an active device area of up to 900 mm^2^.

Rather than tackling the non-uniformity of large-area PeLED devices, Zhao et al. [47] concentrated on enhancing hole injection efficiency, a significant factor affecting performance. By incorporating 5-aminovaleric acid (5-AVA) as an additive, their fabricated PeLEDs exhibited passivated traps and improved microstructure. Additionally, they replaced the hole transport layer with poly-TPD (poly(N,N′-bis(4-butylphenyl)-N,N′-bisphenylbenzidine)), which has a shallow ionization potential. This strategy led to high-efficiency small-area PeLEDs measuring 4 mm^2^, achieving an EQE of 20.2% with a narrow standard deviation, as well as a large-area PeLED of 900 mm^2^ that demonstrated outstanding brightness, uniformity across the entire device, and an EQE of 12.1% (Fig. 2k).

### Blade-coating

**Fig. 3:**
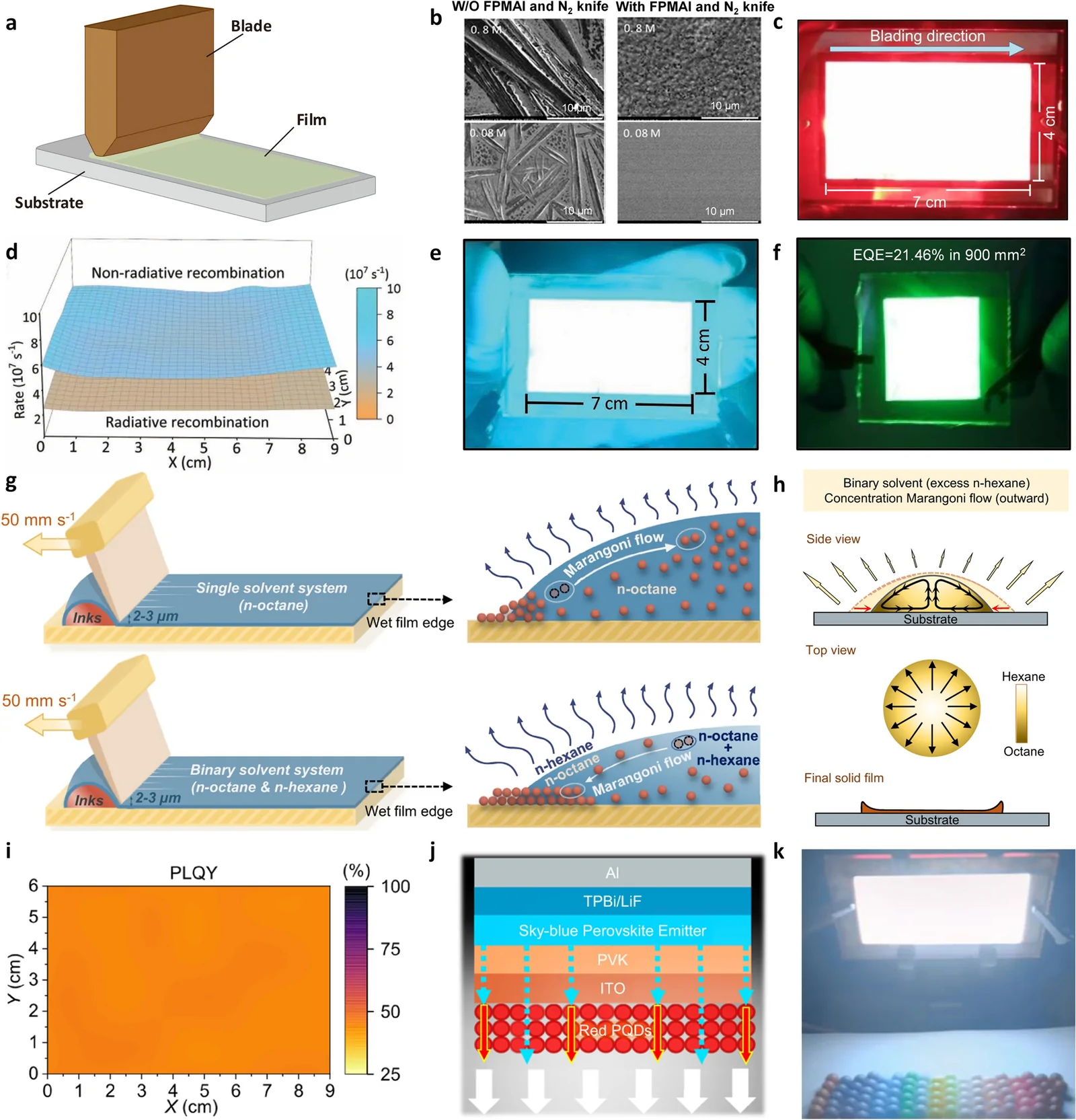
Blade-coating strategy for perovskite films and PeLEDs. **a** Schematics of the blade-coating setup. **b** SEM images of the doctor-bladed films fabricated with/without FPMAI or an N_2_ knife. **c** Photograph image of a red large-area PeLED (40 × 70 mm^2^) [51]. **d** Radiative and non-radiative recombination rate mapping of large-area films. **e** Photograph image of a sky-blue large-area PeLED (40 × 70 mm^2^) [54]. **f** Photograph of a m-bar-coated PeNC film-based large-area PeLED (900 mm^2^) [55]. **g** Schematics of the fabrication process of PeQD films by blade-coating with the single solvent and binary-solvent system. **h** Schematic illustration of solution droplet evaporation and final film patterns with excess ratios of n-hexane. **i** PLQY mapping of a large-area PeQD film (60 × 90 mm^2^). **j** Device structure of white PeLEDs. **k** Photograph image of a white large-area PeLED (40 × 70 mm^2^) [60]

Compared to spin-coating, which is restricted by limited substrate size, blade-coating provides a scalable method for producing large-area perovskite films and optoelectronic devices like PSCs and PeLEDs [69, 110–115]. The coating process involves depositing the precursor solution uniformly across the surface of substrate by controlling the movement of blade or substrate (Fig. 3a) [114]. Fundamentally, this process is governed by shear-driven fluid dynamics, where the blade-induced shear force aligns precursor molecules and suppresses uncontrolled convection, enabling homogeneous solution spreading [114]. The quality and thickness of the formed films depend not only on the setup parameters, including travel speed, placement angle, and gap distance, but also on the viscosity of the precursor solution, the wettability of the substrate, and the adhesion interface between them [111]. The balance between viscous forces and capillary flow dictates the film uniformity, while solvent evaporation kinetics during the sol–gel transition critically influence crystallization pathways and defect formation [115]. Thanks to its rapid and efficient formation of uniform thin films over large surface area, low equipment cost, high compatibility, and high material utilization rate, blade-coating is generally considered as one of the most favorable strategies for the large-area PeLEDs fabrication [52].

In 2016, Bade et al. [116] pioneered the use of the blade-coating method for fabricating PeLEDs. However, due to a lack of control over crystallization kinetics, the resulting perovskite films exhibited micrometer-scale grain sizes and excessive thickness (1–2 μm), leading to imbalanced charge carrier transport, enhanced non-radiative recombination, and poor light outcoupling efficiency. Consequently, the devices achieved an EQE of only 1.1%. Unlike PSCs, which benefit from large grains and thick films, PeLED active layers require small grain sizes and ultrathin films to realize charge carrier confinement and enable efficient charge injections [117, 118]. Blade-coated MHP films also retain more residual solvent compared to spin-coated films, leading to slow and uneven solvent evaporation during the sol–gel stage. This often results in high surface roughness and inhomogeneous crystallization. To accelerate solvent evaporation, the substrate is usually heated during the blade-coating process [112, 119]. However, elevated substrate temperatures also induce rapid solute migration and aggregation, complicating the formation of uniform and smooth films [69]. Consequently, the blade-coating method faces considerable challenges in achieving high-performance PeLEDs, primarily due to the high surface roughness and undesirable crystal morphology caused by uncontrolled crystallization during the deposition process.

To address these challenges, researchers have explored innovative strategies to improve solvent evaporation and film morphology during the blade-coating process. Among these, utilizing a gas knife, such as N_2_ or other inert gases, offer a significant advantage over the single solid blade in accelerating solvent evaporation. By employing an N_2_ knife-assisted low-temperature blade-coating approach, Chu et al. successfully prepared uniform, large-area perovskite films with great uniformity in film thickness and roughness, and improved optoelectronic properties [51]. They modified the blade-coating process by diluting precursor concentration, lowering the substrate temperature down to 50 °C, and incorporating 4-fluorophenylmethylammonium iodide (FPMAI) as an additive to overcome the inherent inhomogeneous crystallization at the sol–gel stages. The diluted precursor with excess organoammonium dramatically reduced the whole sol–gel period, promoting dense nucleation centers, and accelerating phase transformation. This approach significantly decreased the roughness of perovskite films, yielding ultra-flat large-area films with roughness as low as 1 nm (Fig. 3b). The resulting doctor-bladed red-emission PeLEDs achieved a peak EQE of 16.1%. This strategy also enabled the fabrication of an ultra-large-area PeLED (2800 mm^2^) with uniform EL emission, demonstrating its compatibility with large-scale manufacturing processes (Fig. 3c). Chu et al. further extended this blade-coating method to the fabrication of large-area PeLEDs with stable sky-blue emission. By partially replacing dimethyl sulfoxide (DMSO) with dimethylformamide (DMF) to obtain a more volatile supersaturated solution, they successfully modulated the nucleation process from gas-solution interface to whole solution phase, enabling much higher nucleation sites, and a faster crystallization rate [54]. The peak EQE of their blade-coated PeLEDs reached 10.3% with a blue emission wavelength of 489 nm, and the large PeLED device also showed a bright and uniform sky-blue emission, demonstrating quite small fluctuations of both radiative and non-radiative recombination rates over the entire film (Fig. 3d, e).

Chen et al. demonstrated the vacuum quenching-assisted blade-coating process at room temperature for the fabrication of large-area green-emission PeLEDs [52]. To avoid the solution migration and aggregation induced by nonuniform heat-transfer, they creatively put the newly coated precursor wet films into a vacuum chamber to evaporate excess solvent, followed by an annealing post-treatment. The fabricated LED devices achieved high EQEs of 8.24% and 6.12% on emitting areas of 0.12 and 1 mm^2^, respectively. Meanwhile, a large LED device with an emitting area 35 × 35 mm^2^ was also fabricated, which exhibited bright and uniform emission characteristics. As mentioned in the spin-coating section, colloidal PeNCs hold great potential for manufacturing controllable high-quality perovskite films without suffering from rapid crystallization or nonuniform phase distribution. This is because PeNCs are pre-synthesized and surrounded by organic ligands, which shield them from the adverse effects typically associated with the film formation process—such as high-temperature annealing, solvent-induced degradation, and uncontrolled crystallization—that can otherwise deteriorate the material’s structural and optoelectronic properties [120]. Besides, with efficient charge carrier confinement and fast exciton recombination, PeNCs films exhibit bright luminescence with narrow widths, making them promising candidates for the fabrication of large-area PeLEDs. Kim et al. exploited the full advantages of colloidal PeNCs for the large-area efficient PeLEDs with improved EL efficiency, high uniformity, and reproducibility [55]. They printed the pre-prepared FAPbBr_3_ NCs with a modified bar-coating method in which a tilted substrate by more than 50° was adopted to accelerate the evaporation of resident solvent. The surface of FAPbBr_3_ NCs was capped with organic ligands (n-decylamine and oleic acid), which could effectively suppress ion migration and charge trapping during the operation of PeLEDs. By this way, they demonstrated a highly efficient green PeLED with an EQE of 21.46% across the 900 mm^2^ device area, which is comparable to the best efficiency of small-area PeLEDs fabricated by spin-coating (Fig. 3f). Their efforts also indicate that colloidal PeNCs might be more suitable than polycrystalline films for applying printed large-area, high-efficiency PeLEDs for industrial displays and solid-state lighting.

Similar to colloidal PeNCs, premade perovskite quantum dots (PeQDs) can also decouple crystallization from the film formation process, making them a promising material for large-area PeLEDs. However, due to their smaller size (less than 10 nm) and higher surface-to-volume ratio, PeQDs are more ionic and sensitive to polar solvents, posing challenges in their dispersion and deposition [2]. To address this, Shi et al. [60] developed a blade-coating approach using a binary-solvent system (n-octane and n-hexane) to control the solvent fluidic dynamics and achieve uniform PeQD film deposition. As illustrated in Fig. 3g, the introduction of n-hexane into n-octane solvent prevents the accumulation of PeQDs by inducing an outward Marangoni flow, leading to uniform and dense film formation. Meanwhile, the proper amount of n-hexane is also of great significance; otherwise, faster evaporation strengthens the outward flow and leads to premature drying during spreading with excess n-hexane (Fig. 3h). This method enabled the fabrication of large-area film with uniform PLQY (Fig. 3i) and red-emitting PeLEDs with a peak EQE of 15.3%. To exploit the full advantages of the binary-solvent approach of making uniform perovskite films, they also fabricated a white PeLED (EQE > 10.6%) by combining a sky-blue PeLED with a red PeQD layer as the downconverter (Fig. 3j) and successfully produced a large-area (2800 mm^2^) white PeLED with excellent brightness and uniformity (Fig. 3k).

### Thermal Evaporation

**Fig. 4:**
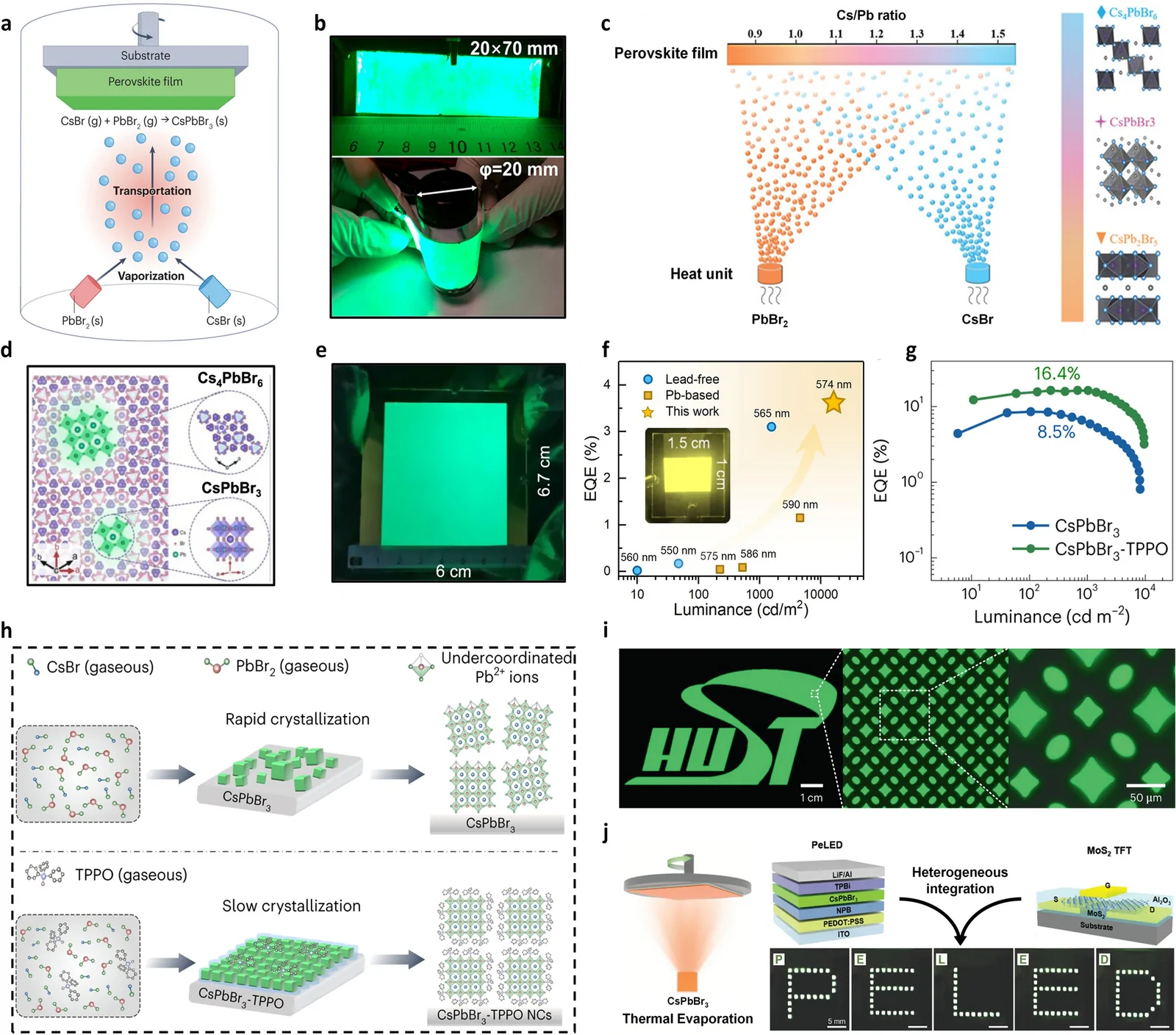
Thermal evaporation strategy for PeLEDs. **a** Schematic of a dual-source thermal evaporation of perovskite CsPbBr_3_ films [40]. **b** Photograph of a large-scale flexible PeLED (70 mm × 20 mm) [48]. **c** Schematic of dual-source thermal evaporation for perovskite film, right insets are structures of Cs_4_PbBr_6_, CsPbBr_3_, and CsPb_2_Br_5_ [121]. **d** Schematic diagram of CsPbBr_3_ embedded in the Cs_4_PbBr_6_ matrix, illustrating the separation of 3D [PbBr_6_]^4−^ of CsPbBr_3_ induced by 0D [PbBr_6_]^4−^ of Cs_4_PbBr_6_. **e** Photograph of a thermally evaporated 4020 mm^2^ PeLED [53]. **f** Statistical charts of EQE verses luminance of the yellow PeLEDs with emission in the range of 550–600 nm. The inset shows a photograph image of CsPbBr_2_I film-based 15 × 10 mm^2^ yellow PeLED [122]. **g** EQE verses luminance curves for CsPbBr_3_ and CsPbBr_3_-TPPO-based devices. **h** Schematics of crystallization process for thermally evaporated CsPbBr_3_ and CsPbBr_3_-TPPO films. **i** Display of “HUST” pattern and its arrays of emissive pixel units after magnification by microscope [59]. **j** Left shows schematic illustration of the deposition of CsPbBr_3_ powder. Right shows heterogenous integration of CsPbBr_3_ PeLED display with a MoS_2_ TFT-based backplane and photographs of the operating PeLED display representing each letter of “PELED” [123]

Thermal evaporation is generally considered as a mature technology, commonly used in electrode coating, semiconductor deposition, and large-scale OLED production [40, 124–126]. In recent years, thermal evaporation technique has attracted great interest for the fabrication of MHPs in PSCs and PeLEDs [127–130]. This strategy is also referred as vapor deposition, which involves the controlled deposition of vaporized precursor materials onto a substrate in a vacuum. As illustrated in Fig. 4a, the vapor deposition process starts with the co-evaporation of two halide precursors (typically an alkali-metal halide and a lead halide) which react on the substrate to form the perovskite [40]. In LED-oriented vacuum deposition, inorganic salts are preferred over organic ammonium halides (e.g., MABr/FABr) because the latter partially decompose under high vacuum at deposition temperatures, complicating flux control and stoichiometry, whereas CsBr/PbBr_2_ co-evaporation reproducibly yields dense CsPbBr_3_ films [131–133]. Nucleation occurs as vapor condenses, forming small clusters that grow into crystals. The nucleation density and subsequent crystal growth are strongly influenced by parameters such as deposition rate, substrate temperature, and surface energy: slower deposition rates and optimized substrate heating promote adatom surface diffusion, leading to larger crystal domains and reduced defect density. Post-deposition annealing often further improves crystallinity by facilitating atom rearrangement and grain coalescence [82].

However, the thermal evaporation process also introduces several challenges. In vacuum co-evaporation of CsPbBr₃ from CsBr and PbBr_2_, the two precursors have markedly different vapor pressures/sublimation temperatures (PbBr_2_ reaches useful vapor pressures in the ~ 340–360 °C range, whereas CsBr generally requires ~ 600–700 °C), so even small drifts in source temperature or crucible loading can bias the Cs/Pb flux ratio, leading to non-stoichiometry [134, 135]. These regions often form deep-level defects that act as non-radiative recombination centers, increasing trap-assisted recombination and reducing PLQY [40]. In addition, because adatom–substrate interactions for halide perovskites in vacuum are relatively weak, adatom diffusion lengths are large and nucleation densities can be sparse, yielding coarser grains and higher RMS roughness than the rapid, supersaturation-driven nucleation typical of antisolvent solution routes. This difference in the early growth regime explains why vacuum films often require additional measures to reach the morphology and defect levels routinely achieved by solution processing [136, 137]. To mitigate these issues, approaches include: closed-loop flux control with PbBr_2_/CsBr ratio tuning, substrate-temperature windows that promote reaction yet avoid decomposition, post-annealing to complete solid-state conversion and heal defects, hybrid thermal evaporation assists to strengthen interfacial reaction/wetting, and nucleation-seeding interlayers to raise nucleus density [65, 128]. Despite these limitations, thermal evaporation offers excellent scalability and precise thickness control due to its directional, collision-free vapor transport in vacuum, which minimizes scattering and contamination [128]. The ability to form well-defined films with tunable composition makes it a compelling choice for large-area manufacturing, provided that the crystallization and stoichiometry challenges can be effectively addressed [138].

To the best of our knowledge, Hu et al. [139] pioneered the use of the thermal evaporation method for fabricating an all-inorganic PeLED in 2017, employing a dual-source co-evaporation technique with an equimolar of CsBr and PbBr_2_. However, due to the low crystallinity of the films, their evaporation-based PeLEDs achieved an EQE of only 1.55%, significantly lower than that of solution-processed PeLEDs during the same period. The poor crystallinity of MHP films was the main culprit, hindering the performance of these evaporated PeLEDs. Countering this issue, Chen et al. [48] in 2020 discovered that applying a moderate in situ annealing temperature (60 °C) during the deposition process improved crystallinity and produced better grain orientation. By utilizing this strategy, they successfully fabricated the first thermally evaporated large-area PeLED (20 mm × 70 mm) with excellent uniformity (Fig. 4b).

On the other hand, while Hu et al. focused on optimizing the stoichiometric ratio through chemical composition engineering, the effects of composition variation on crystallization dynamics and phase evolution were not deeply explored in their study. To address this gap, Li et al. [121] employed an all-vacuum deposition process to fabricate CsPbBr_3_-based PeLEDs and systematically investigated the influence of the Cs/Pb ratio on phase purity and device performance. By using a nonrotating substrate, they achieved a gradient composition in the perovskite film, as illustrated in Fig. 4c. The Cs/Pb ratio increased from the region above the PbBr_2_ source to that above the CsBr source, leading to the formation of mixed CsPb_2_Br_5_ and CsPbBr_3_ phases, which transitioned to CsPbBr_3_ and Cs_4_PbBr_6_ phases. By replacing LiF with NiO_x_ for the hole-injection layer, all-vacuum deposition PeLEDs reached an EQE of 3.26%. Building on these findings, Li et al. [53] further demonstrated in 2021 that zero-dimensional (0D) Cs_4_PbBr_6_ could be incorporated into a CsPbBr_3_ film via simply modifying the Cs/Pb ratio. This led to the formation of a Cs_4_PbBr_6_/CsPbBr_3_ core–shell structure, where Cs_4_PbBr_6_ acted as the matrix and CsPbBr_3_ as nanoinclusions. The size of the CsPbBr_3_ nanoinclusions determines the strength of quantum confinement effect (Fig. 4d). This spatial confinement significantly enhanced carrier recombination efficiency, enabling the fabrication of an ultra-large PeLED (4020 mm^2^) with an EQE of up to 7.1% and uniform green emission across the entire device area (Fig. 4e), showcasing the potential of thermally evaporated PeLEDs for next-generation large-area displays. In 2022, Li et al. [122] extended this approach by replacing CsBr with CsI in the chamber crucible, fabricating yellow-emitting PeLEDs via a similar all-vacuum evaporation method. Owing to the great importance of controlling the grain size and thickness of perovskite layer, they regulated the growth kinetics by modifying the co-evaporation rate. Consequently, the PeLEDs based on the optimal perovskite film demonstrated the yellow EL (574 nm) with a maximum EQE of 3.7%, and the large-area PeLED (150 mm^2^) exhibited great uniformity and brightness (Fig. 4f).

In addition to in situ annealing and spatial confinement, Kim et al. and Song et al. further improved the EQE of thermally evaporated PeLEDs by introducing innovative passivation techniques. Kim et al. [140] introduced a polyethylene oxide (PEO) interlayer doped with MgCl_2_ beneath vacuum-evaporated CsPbBr_3_ films, increasing the EQE to approximately 7.6%, making it one of the highest-performing vacuum-processed PeLEDs at the time. In contrast, Song et al. [141] implemented a bilateral interfacial passivation strategy—treating both sides of the perovskite layer—which resulted in a significantly higher EQE, exceeding 15%, along with enhanced operational stability. Recently, Li et al. [122] achieved a milestone breakthrough by developing a tri-source co-evaporation strategy incorporating triphenylphosphine oxide (TPPO) as a Lewis-base additive, which increased the EQE of thermally evaporated PeLEDs to a maximum of 16.4% (Fig. 4g). As illustrated in Fig. 4h, TPPO, with the P = O band, acts as an electron donor that binds to PbBr_2_, serving as a surface ligand to constrain crystal growth. This not only impedes the crystallization of CsPbBr_3_ but also promotes the formation of small crystal grains with enhanced charge carrier confinement, effectively passivating the perovskite crystals. Based on this strategy, they fabricated active-matrix PeLED displays by integrating top-emitting PeLEDs onto a 6.67-inch thin-film transistor backplane. These displays demonstrated high-definition patterns and videos with a resolution of 1,080 × 2,400 and continuous greyscale information (Fig. 4i), showcasing the potential of thermally evaporated PeLEDs for advanced display technologies.

Further advancing the field, Ji et al. [123] explored heterogeneous integration by combining single-source evaporation-synthesized perovskite films with molybdenum disulfide (MoS_2_)-based thin-film transistors (TFTs) to create a unified optoelectronic system. The thermal evaporation process utilized a mixture of CsBr and PbBr_2_, with HBr incorporated to generate uniform large-area films through vapor deposition (Fig. 4j). Although limited to a maximum EQE of 2.21%, Ji et al. successfully fabricated an 8 × 8 active-matrix luminescent device using ALD-coated MoS_2_ TFTs, which demonstrated stable operation, precise brightness control, and negligible response delay. Five characters, “P,” “E,” “L,” “E,” and “D,” were successfully displayed by integrated device (Fig. 4j), highlighting the potential of heterogeneous integration for developing functional electronic systems with enhanced performance and scalability.

## Patterning Strategies

Achieving high-quality perovskite thin films is essential for optoelectronic performance; however, the transition from uniform films to micro- and nanopatterned architectures represents a critical frontier—where crystal engineering for emerging photoelectronics intersects with integrated device array technology. Precise patterning of perovskite materials is particularly vital for full-color PeLED displays, as it directly impacts pixel resolution, color purity, and device performance. When pixel lateral size *L* is reduced (typically < 50 μm), PeLED efficiency often declines because the perimeter-to-area ratio rises, so etched/processed sidewalls dominate non-radiative recombination. Simultaneously, to reach a given luminance, the required current density increases, aggravating Auger/Joule heating and current crowding, and field fringing at the contact edges may introduce leakage paths [43]. In fact, the size penalty is not intrinsic to perovskites and can be mitigated by process/device design. For example, active-matrix PeLED arrays show little efficiency loss relative to their large-area prototypes, and localized-contact micro/nano-PeLEDs maintain ~ 20% EQE while downscaling pixel length from 650 to 3.5 µm by preventing boundary non-radiative losses [142]. Practical levers for patterned integration include: (i) damage-minimizing pattern transfer and conformal sidewall passivation to suppress edge recombination; (ii) charge-balance engineering and current-spread control; (iii) high-PLQY, uniform emitters via additive/interface control to preserve film quality in micropatterned pixels [30, 43, 143]. Together, these measures substantially narrow the performance gap between small-area, patterned pixels and their larger-area counterparts.

**Fig. 5:**
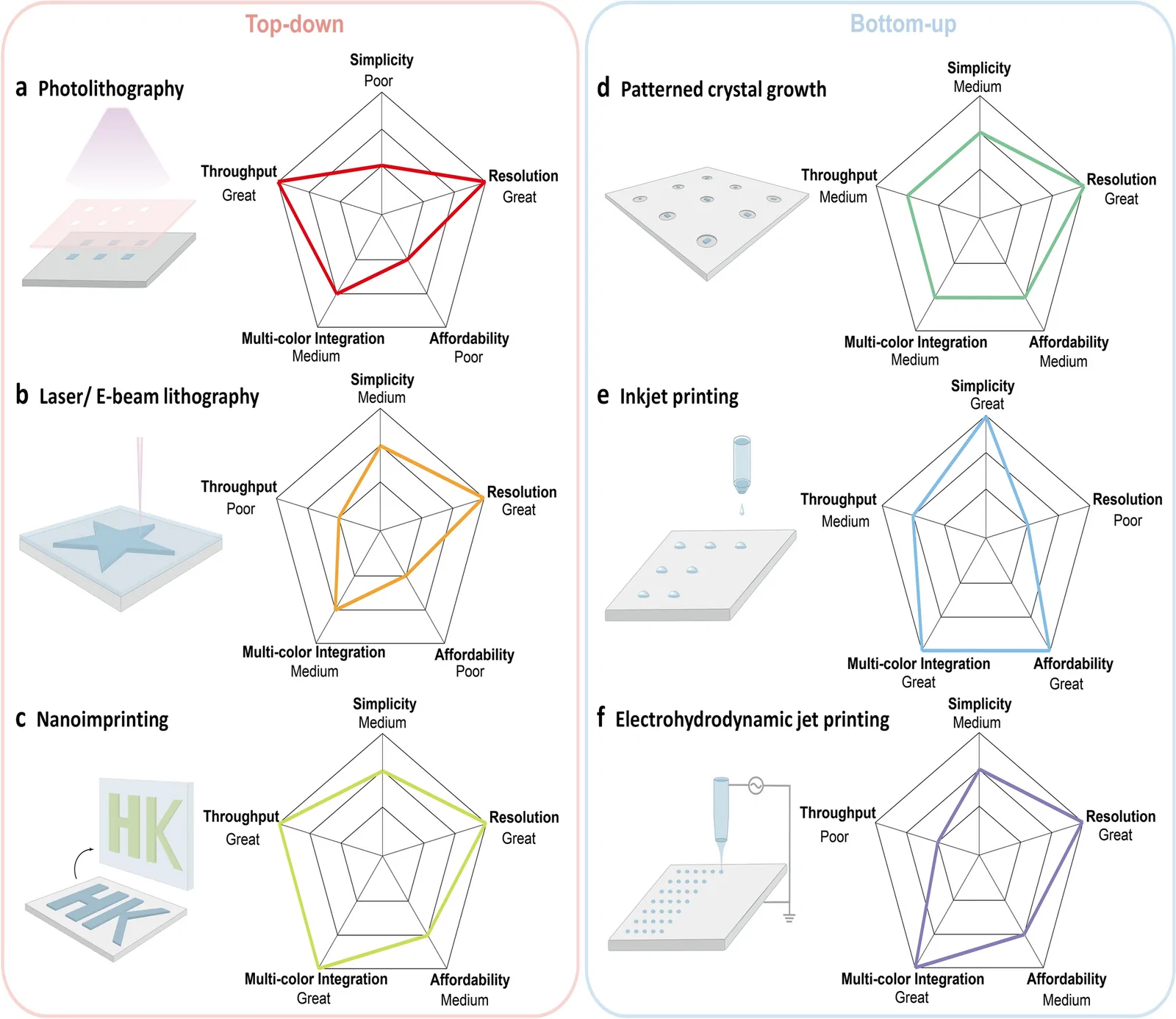
Perovskite patterning strategies. **a-c** Schematics and radar plots for top-down perovskite patterning techniques. **d-f** Schematics and radar plots for bottom-up perovskite patterning techniques. “Multi-color Integration” refers to the controllability of RGB emission units within a single pixel

Patterning techniques for perovskites can be broadly classified into top-down and bottom-up approaches, depending on their underlying processing mechanisms. Top-down methods (Fig. 5a-c)—including photolithography, laser/e-beam lithography, and nanoimprinting—typically rely on subtractive processes such as etching, ablation, or imprinting to define high-resolution features. These techniques offer exceptional spatial precision and geometric control, making them attractive for research-scale device prototyping. However, their inherent serial processing nature, especially in laser and e-beam lithography, limits throughput and scalability. Moreover, perovskite materials often exhibit sensitivity to the solvents, heat, and high-energy exposure used in these techniques, posing material compatibility challenges.

In contrast, bottom-up methods (Fig. 5d, e), such as pre-patterned crystal growth, inkjet printing, and EHD jet printing, are based on the solution processability and low formation energy of perovskites, enabling material assembly under ambient or low-temperature conditions. These techniques exhibit superior material utilization and reduced fabrication waste, making them attractive for sustainable manufacturing. However, challenges remain in achieving nanoscale resolution, and crystallization dynamics must be precisely controlled to ensure pattern uniformity and reproducibility. Recent advancements in PeLED patterning strategies have significantly improved scalability, resolution, and multi-color integration, paving the way for high-performance and full-color displays (Table 2). In the following section, we systematically examine and compare these six key patterning techniques, analyzing their working principles, technical advantages, and practical applications in PeLED manufacturing.

**Table 2:** The summary of main characteristics of patterned PeLEDs over the past five years

Year	Strategy	Emission layer (wavelength)	Minimum pattern size (μm^2^)	Maximum area (mm^2^)	Maximum EQE (%)	*T*_50_ (h, @100 cd m^− 2^)	Refs
2020	E-beam lithography	MAPbBr_3_ (521 nm)	~300	4	0.1	-	[145]
2020	Inkjet printing	FA_0.3_Cs_0.7_PbBr_3_(520 nm)	~9000	-	2.8	0.12(@90 cd m^− 2^)	[146]
2020	Photolithography	PEA-CsPbI_3_/PEA-CsPbBr_3_/PEA-CsPbBr_1.5_Cl_1.5 _(670/523/482 nm)	~300	4	1.24	-	[147]
2021	Inkjet printing	CsPbBr_3_ (515 nm)	~10000	-	3.03	-	[148]
2021	Inkjet printing	PEA-CsPbI_3_/PEA-CsPbBr_3_/PEA-CsPbBr_3_ (645/515/486 nm)	~9000	10	3.5(R), 3.4(G), 1.0(B)	0.03(@128 cd m^− 2^)	[149]
2021	Inkjet printing	MAPbBr_3 _(536 nm)	~25000	-	0.804	-	[150]
2021	Inkjet printing	CsPbBr_3 _(518 nm)	~6075	1288	9	0.13(@120 cd m^− 2^)	[151]
2022	Laser lithography	CsPbBr_3_ (512 nm)	~900	25	-	-	[152]
2022	Transfer printing	Cs_0.7_MA_0.3_Pb(I_0.8_Br_0.2_)_3_ and CsPb(Br_0.84_Cl_0.16_)_3_ (680/493 nm)	25	225	10.5	0.75	[153]
2022	Nanoimprinting	CsPbBr_x_I_3-x_ (650.9 nm)	~100	4	5.9	0.1	[154]
2022	Nanoimprinting and transfer printing	CsPbBr_0.6_Cl_0.4_/CsPbBr_3_/CsPbI_3_ (480/516/670 nm)	3	~ 9	15.3(R), 14.8(G), 2.5(B)	0.35	[34]
2022	Inkjet printing	CsPbBrI_2_/CsPbBr_3_/CsPbCl_1.56_Br_1.44_ (646/514/465 nm)	~675	625	0.83(R), 0.42(G), 0.05(B)	0.13(R), 0.04(G), 0.01(B)	[155]
2022	Inkjet printing	PEA_2_SnI_4_ (633 nm)	-	32	1	~ 3	[156]
2022	Inkjet printing	CsPbBr_3_ (517 nm)	~6075	555	8.54	1.1	[157]
2022	Inkjet printing	CsPb(I/Br)_3_ (640 nm)	~9000	-	9.6	0.19	[158]
2023	Inkjet printing	MAPbBr_3_/PEG (530 nm)	-	1600	0.5	-	[159]
2023	Crystal growth	CsPb 6-4Cl-Br/4-6Cl-Br/CsPbBr_3_/I-Br (478/491/512/630 nm)	40,000	1050	9.97(R), 26.09(G), 16.49(sky-B), 12.41(B)	0.39	[57]
2024	Nanoimprinting	PEA-CsPbBr_x_Cl_3-x_ (471.2 nm)	~75	4	6.1	-	[160]
2024	Inkjet printing	CsPbBr_3_ (505 nm)	-	360	5.47	0.14	[161]
2024	Inkjet printing	FA_0.8_Cs_0.2_PbI_3_/CsPbBr_3_/Cs_0.75_EA_0.25_PbBr_3_ (769/515/490 nm)	-	2800	14.3	2.59	[162]
2024	Crystal growth	CsPbI_3-x_Br_x_/ CsPbBr_3_/CsPbBr_3-x_Cl_x_ (625/512/490 nm)	-	400	7.6(R), 11.6(G), 4.3(B)	720(R), 1400(G), 1000(B)	[163]
2024	Inkjet printing	PEA_2_MA_3_Pb_4_I_13_	~7500	400	-	-	[164]
2025	Photolithography/ Focused ion beam etching	(FA, Cs)PbI_3_/(FA, Cs)Pb(Br,Cl)_3_/(FA, Cs)PbBr_3_/(Cs, FA, Rb)Pb(Br,Cl)_3_ (800/645/525/488 nm)	0.008	-	20.6(NIR), 12(R), 20.6(G), 12(sky-B)	40(G, @10 mA cm^− 2^)	[142]
2025	Photolithography	CsPbBr_3_ (516 nm)	~6.5	625	13.09	0.5	[165]
2025	Crystal growth	CsPbBr_3_/CsPb(Br,I)_3_ (516/640 nm)	20	100	-	-	[166]
2025	Nanoimprinting	FA_0.15_Cs_0.85_PbBr_3_ (517 nm)	~2.8	225	15.79	74.4	[167]
2025	Nanoimprinting	CsPbBr_3_ (460 nm)	~20	225	5.04	-	[168]

### Top-down Methods

#### Photolithography

Photolithography is a well-established semiconductor fabrication technique used to create fine patterns on films or substrates by direct photolithography or combined photomasks [175, 176]. The process typically involves spin-coating a photoresist onto the surface, followed by selective exposure to ultraviolet (UV) light through a patterned photomask [174, 177]. Depending on the type of photoresist used, exposure induces chemical changes that either increase solubility (positive photoresist) or promote polymerization and hardening (negative photoresist) [178, 179]. Commonly utilized resist types and corresponding parameters are summarized in Table 3 for comparison. Despite its precision and scalability, applying photolithography to perovskite materials presents significant challenges due to their intrinsic chemical and thermal sensitivity [180, 181]. Traditional lithographic processes rely on organic solvents and alkaline developers, which can dissolve perovskite layers, disrupt their structural integrity, and induce undesirable ion migration [182]. Meanwhile, high-temperature processing steps, typically around 100 °C, required for photoresist curing and resolution enhancement pose thermal stability challenges, as organic–inorganic hybrid perovskites tend to undergo decomposition or phase transitions within the 100–150 °C range.

**Table 3:** Summary of typical perovskite-compatible photoresist types and critical metrics

Resist type	Developer/process solvent	Typical bake/solvent exposure	PLQY retention (after a full cycle)	Typical minimum feature size of perovskite	Refs
Positive novolac/DNQ i-/g-line PRs (S1813, AZ series)	TMAH aqueous developers (AZ 300 MIF, MF-319)	~ 90 °C soft-bake; ~ 110 °C PEB (tool-dependent)	Significant weaken (directly exposed); safe (pattern-first, deposit-later)	≥ 10 µm	[147]
Negative epoxy PR (SU-8)	PGMEA (SU-8 Developer)	95 °C soft-bake; 150–200 °C PEB/hard-bake	Significant weaken (directly process on perovskite); generally used as mask	~ 1–3 µm (tool-dependent)	[172]
PMMA/ZEP EBL stacks (perovskite-compatible)	Non-polar / weakly polar developers (o-xylene, toluene; ZED-N50/amyl acetate; hexane rinse)	~ 90–100 °C	No weakening (orthogonal solvent set avoids perovskite attack)	~50 nm	[173]
Orthogonal photolithography	Chlorobenzene:hexane (≈1:3) or fluorous HFEs with fluorinated resists	≤ 100 °C	No weakening (minimal PL degradation reported when fully orthogonal)	~ 1–5 µm routinely; 5 µm RGB PeQD	[171, 172, 174]

**Fig. 6:**
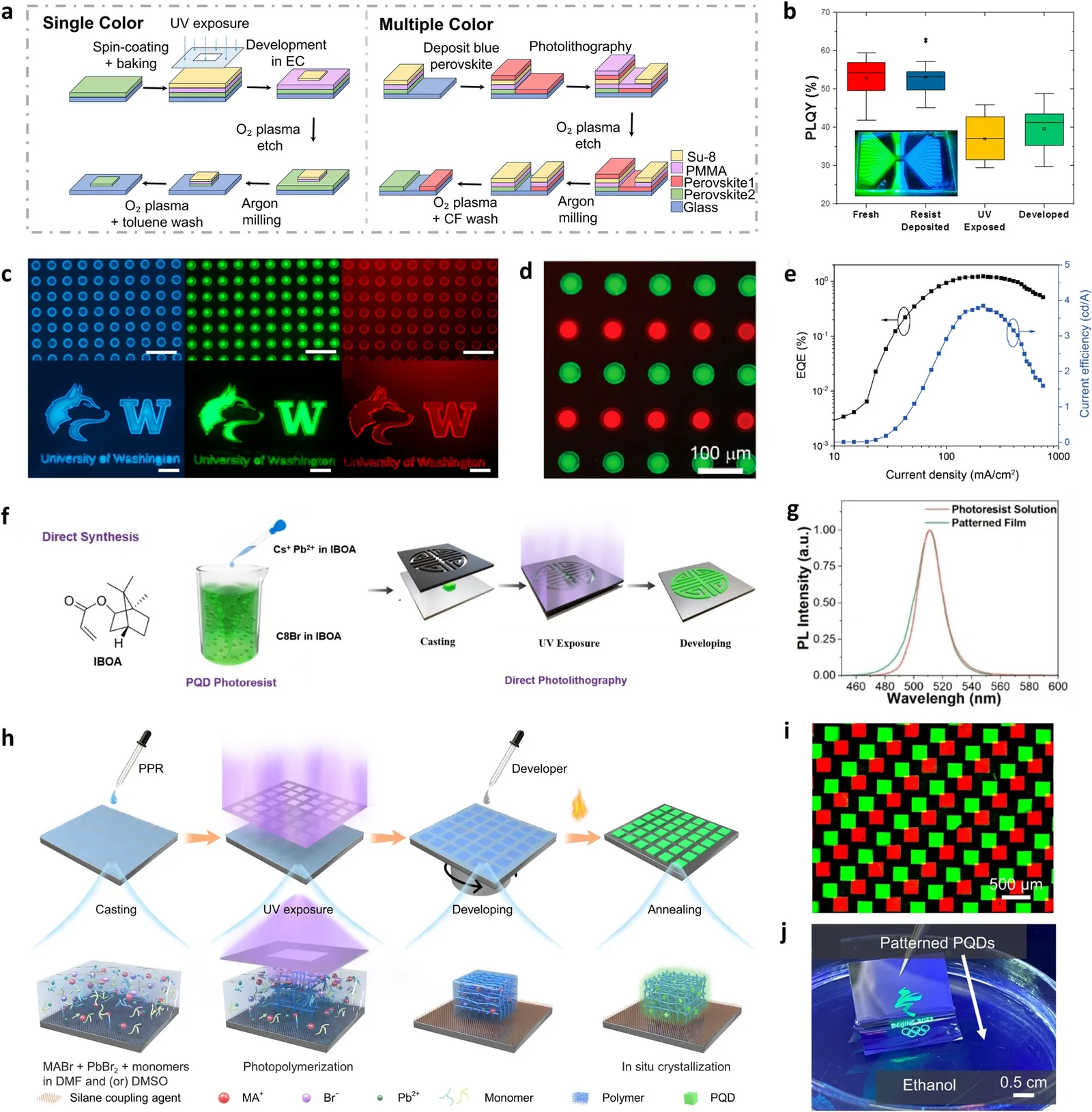
Photolithography strategy for perovskite patterns and PeQDs. **a** Multicolor patterning process by photolithography. **b** PLQY of perovskite sample under different processing conditions. Inset: green and blue color patterns [169]. **c** PL images of multicolor patterning (scale bar: 50 μm) and “University of Washington” logo (scale bar: 200 μm). **d** PL image of multicolor (red and green) pattern. **e** EQE and current efficiency verse current density of PeLEDs [147]. **f** The process of the direct synthesis method for PeQDs. **g** The PL spectra of photoresist solution and patterned film [170]. **h** Schematic of the direct in situ photolithography method using PPR, where annealing involves heating the samples to a precise temperature. **i** PL images of red and green patterns.** j** Photograph of a green logo immersed in ethanol under UV light [171]

To mitigate the detrimental effects of solvents and plasma exposure during photolithography, a stable protective layer—such as inorganic materials (e.g., SiO_2_ or SU-8) or resin-based coatings—is typically deposited or transferred onto the perovskite thin film prior to lithography and etching processes [183]. These encapsulation layers help preserve the integrity of the perovskite’s optoelectronic properties. Harwell et al. demonstrated SU-8 as a negative epoxy-based resist used to facilitate photolithography and polymethyl methacrylate (PMMA) as a removable protecting layer for the perovskite layer [169]. This two-layer protective strategy minimized damage to the perovskite layer during photolithography. The process involved exposing and developing the SU-8 to create a patterned template, followed by the deposition of different colored perovskite materials in the unexposed regions. This process is repeated multiple times, with each new layer of perovskite being selectively deposited in the unexposed areas, resulting in a multicolor pixel array (Fig. 6a). Figure 6b indicates that, with this multilayer protective structure, the PLQY of the perovskite film experienced minimal loss during processing (from 55 to 40%), demonstrating that the photolithography process does not significantly affect the optical properties of the perovskite. With this dual-layer protection structure, they achieved multicolor patterns (green and blue). Despite this success, the requirement for thick protective layers complicates the multicolor photolithography process, posing a significant challenge for fabricating high-quality multicolor patterns.

An alternative strategy involves the application of a photoresist or sacrificial layer on the substrate before perovskite deposition [176]. The perovskite is then grown or deposited on top of this protective layer, and patterning is achieved through a lift-off process or other similar techniques. This approach effectively avoids the direct etching of the perovskite layer, thereby minimizing the risk of material degradation and preserving the integrity of the perovskite’s optoelectronic properties. The key distinction between these two methods mentioned above lies in the sequence of deposition and patterning: the first method involves depositing the perovskite layer before setting the pattern on the template, while the second method deposits the perovskite after the template has been patterned. Using the lift-off approach, Zou et al. successfully fabricated multicolor patterns, including a “University of Washington” logo and red-green pixel arrays (Fig. 6c, d) [147]. However, during the lift-off process, the perovskite layer may adhere to the boundaries of the patterned material, leading to potential damage or loss. This is evident in the observed pattern defects at the boundaries, likely caused by the photolithography or lift-off process. The resulting devices achieved a maximum EQE of 1.24% (Fig. 6e), a current efficiency of 3.85 cd A^− 1^, and a luminance of 13,043 cd m^− 2^, which is relatively low compared to other perovskite patterning strategies. This performance gap can be attributed to two coupled factors. First, chemical incompatibility and residuals contamination: conventional resists, developers, and strippers can dissolve or reconstruct halide perovskites, leaving surface residues that quench PL [147]. A common workaround is to pattern the template first, then deposit the perovskite into the features, and finally peel or lift off the template, so the perovskite never contacts photoresist solvents or developers. Second, process-induced damage: oxygen-plasma cleans, plasma-enhanced chemical vapor deposition (PECVD) steps, and ambient O_2_/H_2_O exposure can extract halides, form Pb-oxide and vacancy defects, suppress PLQY, and thus reduce EQE [184]. Accordingly, oxygen plasma could be avoided, and all wet steps could be carried out under an inert atmosphere. For multicolor arrays, repeated photolithography and lift-off cycles, together with protective interlayers to shield prior perovskite layers from solvents and etchants, further increase process complexity and cross-contamination risk. While these techniques offer precise patterning capabilities, their limitations in terms of efficiency and process complexity underscore the need for further innovation in perovskite patterning strategies.

Photolithography also plays a critical role in the patterning of PeQDs, facilitating their spatially controlled formation and integration [185–187]. This technique enables both the direct conversion of perovskite precursor solutions into well-defined PeQD patterns and the realization of ligand- or polymer-assisted synthesis and patterning strategies, thereby achieving high-resolution and ordered structures suitable for optoelectronic applications [188, 189]. Recently, Zhou et al. [170] proposed a groundbreaking method for directly synthesizing perovskite quantum dot photoresists, significantly simplifying the photolithographic process. In this method, isobornyl acrylate (IBOA) effectively dissolved perovskite precursors and triggered photopolymerization upon UV exposure, producing high-brightness, high-stability PeQD patterns after development (Fig. 6f). Figure 6g demonstrates that the PL intensity of both the perovskite film and the perovskite photoresist solution remained mostly unchanged after the lithography, and the emission peak wavelength of the cured film matched that of the original PeQD photoresist. However, the precision of this method can be influenced by factors such as precursor concentration, photoresist thickness, and exposure conditions. Additionally, post-exposure development may introduce defects, limiting the overall quality of the patterned PeQD. In contrast, the ligand/polymer-assisted synthesis method enhances deposition selectivity and PeQD stability by incorporating functionalized ligands and polymer layers. This strategy not only improves the selective deposition of quantum dots but also stabilizes them against environmental degradation [170, 190].

A notable advancement is the development of a non-destructive technique that directly patterns perovskite precursors without exposing them to high-energy UV light or harsh solvents. Instead, PeQDs are generated in situ through annealing, ensuring uniform distribution and high optical quality (Fig. 6h) [171]. UV-activated lead bromide complexes act as catalysts for thiol-ene free-radical polymerization. Sulfur radicals generated during the process initiate polymer chain formation, while oxygen facilitates the regeneration of lead bromide complexes, ensuring sustained catalytic activity. Additionally, it supports the fabrication of multi-color PeQD patterns with customizable thicknesses up to 10 µm, making it suitable for red and green color displays (Fig. 6i). Furthermore, polymer encapsulation provides robust stability by protecting PeQDs from ion migration, crystal aggregation, and environmental factors such as moisture, oxygen, and heat (Fig. 6j). Despite these advantages, the ligand/polymer-assisted synthesis method introduces additional complexity due to the need for functionalized ligands and polymer layers. These components can affect the adhesion and distribution of quantum dots on the substrate, potentially leading to uneven deposition and impacting the final patterning quality and optical performance. Nevertheless, this method represents a significant step forward in achieving high-resolution, multicolor PeQD patterns for advanced display applications.

#### Laser/E-beam Lithography

Instead of utilizing conventional UV light, electron beam (e-beam) and laser irradiation have been explored as alternative patterning techniques for perovskite optoelectronic devices [194–200]. These methods eliminate the need for photomasks, enabling programmable, high-resolution, and highly flexible patterning processes on both flat and curved surfaces [199]. In e-beam lithography, a focused e-beam is used to expose the resist layer, creating a nanoscale pattern that serves as a mask for subsequent etching or material deposition, thereby defining the geometry of the perovskite layer [201]. While e-beam lithography offers excellent resolution down to the nanometer scale, its high cost and low throughput limit its practicality for large-area applications [202]. Laser lithography, on the other hand, replaces the electron beam with a focused laser and typically employs conventional photoresists instead of specialized e-beam resists, offering a faster and more cost-effective alternative [203]. However, its resolution is constrained by the laser spot size, typically on the micrometer scale [204, 205]. Depending on the interaction between the e-beam/laser and the perovskite material, these lithography strategies can be classified into two main categories: direct ablation, which physically removes material to create patterns, and photothermal-induced crystallization, which enables localized perovskite crystallization [206, 207]. Besides of perovskite patterning, these techniques can also be employed for fabricating structured substrates, photoresist molds, and even inducing selective crystallization in perovskite films [208].

**Fig. 7:**
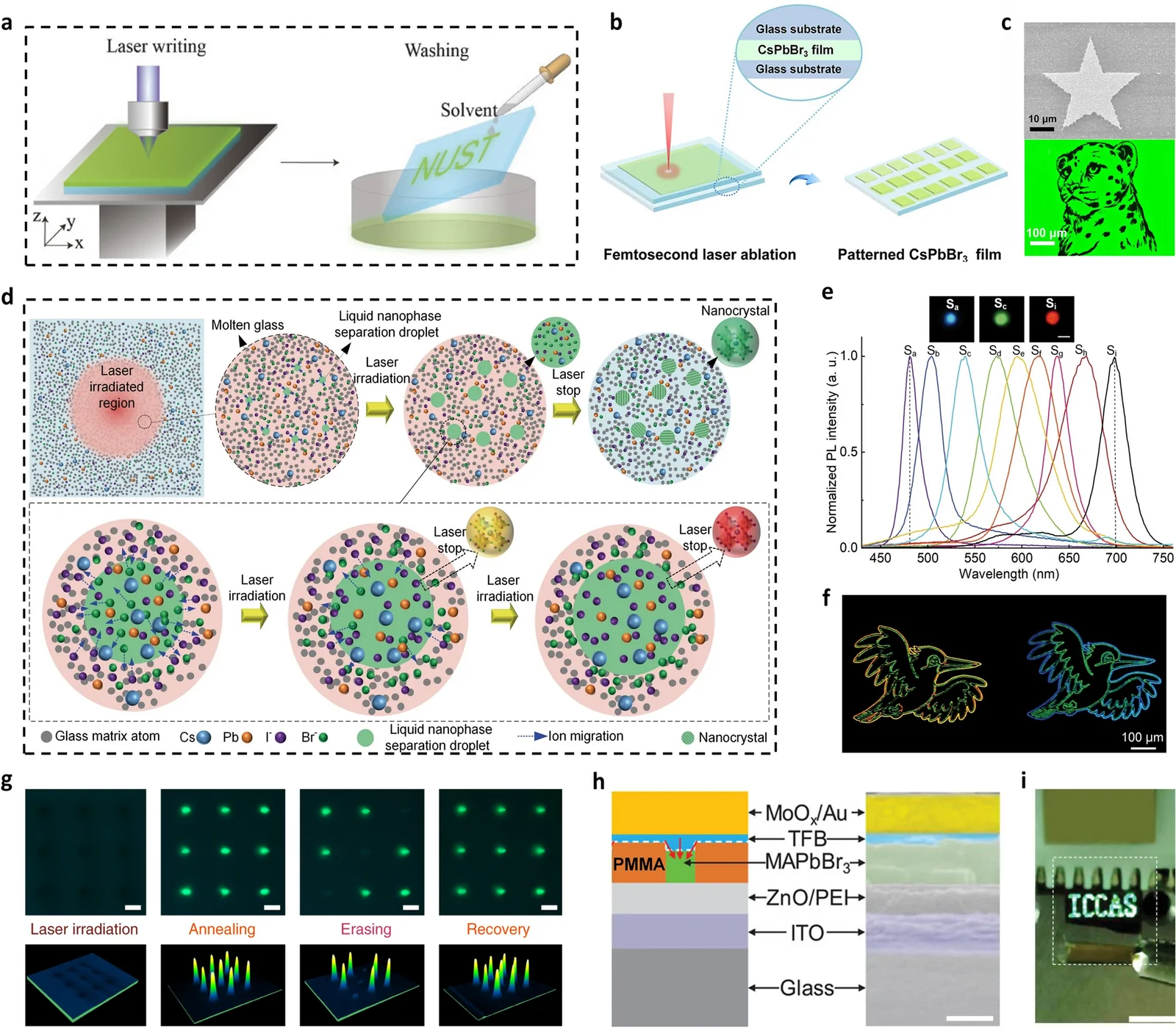
Laser/e-beam lithography strategy for perovskite patterns and luminescent applications. **a** Schematic illustration of laser-induced patterning process [191]. **b** Schematic illustration of patterned CsPbBr_3_ films fabricated via lamination-assisted femtosecond laser ablation (LA-FsLA). **c** High-magnification SEM image and PL image of the patterned CsPbBr_3_ film [152]. **d** Schematic of ultrafast laser-induced liquid nanophase separation and formation of CsPb(Br_1−x_I_x_)_3_ NCs in the Br^−^-I^−^-doped glass. **e** PL mappings and PL spectra of PeNCs written in the Cl^−^-Br^−^-I^−^ co-doped glass. S_a_ to S_i_ represent the PeNC samples written in the Cl^−^-Br^−^-I^−^ co-doped glass with different laser parameters. **f** PL images of the multicolor patterns produced with CsPb(Br_1−x_I_x_)_3_ NCs in the Br^−^-I^−^ doped glass and CsPb(Cl_1−x_Br_x_)_3_ NCs in the Cl^−^-Br.^−^ doped glass [192]. **g** Optical images (upper) and readout signal intensity mapping images (lower) of a CsPbBr_3_ QD array during the erasing–recovery processes under UV light (scale bars, 100 μm) [193]. **h** Schematic structure and cross-sectional SEM image of fabricated perovskite micro-LED device (scale bars, 100 nm). **i** Display of “ICCAS” characters using perovskite micro-LED arrays (scale bars, 10 mm) [145]

In 2017, Chen et al. [191] introduced an innovative application of laser direct writing (LDW) for patterning all-inorganic perovskite quantum dots (CsPbBr₃), marking its first use in this context. Without the requirements of vacuum environments or complex post-processing, LDW simplifies the fabrication process into three efficient steps: spin-coating quantum dots, laser writing, and solvent washing (Fig. 7a). By adjusting parameters such as laser spot size, scanning speed, and energy, LDW achieves controllable pattern resolutions ranging from 3.3 to 100 μm, making it suitable for both microscale and large patterns. Additionally, LDW not only defines patterns but also optimizes the morphology and PL intensity of QDs, enhancing both brightness and morphology quality. A fabricated large-scale 100 mm × 100 mm pattern demonstrates its potential for applications in quantum dot displays and optical storage.

Due to its high precision, rapid processing, and programmable patterning capabilities, femtosecond laser ablation (FsLA) has emerged as a promising method for fabricating perovskite films with resolutions down to the micrometer scale and 3D patterning of PeQDs [209]. Compared to conventional laser writing, which typically uses continuous wave or nanosecond pulsed lasers, FsLA achieves higher spatial resolution and minimizes thermal damage by confining energy deposition within extremely short pulses. This enables precise ablation without affecting surrounding areas, preserving perovskite material quality [210]. Based on this strategy, Liang et al. [211] utilized a focused NIR femtosecond laser (1030 nm) to selectively ablate perovskite material through lattice melting and Coulomb explosion, achieving fine 2D perovskite patterns with resolution as high as 1.78 µm. These patterns featured diverse geometries, including portraits, microline arrays, and microsquare grids, all fabricated without introducing additional pinholes or cracks. Utilizing this FsLA strategy, they developed fluorescent anti-counterfeiting labels capable of retaining their properties even in environments with 96% relative humidity, highlighting their potential for high-security authentication applications. To further enhance device integration, they introduced a sandwich-laminated structure, in which perovskite films were enclosed between two glass layers to prevent debris contamination during processing (Fig. 7b) [152]. As shown in Fig. 7c, this approach yielded complex perovskite patterns with well-defined edges and uniform PL properties, which were successfully incorporated into PeLED devices, demonstrating high-quality emission characteristics.

In contrast to conventional thermal annealing, which lacks spatial control over crystallization, laser-induced photothermal heating provides a precise means of inducing local perovskite crystallization [212]. Femtosecond lasers, known for their ability to create three-dimensional photonic structures with high spatial resolution, achieve this through rapid atomic redistribution enabled via nonlinear optical processes [213]. Using femtosecond laser-induced liquid nanophase separation, Sun et al. achieved 3D direct lithography of PeNCs embedded in glass with tunable photoluminescence from 480 to 700 nm [192]. As illustrated in Fig. 7d, localized laser heating and pressure gradients drive halide migration, initially forming Br-rich regions (~ 520 nm green emission), with prolonged exposure inducing I⁻ incorporation, shifting the red emission (~ 690 nm). The integration of Cl⁻ ions further enables precise emission tuning, enabling the creation of multicolor perovskite patterns like CsPb(Br_1−x_I_x_)_3_ and CsPb(Cl_1−x_Br_x_)_3_ NCs, suitable for diverse optoelectronic applications (Fig. 7e, f). Utilizing 3D laser printing, Huang et al. also demonstrated the reversible in situ formation of MHP QDs embedded within a transparent glass matrix [193]. Compared to solution-processed QDs, this approach offers enhanced stability and seamless device integration. The multiphoton absorption effect of the laser effectively induces nucleation within localized regions, enabling precise 3D patterning. As illustrated in Fig. 7g, patterns were selectively erased and re-formed through laser-induced decomposition and subsequent low-temperature annealing, allowing for multiple processing cycles while maintaining the QD structure and luminescent properties. This method paves the way for high-precision, reconfigurable optoelectronic devices.

Beyond direct lithography, e-beam lithography has been employed to fabricate perovskite-based display components. Using an e-beam lithography patterned PMMA resist, Wang et al. reported a wettability-guided screen-printing technique to produce multicolor perovskite microdisk arrays with precise geometry [145]. This technique exploits wetting/dewetting mechanisms, where the surface energy contrast created by the EBL-patterned resist directs the placement of perovskite ink, enabling controlled deposition of microdisk arrays. The resulting microstructures exhibited whispering gallery mode (WGM) lasing with tunable colors across the visible-to-near-infrared spectrum, making them suitable for high-resolution display applications. Besides, they successfully fabricated micro-LEDs with a residual PMMA-induced "current-focusing" architecture within the transporting layer of TFB as illustrated in Fig. 7h, enhancing charge injection efficiency for practical display integration (Fig. 7i). However, poor crystallization control and insufficient grain boundary passivation in perovskite films remained significant challenges, ultimately limiting the EQE of fabricated PeLEDs to ~ 0.1%.

#### Nanoimprinting

Nanoimprinting is a cost-effective, high-resolution, and high-throughput lithography technique that enables direct replication of nanoscale features onto substrates using a soft stamp with an inverse pattern [214, 215]. This method is widely employed in perovskite optoelectronic devices, either by imprinting patterns onto an electron transport layer (e.g., TiO₂ or ZnO) or directly onto the perovskite active layer [42, 215–218]. In the case of perovskite films, a thin layer of perovskite precursor material is first deposited onto a substrate, followed by pressing a pre-patterned mold—typically made of chemically inert, low surface energy materials such as silicon, quartz, or polydimethylsiloxane (PDMS)—under controlled pressure and temperature conditions [219, 220]. Solvent evaporation and directional deformation guide crystallization into the mold geometry, followed by thermal or chemical curing. Imprint quality depends on precursor viscosity, mold-substrate surface energy, applied pressure, temperature, and solvent evaporation rate. Pattern formation is governed by capillary filling and viscous flow, followed by rapid solidification to retain shape. Feature resolution can reach ultra-high values (about 20 nm), but is limited by mold fidelity, material relaxation, and capillary-driven reflow [221]. In contrast with photolithography, nanoimprinting enables high-resolution pattern formation without the diffraction limit of light, making it particularly suited for fabricating surface-relief quasi-3D structures and compatible with flexible substrates due to its low-temperature and low-pressure process conditions [214, 222]. However, nanoimprinting strategy still suffers from several problems, including materials compatibility issues, as perovskite precursors are highly sensitive to temperature variations. Additionally, controlling the material flow within the mold is difficult, often resulting in non-uniform pattern formation. Large-area uniformity and alignment remain critical issues, and scaling up the process for industrial applications requires precise control over imprinting parameters, multiple processing steps, and optimization of curing conditions [223]. Despite these issues, nanoimprinting remains a promising technique for fabricating high-resolution perovskite films, offering excellent patterning fidelity for advanced optoelectronic applications.

**Fig. 8:**
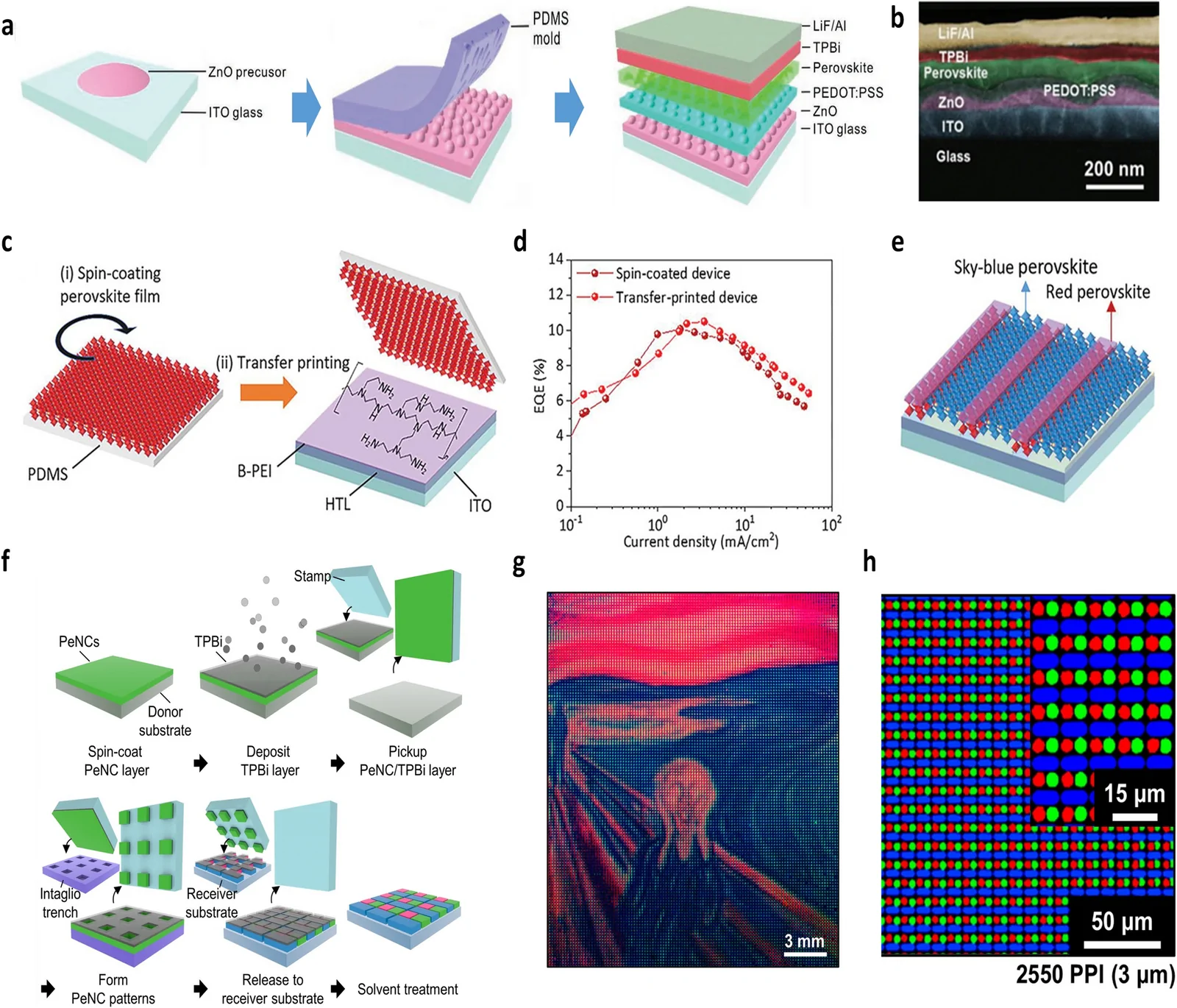
Nanoimprinting for PeLEDs. **a** Schematic illustration of the fabrication process of CsPbBr_3_ PeLEDs with imprinted nanostructure. **b** Cross-sectional SEM image of a nanoimprinted PeLED [144]. **c** Schematic illustration of transfer-printing process of perovskite films. **d** EQE of transfer-printed and spin-coated red PeLEDs. **e** Schematic illustration of the structure of a transfer-printed white emission perovskite film [153]. **f** Schematic illustration of the double-layer transfer printing process with RGB pixelated arrays of PeNCs. **g** PL image of transfer-printed RGB PeNC pixelated patterns displaying *The Scream* by Edvard Munch. **h** Fluorescence microscopic image of the pixelated RGB PeNC patterns with 2550 PPI resolution [34]

Although nanoimprinting has been extensively utilized in fabricating patterned OLED [224], QDLED [225], solar cell [223, 226], photodetector [227], and other optoelectronic devices [154, 228–230], its application to PeLEDs began only recently in 2019. Shen et al. [144] employed bioinspired moth-eye nanostructures (MEN) at the front electrode/perovskite interface to enhance the outcoupling efficiency of waveguided light, by utilizing nanoimprinting to fabricate CsPbBr₃-based green-emitting PeLEDs. As illustrated in Fig. 8a a sol–gel-derived ZnO precursor was spin-coated onto an ITO glass, followed by imprinting with a PDMS mold containing the MEN pattern. After conformal pressing and post-annealing at 150 °C, subsequent layers of PEDOT: PSS, CsPbBr_3_ perovskite, TPBi, LiF, and Al were sequentially deposited to complete the PeLED device. The cross-sectional scanning electron microscope (SEM image) in Fig. 8b shows the patterned ZnO layer with an inverse-patterned CsPbBr₃ layer. This nanoimprinted structure significantly reduced Fresnel reflections, enabling the PeLED to achieve an EQE of 20.3%, which increased to 28.2% with a half-ball lens. To address the challenge of fabricating ultrahigh-resolution display, Kim et al. developed a micropatterning method utilizing capillary force lithography, achieving 10-µm-scale micropatterns [154]. This method incorporated poly(ethylene oxide) (PEO) and an O₂ plasma-treated PDMS mold to suppress self-aggregation and enhance the heterogeneous nucleation of the perovskite precursor, resulting in highly uniform microarrays. The fabricated micro-PeLEDs exhibited a characteristic red emission at 650.9 nm and an EQE of 5.9%.

While nanoimprinting offers high-precision patterning, it faces inherent challenges in controlling the nucleation and crystallization dynamics of perovskite materials. The confinement of precursors within nanoscale cavities can lead to non-uniform grain growth and defect formation due to uneven solvent evaporation and stress accumulation. Moreover, nanoimprinting is typically limited to single-layer patterning and lacks the flexibility for spatially selective deposition, making it unsuitable for multicolor pixel fabrication [231]. To address these limitations, transfer printing has emerged as a complementary technique, allowing crystallization and patterning to be performed independently. By first developing high-quality perovskite films or nanocrystal layers and then transferring them onto target substrates, this technique minimizes defects and enables advanced pixel architectures [232]. Transfer printing typically employs soft, viscoelastic stamps (e.g., PDMS), which facilitate conformal contact and/or separation through controlled adhesion modulation [233]. Recent studies have demonstrated laser- or thermally triggered release protocols that tune interfacial adhesion, enabling precise pattern transfer with sub-micron alignment accuracy [232, 234, 235]. For example, double-layer printing with organic charge transport layers has achieved ultrahigh resolution up to ~ 3 μm RGB pixels (≈2,550 pixels per inch) without internal cracking and 100% pattern transfer yield on flexible substrates [34]. The underlying physics involves carefully balancing the work of adhesion, viscoelastic stamp mechanics, and stamp–substrate separation kinetics to favor either pickup or release. Thermal and laser triggers temporarily alter adhesion properties, while peeling dynamics define whether the film remains on the stamp or transfers to the substrate [231, 236–240]. By decoupling film quality control from pattern alignment, transfer printing surmounts nanoimprinting’s limitations—enabling large-area multicolor integration, high-resolution patterning, and compatibility with flexible or curved substrates—making it a versatile and scalable route for next-generation PeLED devices [241].

Li et al. developed a reliable mass transfer printing technique for perovskite films and nanostructures, achieving comparable results to optimized spin-coated films in terms of surface morphology, composition, and optoelectronic properties [153]. The method enabled high-resolution patterning up to 1270 pixels per inch (PPI). A key innovation was the introduction of an ultrathin branched-polyethyleneimine(B-PEI) chemical bonding layer, which effectively resolved electrical contact issues between the transferred perovskite films and the receiving substrate (Fig. 8c). Using this method, they fabricated PeLEDs with decent EQEs of 10.5% (red) and 6.7% (sky-blue) (Fig. 8d). Additionally, they demonstrated a novel white PeLED structure by laterally aligning red and sky-blue perovskite microstripes via multiple transfer steps (Fig. 8e). To further improve performance, alignment accuracy can be improved using an automated alignment system that utilizes an optical microscope with computer vision algorithms and a motorized microstage to achieve placement accuracy below 40 nm, or microstripes can be transferred onto the TFT to independently control the drive voltage for each color, eliminating the need for a PMMA layer [242].

A key limitation of conventional dry transfer printing is the formation of internal cracks in the PeNC film during transfer. To address this, Kwon et al. demonstrated a double-layer transfer printing approach, incorporating both PeNCs and an organic charge transport layer [160]. Specifically illustrated in Fig. 8f, this method begins by simultaneously picking up spin-coated PeNCs and thermally evaporated TPBi using a viscoelastic PDMS stamp. The assembly is then gently placed onto an intaglio trench, allowing selective removal of the double-layer structure due to the surface energy contrast between the PDMS stamp and the trench. After construction of RGB subpixels by repeating the printing method, solvent treatment is conducted to enhance the junction properties between the emitting and charge transport layers, optimizing performance for LED applications. This double-layer transfer printing technique enabled the fabrication of RGB pixelated PeNC patterns with an ultrahigh resolution of 2550 PPI and a near-perfect transfer yield (~ 100%) (Fig. 8g, h). Moreover, PeLEDs with transfer-printed active layers demonstrated outstanding EQEs of 15.3% for red devices and 14.8% for green devices, as well as synthesis of ultrathin multicolor PeLEDs that can operate on human skin. These results indicate double-layer transfer printing holds significant promise for next-generation high-resolution displays and wearable optoelectronic devices.

### Bottom-up Methods

#### Patterned Crystal Growth

Among the various approaches for synthesizing perovskite films, crystal growth methods have emerged as crucial strategies for optimizing material performance [247, 248], stability [249, 250], and scalability [251, 252]. From a thermodynamic perspective, crystal growth is driven by the minimization of the system’s free energy. During the transition from a disordered precursor state to an ordered crystalline structure, the reduction in Gibbs free energy facilitates nucleation and subsequent crystal growth. Controlling surface energy, crystallization rate, and phase stability—particularly through modifications that increase the Goldschmidt tolerance factor or reduce crystal size—further optimizes the energy landscape, enabling the formation of high-quality films with suppressed defect densities and enhanced photovoltaic performance [253, 254]. The growth of perovskite patterns is typically achieved by using templates with specific shapes and structures to guide the perovskite material to grow in a particular morphology and arrangement on the template surface or structure, resulting in highly oriented displays [255]. Alternatively, by hydrophilic/hydrophobic treatments to the substrate and large-area film fabrication methods such as blade-coating, growth can be localized to specific regions, enabling the formation of arrayed perovskite crystals for patterning in display applications [256]. These strategies enable the fabrication of high-quality perovskite materials with fewer defects, reduced non-radiative recombination, and improved carrier mobility [257]. By precisely controlling growth conditions, the size and morphology of the crystals can be tailored, leading to more efficient and stable devices [258].

**Fig. 9:**
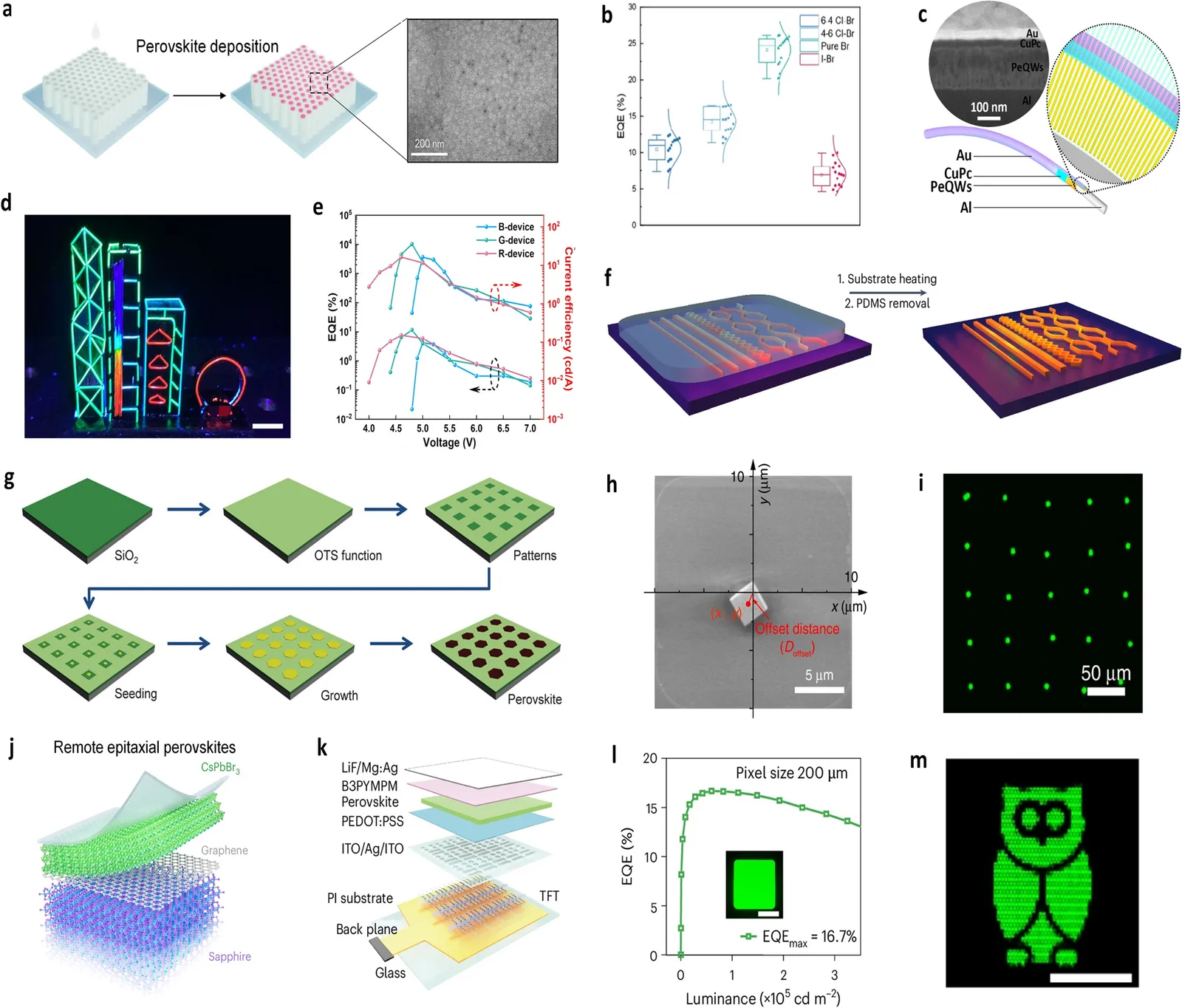
Crystal growth strategy for PeLEDs. **a** Scheme of perovskite growth process on PAM template. **b** Distribution of EQEs of four different colored PeQWs-based LEDs [57]. **c** Schematic structure images of Fi-LEDs fabricated by crystal growth using PAM template. **d** Three colors of Fi-LEDs with freestanding structures. **e** EQE–current efficiency–voltage curves of RGB LED devices [163]. **f** Schematic illustration of patterned perovskite structures by the pseudomorphosis under decreased crystallization rate [243]. **g** Scheme of perovskite crystallization on a hydrophobically treated substrate [244]. **h** Coordinate system of a perovskite microplate in a hydrophilic window. **i** Fluorescence micrography of MAPbBr_3_ array under UV light [245]. **j** Schematic of perovskite epitaxial growth on a sapphire substrate with a graphene interlayer, resulting in a free-standing film after release. **k** A schematic of an active-matrix perovskite micro-LED display. **l** EQE–luminance curves of perovskite micro-LEDs with 200 μm pixel size. **m** Static images displayed by perovskite micro-LEDs (scale bar: 2 mm) [246]

The surface-confined crystal growth typically involves a confinement depth of around 100 nm, which is relatively small compared to macroscopic dimensions and can therefore be regarded as a surface treatment. In this strategy, perovskite precursors are confined to pre-defined surfaces or voids on the substrate, often leading to superior crystallinity relative to thin films produced by spin-coating. Fan et al. reported the fabrication of crystalline all-inorganic perovskite quantum wire arrays with ultrahigh density and exceptional uniformity using porous alumina membranes (PAMs) with ultra-small pore sizes (6.4 nm) as templates, eliminating the need for organic ligands and antisolvents (Fig. 9a) [57]. The PeLEDs based on these quantum wire arrays, incorporating a dual-functional molecule as both a passivation layer and a hole transport layer, achieved EQEs of 9.97%, 12.41%, 16.49%, and 26.09% for pure-red, blue, sky-blue, and green emissions, respectively (Fig. 9b). Flexible (15 × 15 mm^2^) and large-area (30 × 35 mm^2^) PeLED device based on PAMs demonstrated stable EL emission. Since commonly employed patterning and etching techniques are designed to obtain perovskite patterns on two-dimensional surfaces, achieving perovskite patterns on curved substrates represents a significant challenge. To address this, perovskite quantum wires (PeQWs) have been integrated into aluminum fibers through a roll-to-roll solution-coating technique, which allows for the growth of patterned perovskite material on the curved surface of the fibers [163]. As illustrated in Fig. 9c, the thin aluminum fibers, due to their malleability and plasticity, are well-suited for both 2D and 3D architectures. This is exemplified by a 3D multicolor “night scene” of Victoria Harbour, demonstrating their versatility in creating complex freestanding and multicolor display structures (Fig. 9d). The PeQWs exhibited nearly 90% PLQY, and the resulting fiber LEDs (Fi-LEDs) achieved a high EQE of 15.2% (Fig. 9e). These Fi-LEDs maintained excellent stability under strong external interference, such as twisting and water immersion, demonstrating excellent stability. Despite these advancements, the need for pre-treatment of the Al_2_O_3_ substrate to form PAM templates adds complexity to the process, potentially hindering industrialization. Additionally, SEM images indicate that the hole arrangement in the PAMs was less regular and uniform than depicted in the schematic (Fig. 9a), which could impact device performance.

Focusing on perovskite crystal growth with different morphologies, space-confined perovskite crystal growth utilizes preconfigured templates to guide perovskite growth within defined spaces, forming structures with predetermined shapes. Kędziora et al. [243] demonstrated a template-assisted method for fabricating large-scale waveguide perovskite crystals with arbitrary predefined geometries. As illustrated in Fig. 9f, a PDMS mold is created by polymerizing on a GaAs master template, transferred to a new substrate, and filled with a perovskite solution under cooled conditions to control crystal growth. Heating facilitates solvent evaporation and crystallization, after which the mold is removed. SEM images show that different predesigned crystal geometries with a height of 600 nm can be achieved. This crystallization method reduces production costs and process complexity by eliminating the need for atomically flat substrates or complex chemical treatments. The precision and shape of the template design are crucial to the final morphology of the crystals, and inaccuracies or defects in the template can lead to uneven crystal growth or shape deviations. However, the process of fabricating and using templates remains relatively complex, particularly when dealing with large-area or uniquely shaped crystals. Additionally, residual material from template removal may affect crystal quality and device performance.

Another approach is the surface dewetting-assisted method, which utilizes hydrophobic substrate treatment to induce patterned perovskite crystallization [244, 259, 260]. During annealing or solvent evaporation, surface dewetting causes phase separation, forming discrete droplets or isolated patches [261]. Wang et al. [244] reported this method by treating a SiO_2_ substrate with octadecyltrichlorosilane (OTS) to create hydrophilic and hydrophobic regions, enabling controlled nucleation and growth of perovskite crystals (Fig. 9g). The nucleation and growth of PbI_2_ microplates were precisely controlled in pre-defined patterns. Xu et al. [245] further advanced this technique by combining space confinement with antisolvent-assisted crystallization (SC-ASC) to fabricate high-quality perovskite single-crystal arrays on-chip. The SC-ASC method, which integrates traditional photolithography with antisolvent crystallization, achieves precise control over crystal position, size, and orientation, enabling high-resolution arrays with tunable pixel dimensions (ranging from 2 to 8 µm) and less than 10% positional deviation (Fig. 9h). This method allows flexible pattern customization and uniformity across large arrays (Fig. 9i). However, the need for hydrophobic substrate treatment adds complexity, and achieving uniform crystallization remains a challenge.

In addition to surface confinement, remote epitaxy growth has been proposed as an innovative technique. Wu et al. [246] introduced a sub-nanometer graphene interlayer between the substrate and the perovskite film to achieve remote epitaxial growth with relaxed strain (Fig. 9j). By remote epitaxial crystalline, large-area (400 mm^2^), grain-boundary-free, and purely epitaxially oriented perovskite films were fabricated, which were seamlessly integrated into micro-LED devices. Photolithography was employed to isolate each perovskite pixel, approaching 10 μm resolution without detectable damage. Figure 9k illustrates the device configuration, where thin-film transistors control each perovskite pixel independently. These devices demonstrated a high EQE of 16.7% with 200 μm pixels and a brightness of 4.0 × 10^5^ cd m^− 2^, with a minimum pixel size of 4 μm (Fig. 9l). The display can show static images with uniform brightness (Fig. 9m), demonstrating a promising route for achieving high-performance micro-LED displays with fine resolution and exceptional brightness.

#### Inkjet/EHD Jet Printing

**Fig. 10:**
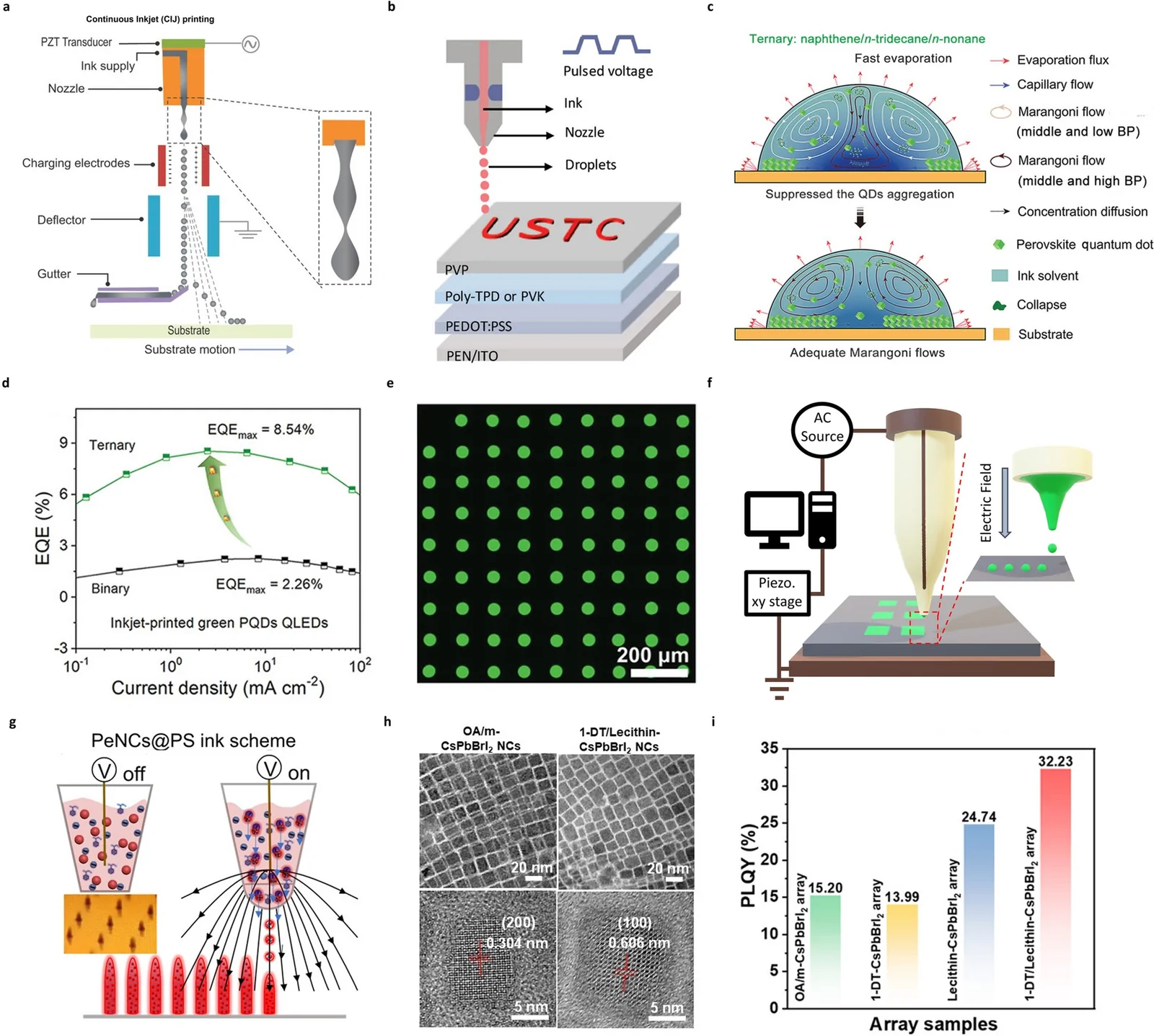
Inkjet and EHD jet printing strategy for perovskite and light-emitting applications. **a** Schematic diagram of CIJ printing process [270]. **b** Structure setup of perovskite inkjet printing system [162]. **c** Schematic illustration of solvent flow, evaporation process, and self-assembled formation. **d** EQE of an inkjet printed PeLED based on ternary ink [157]. **e** PL spectra of PeQDs patterns [155]. **f** Structure illustration of EHD jet printing system [280]. **g** Schematic illustration of printed PeNC arrays from PeNCs@PS ink. The inset displays microscope of printed arrays [281]. **h** TEM images of PeNCs with corresponding ligands. **i** PLQY of OA/m-CsPbBrI_2_ array, 1-DT-CsPbBrI_2_ array, lecithin-CsPbBrI_2_ array, and 1-DT/lecithin-CsPbBrI_2_ array [282]

Inkjet printing offers a versatile and scalable approach for depositing perovskite patterns at desired locations, making it suitable for LED displays and large-area devices fabrication [155, 262–266]. Unlike conventional deposition methods, inkjet printing generates minimal material waste, as the precursor ink is directly ejected from the nozzle onto the target substrate, where in situ crystallization occurs to form a perovskite film or pattern [267]. Additionally, inkjet printing is compatible with flexible electronics, facilitating the fabrication of bendable or foldable devices [150]. Inkjet printing enables layer-by-layer printing, allowing for the precise assembly of multiple device components, from LED electrodes to the perovskite emission layer [268]. There are 2 main droplet generation mechanisms in inkjet printing: continuous inkjet (CIJ) printing and drop-on-demand (DOD) inkjet printing [269]. In CIJ printing, a liquid column is formed under pressure and breaks apart into a continuous stream of droplets due to Rayleigh–Plateau instability (Fig. 10a) [270].

CIJ printing faces the risk of ink contamination due to the recycling of droplets and has lower resolution [271]. In contrast, DOD printing utilizes a printhead connected to an ink reservoir, where small, precise droplets (ranging from 1 to 100 picoliters) are ejected on demand via thermal or piezoelectric actuation [272, 273]. Miniaturized thermal DOD systems are commonly used for home applications, while piezoelectric DOD systems are preferred in industrial and material science printing due to their ability to modulate droplet volume and velocity. This is essential for stable, high-frequency inkjet processes with consistent droplet sizes. Most studies on inkjet printing for perovskite devices also use piezoelectric DOD systems, where the piezo actuator deforms to modulate chamber pressure, ejecting ink filaments via nozzle contraction [270, 274, 275]. Droplet detachment occurs through “end-pinching,” which is influenced by the rheology of the fluid, governed by the Ohnesorge, Reynolds, and Weber numbers [276, 277]. The governing dimensionless groups are the Reynolds number *Re* = *ρvL/μ*, the Weber number *We* = *ρv*^*2*^*L/σ*, and the Ohnesorge number *Oh* = *μ*/$\sqrt{\rho \sigma L}$ [278, 279]. Neck thinning and final pinch-off follow capillary–inertial scaling at low *Oh* and viscous–capillary scaling at high *Oh*, while stable DOD operation is typically achieved in transitional windows of *Re* and *We* with *Oh* ≈ 0.1–1 [269]. A stable end-pinching process requires fluid properties within transitional regimes, balancing viscosity, surface tension, and inertial forces.

Since the pioneering works of Hermerschmidt et al. (2020) using ionic perovskite precursors and Li et al. (2020) employing pre-synthesized PeQDs in inkjet printing formulations, both strategies have been established as fundamental approaches for PeLED fabrication [146, 283]. However, a major challenge in inkjet printing is the “coffee ring” effect, which impacts film morphology and uniformity. When a droplet is deposited onto a wetting substrate, solvent evaporation occurs more rapidly at the edges than at the center, generating microflows that transport solutes to the droplet perimeter. As the solvent continues to evaporate, solute accumulation at the edges leads to ring-like deposition patterns, deteriorating film uniformity and device performance.

To address this issue, many studies have focused on multisolvent strategies to suppress the coffee ring effect and improve PeLEDs performance. As shown in Fig. 10b, Xiao et al. [162] developed highly efficient, flexible, and large-area PeLEDs via inkjet printing by engineering surface wettability and optimizing solvent compositions. A double-hole transport layer and a wetting interface layer (PVP) were introduced to improve substrate wettability, while a binary-solvent system was employed to suppress solute migration and weaken capillary flow, thereby minimizing coffee ring formation. Additionally, the binary-solvent system created a surface tension gradient that induced recirculating Marangoni flow, leading to more uniform solute deposition. This approach enabled the fabrication of inkjet-printed PeLEDs with an EQE of 14.3%, representing a significant improvement in efficiency. The method also demonstrated scalability, achieving a uniform 2800 mm^2^ PeLED device and patterned structures such as the "USTC" logo. Further advancing ink formulation, Zeng et al. [157] introduced a ternary halogen-free solvent ink composed of naphthane, n-tridecane, and n-nonane, significantly improving printing performance and film quality compared with a binary-solvent system (Fig. 10c). The Marangoni effect, as a key interfacial phenomenon governing fluid dynamics in inkjet-printed droplets, deserves further elaboration on its intrinsic mechanism and regulatory pathway in PeLED fabrication [284]. Specifically, the surface tension gradient driving this effect originates from the differential evaporation rates of multi-solvent components: in binary systems, the preferential volatilization of the low-surface-tension solvent creates a local surface tension discrepancy between the droplet edge and center [285, 286]. This gradient triggers Marangoni flow directed from the low-tension edge to the high-tension center, which counteracts the outward capillary flow that dominates coffee ring formation—effectively redistributing aggregated PeQDs from the droplet periphery back to the bulk, thereby homogenizing the film [157]. Notably, the intensity of Marangoni flow is synergistically determined by the surface tension difference between solvents (Δγ) and their volatility mismatch (Δ*Pvap*): a larger Δγ enhances the driving force, while an appropriate Δ*Pvap* avoids overly rapid gradient decay caused by excessive volatilization of the low-surface-tension component [287]. In ternary halogen-free systems like naphthane/n-tridecane/n-nonane inks, the introduction of a third solvent enables more precise modulation of this effect: by tuning the content of n-nonane, the dynamic evolution of the surface tension gradient can be regulated—slowing the rate of gradient establishment to match the PeQD crystallization kinetics, and preventing the attenuation of the Marangoni effect due to premature depletion of volatile components [21, 288]. By using this ternary solvent system, green PeLEDs exhibited an EQE of 8.54% and a maximum brightness of 43,883.39 cd m^− 2^, far surpassing the binary solvent-based PeLEDs (EQE of 2.26%) (Fig. 10d). Moreover, this approach enabled the fabrication of high-resolution perovskite arrays with feature sizes as small as 45 μm (Fig. 10e).

Beyond the major challenge, coffee-ring effect, microscale patterning introduces additional constraints. Even when using 1–3 pL nozzles, the printed wet spot typically spans about 10–30 μm on low-energy surfaces, and subsequent spreading and merging push the practical pixel pitch above roughly 20–30 μm unless strong confinement is applied [289]. Serial, pass-by-pass printing also accumulates overlay errors across HTL, perovskite emission layer, and ETL stacks, and these errors become more pronounced on banked topographies [290]. Hygroscopic precursors and fast-evaporating co-solvents can crystallize at the nozzle tip, intermittent printing aggravates this crusting [275, 291]. These interactions swell seals or leave metal-salt residues that poison the orifice, which causes drift in drop volume and velocity and ultimately degrades pixel fidelity, yield, and device metrics.

EHD jet printing is an advanced printing technique capable of producing high-resolution perovskite patterns beyond the limits of conventional inkjet printing [292–297]. Unlike inkjet printing, which relies on thermal or piezoelectric mechanisms, EHD jet printing utilizes an electric field established between the nozzle and the substrate to control ink ejection and deposition [295, 298]. When the field is sufficiently strong, the liquid meniscus at the nozzle tip deforms into a Taylor cone [299, 300]. At the cone apex, the normal Maxwell stress (∝ εE^2^) counterbalances surface tension (∝ γ/R), and once the electric pressure exceeds the capillary pressure, a thin, charged microjet or a train of fine droplets is emitted [301, 302]. In the steady cone-jet regime, the stability limit is fully determined by the surface tension *γ*, the electric stress *ε*E^2^ and 2 geometric parameters of the droplet, so the printed droplet size or line width is set primarily by field strength and ink properties rather than by the nozzle size [303, 304]. As a result, features 10–100 × smaller than the nozzle are routinely achieved, and perovskite patterns with ≈1 µm resolution have been reported [155, 305]. Field-driven scaling further implies that increasing voltage, increasing ink conductivity, and reducing flow rate each tend to shrink the emitted jet or droplet, while viscosity and surface tension set stability windows for maintaining a steady cone-jet (Fig. 10f) [280, 295, 306].

For micro-LEDs with pixel pitches of roughly 10–30 µm, EHD jet printing enables deterministic placement of emitters with narrow linewidths and sharp edges, which suppresses optical and electrical crosstalk across pixel boundaries [297]. The electrically focused ejection reduces splashing and coffee-ring artifacts because the charged cone jet and electric field guided landing in EHD lower the effective Weber number *We* and suppress rim detachment, thereby improving uniformity within confined apertures [307, 308]. Because the electric field lines can be shaped by the device stack and biasing, EHD jet printing remains effective over passivation layers and mildly nonplanar topographies, aiding integration with dielectric isolation and thin-film interconnects. At the nanoscale, the decoupling of feature size from nozzle diameter allows sub-micrometer emitters, nanoribbons, and dense interconnects to be written directly, supporting ultra-high-PPI layouts and compact pixel apertures for next-generation displays and on-chip optoelectronics.

The earliest report of EHD jet printing can be traced back to 2007, showcasing the feasibility of using electric fields to generate droplets significantly smaller than nozzle diameters [292]. This capability enables ultra-fine perovskite patterning, making EHD jet printing a promising alternative for high-resolution optoelectronics. To enhance brightness and environmental stability in PeNC arrays, Chen et al. introduced a novel EHD-printed colloidal ink composed of PeNCs mixed with polystyrene (PS) in a nonpolar xylene solvent [281]. The addition of PS significantly improved the brightness and stability of red light-emitting PeNC arrays, addressing common issues such as low emission intensity and degradation. Unlike traditional 2D perovskite microdisk arrays, this study demonstrated the formation of 3D PeNC micropillars (Fig. 10g), which enhanced color conversion efficiency and brightness, making them highly effective as color conversion layers (CCLs) for micro-LED displays. Another advancement in EHD jet printing was made by Yang et al. [282] who developed a dual-ligand strategy to enhance the stability of PeNCs and reduce ion migration during the printing process. By incorporating lecithin and dodecanethiol (1-DT) as dual ligands, they significantly mitigated spectral shifts and emission instability caused by electric field-induced ion migration. Figure 10h compares OA/m-CsPbBrI₂ NCs, which exhibited irregular morphologies and weaker crystallinity, to dual-ligand CsPbBrI₂ NCs, which displayed well-defined square shapes, enhanced uniformity, and superior PLQY (Fig. 10i).

Despite its advantages, EHD jet printing faces several challenges when applied to PeLED fabrication. One major limitation is the high viscosity and low electric field sensitivity of typical electrode materials, which hinders the formation of stable and uniform jets, leading to inconsistent deposition. These issues make it difficult to achieve the precise electrode patterns necessary for optimal LED performance [309]. Additionally, poor adhesion between the printed patterns and electrode materials can lead to delamination or peeling during device operation, disrupting charge transport and increasing electrical resistance. This not only degrades the LED efficiency and operational lifespan but also compromises overall device stability and reliability [310]. Beyond these materials and interface constraints, the intrinsic serial, pixel-by-pixel nature of EHD jet printing limits raw throughput when scaling to large areas because each pixel must be written individually [311]. To mitigate this scalability bottleneck, multi-nozzle arrays have been introduced to boost print speed. However, close-packed nozzles can suffer electric-field crosstalk that deflects jets and produces non-uniform deposition [312, 313]. To counter crosstalk, researchers have optimized nozzle layouts and implemented auxiliary electrodes around nozzle arrays to electrostatically shield and stabilize the jets to enhance pattern fidelity and consistency [314–317]. Consequently, while EHD jet printing holds promise for high-resolution perovskite patterning, further advancements are needed to optimize material formulations and deposition techniques to enhance its applicability for PeLED manufacturing.

## Summary and Perspectives

MHPs have attracted extensive attention in both academia and industry due to their outstanding optoelectronic properties, including high color purity, narrow emission linewidth, high photoluminescence quantum efficiency, and excellent defect tolerance. As a promising candidate for next-generation display technology, PeLEDs offer significant advantages such as a wide color gamut, excellent contrast, and cost-effective fabrication. This review systematically explores perovskite film fabrication and patterning strategies for high-performance PeLEDs, classifying them into top-down and bottom-up approaches. In the top-down approach, methods such as photolithography, laser/e-beam lithography, and nanoimprinting enable precise pattern formation on perovskite films, often using PDMS molds or lithographic techniques. Conversely, the bottom-up approach focuses on precise control of perovskite crystal growth at the atomic or molecular level. Additive manufacturing strategies also provide greater flexibility in device geometry design and potential applications, enabling three-dimensional perovskite structures [318–320].

To realize their full potential in large-area, high-resolution displays, the scalable fabrication of uniform, high-quality perovskite thin films remains a foundational requirement. Although spin-coated small-area PeLEDs have demonstrated high external quantum efficiencies and operational lifetimes, large-area devices still lag behind due to challenges in achieving uniform thickness, controlled crystallization, and interfacial consistency across wide substrates [40]. Additionally, variations in film morphology and defect densities can significantly impact device reproducibility and long-term performance. To address these limitations, future research may focus on developing deposition techniques that combine scalability with fine control over film formation dynamics—such as solvent-engineered blade-coating, slot-die coating with in situ crystallization control, and hybrid vacuum-solution methods [321–323]. Moreover, integration with flexible or unconventional substrates requires careful interfacial engineering to maintain mechanical reliability and optoelectronic performance.

Building upon this, high-resolution patterning technologies are also essential for achieving full-color PeLED displays. While perovskite materials offer excellent optoelectronic properties, their inherent instability under common photolithographic and laser-based processing conditions (e.g., exposure to heat, UV light, and moisture) presents challenges in fabricating high-quality RGB microarrays with optimal optical and electrical performance [324]. Inkjet printing, particularly with multi-nozzle configurations, is a promising technique for fabricating full-color PeLEDs. However, differences in the crystallization kinetics of the 3 RGB perovskite precursors complicate uniform patterning. Thermal evaporation, a mature and scalable process, is typically restricted to single-color pixelation due to its limited compositional control. In contrast, emerging techniques such as EHD jet printing, nanoimprinting, and transfer printing offer high-resolution, non-destructive patterning strategies. EHD jet printing achieves submicron scale resolution governed by electric-field-driven droplet formation. Nanoimprinting and transfer printing, on the other hand, bypass optical and solvent-related resolution limits and are particularly advantageous for preserving film integrity. Furthermore, transfer printing enables decoupling of film crystallization and pixel alignment, offering a practical route for multicolor integration on both rigid and flexible substrates. Utilizing these approaches, it will be possible to fabricate high-precision, full-color perovskite displays with excellent optical and electrical properties.

Despite progress in efficiency optimization, the long-term operational stability of PeLEDs remains a critical bottleneck. Compared to commercial OLEDs, which exhibit high EQE (> 30%) and operation lifetimes exceeding 100,000 h, PeLEDs currently demonstrate much shorter lifetimes, often only a few hours—far below commercial viability [22, 325]. As demonstrated in perovskite photovoltaics, the instability of PeLEDs is closely linked to factors such as halide ion migration, interfacial degradation at charge transport layers, and the intrinsic chemical instability of the perovskite material [326–328]. However, PeLEDs operate under higher electric fields and harsher conditions (e.g., elevated temperatures), leading to degradation mechanisms more complex than those observed in photovoltaic devices [329]. Therefore, stability-enhancing strategies that are successful in photovoltaics may not directly translate to PeLEDs. To mitigate instability, one promising approach is the development of core/shell heterostructures, where the perovskite emitter is encapsulated within a wide-bandgap matrix, effectively passivating surface defects and suppressing trap-induced ion migration [330]. For instance, Kim et al. [26] reported BPA-capped core/shell PeNCs with a projected device half-life exceeding 30,000 h at 100 cd m^− 2^. Additionally, Guo et al. introduced dipolar molecular stabilizers to suppress field-induced phase degradation in α-FAPbI_3_ PeLEDs, achieving operational stability over 660,000 h [331]. These developments highlight the feasibility of overcoming intrinsic instability in perovskite emitters.

In addition to stability challenges, concerns over lead (Pb) toxicity have motivated ongoing efforts to develop lead-free or low-lead perovskite alternatives [332–335]. A promising strategy involves substituting Pb^2+^ with alternative B-site cations such as Sn^2+^, Ge^2+^, Cu^+^ (usually represented as Cs_3_Cu_2_X_5_, CsCu_2_X_3,_ and Cu-I cluster hybrids) or Bi^3+^ to mitigate environmental and health risks [336–342]. However, lead-free perovskites currently exhibit lower photoelectric conversion efficiency, reduced stability, and inferior processability compared to Pb-based counterparts [334, 339, 343]. Moreover, Sn^2+^, a commonly used substitute, readily oxidizes to Sn^4+^, accelerating device degradation and limiting performance [344]. To bridge this gap, future research may investigate correlations between structural, optical, and charge transport properties in lead-free perovskites, supported by theoretical predictions and compositional screening. At the same time, Pb risk may be managed on two fronts: preventing release during use and recovering Pb in a closed loop from fabrication and end-of-life. Firstly, encapsulation with Pb-sequestering layers (e.g., ionogel sorbents; mesoporous “cage-trap” adsorbents) can immobilize leaked Pb under impact/weathering—critical because unencapsulated devices can leach > 60% of their Pb within minutes of rain exposure [345]. Secondly, closed-loop recycling proven in PSCs could be ported to PeLED pilot lines: cation exchange/adsorbent capture of Pb from process solvents with conversion back to 99% purity PbI_2_; benzoic acid-assisted routes that recover > 98–99% Pb from modules or precursor mixes, and demonstrated reuse of recovered PbI_2_ in high-efficiency devices (> 20%) [346, 347]. Practically, pilot lines should add in-line Pb monitors and secondary containment, segregate Pb-bearing waste streams for adsorption/recovery, and qualify barrier stacks for Pb capture alongside moisture/oxygen ingress tests [348].

Overall, the convergence of high-quality film deposition, precise and scalable patterning, stability enhancement, and material sustainability will define the roadmap toward practical, full-color perovskite light-emitting display technologies.

## References

[1] Q.A. Akkerman, V. D’Innocenzo, S. Accornero, A. Scarpellini, A. Petrozza et al., Tuning the optical properties of cesium lead halide perovskite nanocrystals by anion exchange reactions. J. Am. Chem. Soc. **137**(32), 10276–10281 (2015). 10.1021/jacs.5b05602 26214734 10.1021/jacs.5b05602 PMC4543997

[2] Q.A. Akkerman, G. Rainò, M.V. Kovalenko, L. Manna, Genesis, challenges and opportunities for colloidal lead halide perovskite nanocrystals. Nat. Mater. **17**(5), 394–405 (2018). 10.1038/s41563-018-0018-4 29459748 10.1038/s41563-018-0018-4

[3] L. Protesescu, S. Yakunin, M.I. Bodnarchuk, F. Krieg, R. Caputo et al., Nanocrystals of cesium lead halide perovskites (CsPbX_3_, X = Cl, Br, and I): novel optoelectronic materials showing bright emission with wide color gamut. Nano Lett. **15**(6), 3692–3696 (2015). 10.1021/nl5048779 25633588 10.1021/nl5048779 PMC4462997

[4] J. Shamsi, A.S. Urban, M. Imran, L. De Trizio, L. Manna, Metal halide perovskite nanocrystals: synthesis, post-synthesis modifications, and their optical properties. Chem. Rev. **119**(5), 3296–3348 (2019). 10.1021/acs.chemrev.8b00644 30758194 10.1021/acs.chemrev.8b00644 PMC6418875

[5] M. Karlsson, Z. Yi, S. Reichert, X. Luo, W. Lin et al., Mixed halide perovskites for spectrally stable and high-efficiency blue light-emitting diodes. Nat. Commun. **12**, 361 (2021). 10.1038/s41467-020-20582-6 33441549 10.1038/s41467-020-20582-6 PMC7806600

[6] L. Zhang, L. Mei, K. Wang, Y. Lv, S. Zhang et al., Advances in the application of perovskite materials. Nano-Micro Lett. **15**, 177 (2023). 10.1007/s40820-023-01140-3 10.1007/s40820-023-01140-3 PMC10333173 37428261

[7] J. Kang, L.-W. Wang, High defect tolerance in lead halide perovskite CsPbBr_3_. J. Phys. Chem. Lett. **8**(2), 489–493 (2017). 10.1021/acs.jpclett.6b02800 28071911 10.1021/acs.jpclett.6b02800

[8] S. Kumar, J. Jagielski, N. Kallikounis, Y.-H. Kim, C. Wolf et al., Ultrapure green light-emitting diodes using two-dimensional formamidinium perovskites: achieving recommendation 2020 color coordinates. Nano Lett. **17**(9), 5277–5284 (2017). 10.1021/acs.nanolett.7b01544 28770603 10.1021/acs.nanolett.7b01544

[9] H. Huang, M.I. Bodnarchuk, S.V. Kershaw, M.V. Kovalenko, A.L. Rogach, Lead halide perovskite nanocrystals in the research spotlight: stability and defect tolerance. ACS Energy Lett. **2**(9), 2071–2083 (2017). 10.1021/acsenergylett.7b00547 28920080 10.1021/acsenergylett.7b00547 PMC5594444

[10] Y. Wu, F. Xie, H. Chen, X. Yang, H. Su et al., Thermally stable MAPbI_3_ perovskite solar cells with efficiency of 19.19% and area over 1 cm2 achieved by additive engineering. Adv. Mater. **29**(28), 1701073 (2017). 10.1002/adma.201701073 10.1002/adma.201701073 28524262

[11] M.A. Green, A. Ho-Baillie, H.J. Snaith, The emergence of perovskite solar cells. Nat. Photonics **8**(7), 506–514 (2014). 10.1038/nphoton.2014.134

[12] H. Chen, C. Liu, J. Xu, A. Maxwell, W. Zhou et al., Improved charge extraction in inverted perovskite solar cells with dual-site-binding ligands. Science **384**(6692), 189–193 (2024). 10.1126/science.adm9474 38603485 10.1126/science.adm9474

[13] S. Zhang, F. Ye, X. Wang, R. Chen, H. Zhang et al., Minimizing buried interfacial defects for efficient inverted perovskite solar cells. Science **380**(6643), 404–409 (2023). 10.1126/science.adg3755 37104579 10.1126/science.adg3755

[14] Y. Rong, Y. Hu, A. Mei, H. Tan, M.I. Saidaminov et al., Challenges for commercializing perovskite solar cells. Science **361**(6408), eaat8235 (2018). 10.1126/science.aat8235 30237326 10.1126/science.aat8235

[15] S. Liu, J. Li, W. Xiao, R. Chen, Z. Sun et al., Buried interface molecular hybrid for inverted perovskite solar cells. Nature **632**(8025), 536–542 (2024). 10.1038/s41586-024-07723-3 38925147 10.1038/s41586-024-07723-3

[16] M.A. Green, E.D. Dunlop, M. Yoshita, N. Kopidakis, K. Bothe et al., Solar cell efficiency tables (version 66). Prog. Photovoltaics Res. Appl. **33**(7), 795–810 (2025). 10.1002/pip.3919

[17] J. Liu, Y. He, L. Ding, H. Zhang, Q. Li et al., Perovskite/silicon tandem solar cells with bilayer interface passivation. Nature **635**(8039), 596–603 (2024). 10.1038/s41586-024-07997-7 39236747 10.1038/s41586-024-07997-7

[18] Z. Liu, R. Lin, M. Wei, M. Yin, P. Wu et al., All-perovskite tandem solar cells achieving >29% efficiency with improved (100) orientation in wide-bandgap perovskites. Nat. Mater. **24**(2), 252–259 (2025). 10.1038/s41563-024-02073-x 39794635 10.1038/s41563-024-02073-x

[19] H. Wang, X. Gong, D. Zhao, Y.-B. Zhao, S. Wang et al., A multi-functional molecular modifier enabling efficient large-area perovskite light-emitting diodes. Joule **4**(9), 1977–1987 (2020). 10.1016/j.joule.2020.07.002

[20] M.T. Hoang, A.S. Pannu, Y. Yang, S. Madani, P. Shaw et al., Surface treatment of inorganic CsPbI_3_ nanocrystals with guanidinium iodide for efficient perovskite light-emitting diodes with high brightness. Nano-Micro Lett. **14**(1), 69 (2022). 10.1007/s40820-022-00813-9 10.1007/s40820-022-00813-9 PMC8891416 35237871

[21] G.H. Lee, K. Kim, Y. Kim, J. Yang, M.K. Choi, Recent advances in patterning strategies for full-color perovskite light-emitting diodes. Nano-Micro Lett. **16**(1), 45 (2023). 10.1007/s40820-023-01254-8 10.1007/s40820-023-01254-8 PMC10704014 38060071

[22] C. Sun, Y. Jiang, K. Wei, M. Yuan, Perovskite light-emitting diodes toward commercial full-colour displays: progress and key technical obstacles. Light: Adv. Manuf. **4**(3), 1 (2023). 10.37188/lam.2023.015

[23] B.R. Sutherland, E.H. Sargent, Perovskite photonic sources. Nat. Photonics **10**(5), 295–302 (2016). 10.1038/nphoton.2016.62

[24] F. Palazon, F. Di Stasio, Q.A. Akkerman, R. Krahne, M. Prato et al., Polymer-free films of inorganic halide perovskite nanocrystals as UV-to-white color-conversion layers in LEDs. Chem. Mater. **28**(9), 2902–2906 (2016). 10.1021/acs.chemmater.6b00954 27563171 10.1021/acs.chemmater.6b00954 PMC4993521

[25] Z.-K. Tan, R.S. Moghaddam, M.L. Lai, P. Docampo, R. Higler et al., Bright light-emitting diodes based on organometal halide perovskite. Nat. Nanotechnol. **9**(9), 687–692 (2014). 10.1038/nnano.2014.149 25086602 10.1038/nnano.2014.149

[26] J.S. Kim, J.-M. Heo, G.-S. Park, S.-J. Woo, C. Cho et al., Ultra-bright, efficient and stable perovskite light-emitting diodes. Nature **611**(7937), 688–694 (2022). 10.1038/s41586-022-05304-w 36352223 10.1038/s41586-022-05304-w

[27] Y. Nong, J. Yao, J. Li, L. Xu, Z. Yang et al., Boosting external quantum efficiency of blue perovskite QLEDs exceeding 23% by trifluoroacetate passivation and mixed hole transportation design. Adv. Mater. **36**(27), 2402325 (2024). 10.1002/adma.202402325 10.1002/adma.202402325 38631673

[28] L. Kong, Y. Sun, B. Zhao, K. Ji, J. Feng et al., Fabrication of red-emitting perovskite LEDs by stabilizing their octahedral structure. Nature **631**(8019), 73–79 (2024). 10.1038/s41586-024-07531-9 38867044 10.1038/s41586-024-07531-9

[29] Z. Liu, W. Qiu, X. Peng, G. Sun, X. Liu et al., Perovskite light-emitting diodes with EQE exceeding 28% through a synergetic dual-additive strategy for defect passivation and nanostructure regulation. Adv. Mater. **33**(43), 2103268 (2021). 10.1002/adma.202103268 10.1002/adma.202103268 34545631

[30] Y. Gao, H. Li, X. Dai, X. Ying, Z. Liu et al., Microsecond-response perovskite light-emitting diodes for active-matrix displays. Nat. Electron. **7**(6), 487–496 (2024). 10.1038/s41928-024-01181-5

[31] S. Yuan, L. Dai, Y. Sun, F. Auras, Y.-H. Zhou et al., Efficient blue electroluminescence from reduced-dimensional perovskites. Nat. Photon. **18**(5), 425–431 (2024). 10.1038/s41566-024-01382-6

[32] G.-H. Lee, H. Moon, H. Kim, G.H. Lee, W. Kwon et al., Multifunctional materials for implantable and wearable photonic healthcare devices. Nat. Rev. Mater. **5**(2), 149–165 (2020). 10.1038/s41578-019-0167-3 32728478 10.1038/s41578-019-0167-3 PMC7388681

[33] S. Choi, H. Lee, R. Ghaffari, T. Hyeon, D.-H. Kim, Recent advances in flexible and stretchable bio-electronic devices integrated with nanomaterials. Adv. Mater. **28**(22), 4203–4218 (2016). 10.1002/adma.201504150 26779680 10.1002/adma.201504150

[34] J.I. Kwon, G. Park, G.H. Lee, J.H. Jang, N.J. Sung et al., Ultrahigh-resolution full-color perovskite nanocrystal patterning for ultrathin skin-attachable displays. Sci. Adv. **8**(43), eadd0697 (2022). 10.1126/sciadv.add0697 36288304 10.1126/sciadv.add0697 PMC9604611

[35] S.G.R. Bade, X. Shan, P.T. Hoang, J. Li, T. Geske et al., Stretchable light-emitting diodes with organometal-halide-perovskite–polymer composite emitters. Adv. Mater. **29**(23), 1607053 (2017). 10.1002/adma.201607053 10.1002/adma.201607053 28387463

[36] Y. Shen, M.-N. Li, Y. Li, F.-M. Xie, H.-Y. Wu et al., Rational interface engineering for efficient flexible perovskite light-emitting diodes. ACS Nano **14**(5), 6107–6116 (2020). 10.1021/acsnano.0c01908 32223190 10.1021/acsnano.0c01908

[37] F. Chun, B. Zhang, Y. Gao, X. Wei, Q. Zhang et al., Multicolour stretchable perovskite electroluminescent devices for user-interactive displays. Nat. Photon. **18**(8), 856–863 (2024). 10.1038/s41566-024-01455-6

[38] Y.H. Song, J. Ge, L.B. Mao, K.H. Wang, X.L. Tai et al., Planar defect-free pure red perovskite light-emitting diodes *via* metastable phase crystallization. Sci. Adv. **8**(45), eabq2321 (2022). 10.1126/sciadv.abq2321 36367940 10.1126/sciadv.abq2321 PMC9651863

[39] C. Sun, Y. Jiang, M. Cui, L. Qiao, J. Wei et al., High-performance large-area quasi-2D perovskite light-emitting diodes. Nat. Commun. **12**, 2207 (2021). 10.1038/s41467-021-22529-x 33850141 10.1038/s41467-021-22529-x PMC8044177

[40] J. Luo, J. Li, L. Grater, R. Guo, A.R. bin Mohd Yusoff et al., Vapour-deposited perovskite light-emitting diodes. Nat. Rev. Mater. **9**(4), 282–294 (2024). 10.1038/s41578-024-00651-8

[41] Y. Zou, P. Teng, W. Xu, G. Zheng, W. Lin et al., Manipulating crystallization dynamics through chelating molecules for bright perovskite emitters. Nat. Commun. **12**, 4831 (2021). 10.1038/s41467-021-25092-7 34376647 10.1038/s41467-021-25092-7 PMC8355273

[42] J.-W. Lee, S.M. Kang, Patterning of metal halide perovskite thin films and functional layers for optoelectronic applications. Nano-Micro Lett. **15**(1), 184 (2023). 10.1007/s40820-023-01154-x 10.1007/s40820-023-01154-x PMC10354233 37462884

[43] X. Yang, L. Ma, L. Li, M. Luo, X. Wang et al., Towards micro-PeLED displays. Nat. Rev. Mater. **8**(5), 341–353 (2023). 10.1038/s41578-022-00522-0

[44] T.-H. Han, K.Y. Jang, Y. Dong, R.H. Friend, E.H. Sargent et al., A roadmap for the commercialization of perovskite light emitters. Nat. Rev. Mater. **7**(10), 757–777 (2022). 10.1038/s41578-022-00459-4

[45] Y. Dong, Y.-K. Wang, F. Yuan, A. Johnston, Y. Liu et al., Bipolar-shell resurfacing for blue LEDs based on strongly confined perovskite quantum dots. Nat. Nanotechnol. **15**(8), 668–674 (2020). 10.1038/s41565-020-0714-5 32632321 10.1038/s41565-020-0714-5

[46] L. Zhang, C. Sun, T. He, Y. Jiang, J. Wei et al., High-performance quasi-2D perovskite light-emitting diodes: from materials to devices. Light. Sci. Appl. **10**, 61 (2021). 10.1038/s41377-021-00501-0 33741895 10.1038/s41377-021-00501-0 PMC7979804

[47] X. Zhao, Z.-K. Tan, Large-area near-infrared perovskite light-emitting diodes. Nat. Photonics **14**(4), 215–218 (2020). 10.1038/s41566-019-0559-3

[48] C. Chen, T.-H. Han, S. Tan, J. Xue, Y. Zhao et al., Efficient flexible inorganic perovskite light-emitting diodes fabricated with CsPbBr_3_ emitters prepared* vi*a low-temperature* in sit*u dynamic thermal crystallization. Nano Lett. **20**(6), 4673–4680 (2020). 10.1021/acs.nanolett.0c01550 32437162 10.1021/acs.nanolett.0c01550

[49] Y. Shen, J.-K. Wang, Y.-Q. Li, K.-C. Shen, Z.-H. Su et al., Interfacial “anchoring effect” enables efficient large-area sky-blue perovskite light-emitting diodes. Adv. Sci. **8**(19), 2102213 (2021). 10.1002/advs.202102213 10.1002/advs.202102213 PMC8498857 34453782

[50] Y.-H. Kim, S. Kim, A. Kakekhani, J. Park, J. Park et al., Comprehensive defect suppression in perovskite nanocrystals for high-efficiency light-emitting diodes. Nat. Photonics **15**(2), 148–155 (2021). 10.1038/s41566-020-00732-4

[51] S. Chu, W. Chen, Z. Fang, X. Xiao, Y. Liu et al., Large-area and efficient perovskite light-emitting diodes *via* low-temperature blade-coating. Nat. Commun. **12**, 147 (2021). 10.1038/s41467-020-20433-4 33420040 10.1038/s41467-020-20433-4 PMC7794572

[52] C. Chen, L. Zeng, Z. Jiang, Z. Xu, Y. Chen et al., Vacuum-assisted preparation of high-quality quasi-2D perovskite thin films for large-area light-emitting diodes. Adv. Funct. Mater. **32**(4), 2107644 (2022). 10.1002/adfm.202107644

[53] P. Du, J. Li, L. Wang, L. Sun, X. Wang et al., Efficient and large-area all vacuum-deposited perovskite light-emitting diodes *via* spatial confinement. Nat. Commun. **12**, 4751 (2021). 10.1038/s41467-021-25093-6 34362915 10.1038/s41467-021-25093-6 PMC8346511

[54] S. Chu, Y. Zhang, P. Xiao, W. Chen, R. Tang et al., Large-area and efficient sky-blue perovskite light-emitting diodes *via* blade-coating. Adv. Mater. **34**(16), 2108939 (2022). 10.1002/adma.202108939 10.1002/adma.202108939 35181956

[55] Y.-H. Kim, J. Park, S. Kim, J.S. Kim, H. Xu et al., Exploiting the full advantages of colloidal perovskite nanocrystals for large-area efficient light-emitting diodes. Nat. Nanotechnol. **17**(6), 590–597 (2022). 10.1038/s41565-022-01113-4 35577974 10.1038/s41565-022-01113-4

[56] D. Zhang, Q. Zhang, B. Ren, Y. Zhu, M. Abdellah et al., Large-scale planar and spherical light-emitting diodes based on arrays of perovskite quantum wires. Nat. Photonics **16**(4), 284–290 (2022). 10.1038/s41566-022-00978-0

[57] Y.B. Cao, D. Zhang, Q. Zhang, X. Qiu, Y. Zhou et al., High-efficiency, flexible and large-area red/green/blue all-inorganic metal halide perovskite quantum wires-based light-emitting diodes. Nat. Commun. **14**, 4611 (2023). 10.1038/s41467-023-40150-y 37528109 10.1038/s41467-023-40150-y PMC10393990

[58] L. Kong, C. Sun, M. You, Y. Jiang, G. Wang et al., Universal molecular control strategy for scalable fabrication of perovskite light-emitting diodes. Nano Lett. **23**(3), 985–992 (2023). 10.1021/acs.nanolett.2c04459 36715576 10.1021/acs.nanolett.2c04459

[59] J. Li, P. Du, Q. Guo, L. Sun, Z. Shen et al., Efficient all-thermally evaporated perovskite light-emitting diodes for active-matrix displays. Nat. Photon. **17**(5), 435–441 (2023). 10.1038/s41566-023-01177-1

[60] G. Shi, Z. Huang, R. Qiao, W. Chen, Z. Li et al., Manipulating solvent fluidic dynamics for large-area perovskite film-formation and white light-emitting diodes. Nat. Commun. **15**(1), 1066 (2024). 10.1038/s41467-024-45488-5 38316825 10.1038/s41467-024-45488-5 PMC10844237

[61] C.-H. Tien, J.-Q. Liu, L.-C. Chen, Post-hot-cast annealing deposition of perovskite films with infused multifunctional organic molecules to enhance the performance of large-area light-emitting devices. RSC Adv. **14**(26), 18567–18575 (2024). 10.1039/d4ra02652g 38860259 10.1039/d4ra02652g PMC11163951

[62] K. Wei, T. Zhou, Y. Jiang, C. Sun, Y. Liu et al., Perovskite heteroepitaxy for high-efficiency and stable pure-red LEDs. Nature **638**(8052), 949–956 (2025). 10.1038/s41586-024-08503-9 39972133 10.1038/s41586-024-08503-9

[63] G. Chen, S. Wang, Z. Yu, C. Dong, P. Jia et al., Regulation of nucleation and crystallization for blade-coating large-area CsPbBr_3_ perovskite light-emitting diodes. Sci. Bull. **70**(2), 212–222 (2025). 10.1016/j.scib.2024.10.022 10.1016/j.scib.2024.10.022 39477787

[64] W.-Z. Liu, Y. Wang, J.-Z. Xu, S.-H. Xu, D.-Y. Zhou et al., Enhancing carrier balance in blade-coated near-infrared quantum dot light-emitting diodes by a PSS-rich PEDOT: PSS hole-buffering layer. Small **21**(44), e04662 (2025). 10.1002/smll.202504662 40937678 10.1002/smll.202504662

[65] Y. Li, N. Meng, Y. Xu, B. Yu, J. Liu et al., Sequential layer-by-layer deposition for high-performance fully thermal-evaporated red perovskite light-emitting diodes. Nat. Commun. **16**, 6908 (2025). 10.1038/s41467-025-62282-z 40715111 10.1038/s41467-025-62282-z PMC12297250

[66] S. Ding, Q. Wang, W. Gu, Z. Tang, B. Zhang et al., Phase dimensions resolving of efficient and stable perovskite light-emitting diodes at high brightness. Nat. Photon. **18**(4), 363–370 (2024). 10.1038/s41566-023-01372-0

[67] S. Zheng, Z. Wang, N. Jiang, H. Huang, X. Wu et al., Ultralow voltage-driven efficient and stable perovskite light-emitting diodes. Sci. Adv. **10**(36), eadp8473 (2024). 10.1126/sciadv.adp8473 39241067 10.1126/sciadv.adp8473 PMC11378915

[68] H. Li, Y. Feng, M. Zhu, Y. Gao, C. Fan et al., Nanosurface-reconstructed perovskite for highly efficient and stable active-matrix light-emitting diode display. Nat. Nanotechnol. **19**(5), 638–645 (2024). 10.1038/s41565-024-01652-y 38649747 10.1038/s41565-024-01652-y

[69] N.-G. Park, K. Zhu, Scalable fabrication and coating methods for perovskite solar cells and solar modules. Nat. Rev. Mater. **5**(5), 333–350 (2020). 10.1038/s41578-019-0176-2

[70] H. Liu, G. Shi, C. Peng, W. Chen, H. Yao et al., Advances and challenges in large-area perovskite light-emitting diodes. Adv. Mater. **37**(25), 2410154 (2025). 10.1002/adma.202410154 10.1002/adma.202410154 39318091

[71] Z. Ma, Z. Shi, C. Qin, M. Cui, D. Yang et al., Stable yellow light-emitting devices based on ternary copper halides with broadband emissive self-trapped excitons. ACS Nano **14**(4), 4475–4486 (2020). 10.1021/acsnano.9b10148 32167288 10.1021/acsnano.9b10148

[72] Y. Zou, Y. Li, X. Pang, Y. Song, W. Xu et al., Unraveling deposition atmosphere impact on reproducibility of perovskite light-emitting diodes. J. Phys. Chem. Lett. **14**(21), 5025–5032 (2023). 10.1021/acs.jpclett.3c01067 37227043 10.1021/acs.jpclett.3c01067

[73] Y. Han, J. Wang, C.G. Bischak, S. Kim, K. Lee et al., Significance of ambient temperature control for highly reproducible layered perovskite light-emitting diodes. ACS Photonics **7**(9), 2489–2497 (2020). 10.1021/acsphotonics.0c00779

[74] Y. Zhao, C. Liu, M. Li, X. Chen, Z. Song et al., Custom-tailored surface morphology for efficient quasi-2D perovskite light-emitting diodes. Adv. Funct. Mater. **35**(37), 2503978 (2025). 10.1002/adfm.202503978

[75] S. Chi, Y. Chen, X. Li, J. Wang, Z. Zhao et al., Functional molecule surface infiltration treatment for efficient all-inorganic perovskite light-emitting diodes. Chem. Eng. J. **514**, 162787 (2025). 10.1016/j.cej.2025.162787

[76] S. Wang, X. Sun, J. Shi, R. Zhao, B. Zhang et al., Additive-driven enhancement of crystallization: strategies and prospects for boosting photoluminescence quantum yields in halide perovskite films for light-emitting diodes. Adv. Mater. **36**(52), e2413673 (2024). 10.1002/adma.202413673 39506414 10.1002/adma.202413673

[77] M. Hu, S. Fernández, Q. Zhou, P. Narayanan, B. Saini et al., Water additives improve the efficiency of violet perovskite light-emitting diodes. Matter **6**(7), 2356–2367 (2023). 10.1016/j.matt.2023.05.018

[78] L. Zhu, H. Cao, C. Xue, H. Zhang, M. Qin et al., Unveiling the additive-assisted oriented growth of perovskite crystallite for high performance light-emitting diodes. Nat. Commun. **12**(1), 5081 (2021). 10.1038/s41467-021-25407-8 34426580 10.1038/s41467-021-25407-8 PMC8382739

[79] M. Li, Y. Zhao, J. Guo, X. Qin, Q. Zhang et al., Phase regulation and defect passivation enabled by phosphoryl chloride molecules for efficient quasi-2D perovskite light-emitting diodes. Nano-Micro Lett. **15**(1), 119 (2023). 10.1007/s40820-023-01089-3 10.1007/s40820-023-01089-3 PMC10151432 37127730

[80] M. Li, Y. Yang, Z. Kuang, C. Hao, S. Wang et al., Acceleration of radiative recombination for efficient perovskite LEDs. Nature **630**(8017), 631–635 (2024). 10.1038/s41586-024-07460-7 38811739 10.1038/s41586-024-07460-7 PMC11186751

[81] J. Li, C. Duan, Q. Zhang, C. Chen, Q. Wen et al., Self-generated buried submicrocavities for high-performance near-infrared perovskite light-emitting diode. Nano-Micro Lett. **15**(1), 125 (2023). 10.1007/s40820-023-01097-3 10.1007/s40820-023-01097-3 PMC10185725 37188867

[82] Y.-K. Wang, F. Jia, X. Li, S. Teale, P. Xia et al., Self-assembled monolayer–based blue perovskite LEDs. Sci. Adv. **9**(36), eadh2140 (2023). 10.1126/sciadv.adh2140 37683007 10.1126/sciadv.adh2140 PMC10491221

[83] N. Wang, L. Cheng, R. Ge, S. Zhang, Y. Miao et al., Perovskite light-emitting diodes based on solution-processed self-organized multiple quantum wells. Nat. Photonics **10**(11), 699–704 (2016). 10.1038/nphoton.2016.185

[84] Y. Cao, N. Wang, H. Tian, J. Guo, Y. Wei et al., Perovskite light-emitting diodes based on spontaneously formed submicrometre-scale structures. Nature **562**(7726), 249–253 (2018). 10.1038/s41586-018-0576-2 30305742 10.1038/s41586-018-0576-2

[85] S. Yuan, Z.-K. Wang, M.-P. Zhuo, Q.-S. Tian, Y. Jin et al., Self-assembled high quality CsPbBr_3_ quantum dot films toward highly efficient light-emitting diodes. ACS Nano **12**(9), 9541–9548 (2018). 10.1021/acsnano.8b05185 30199226 10.1021/acsnano.8b05185

[86] L. Ni, U. Huynh, A. Cheminal, T.H. Thomas, R. Shivanna et al., Real-time observation of exciton–phonon coupling dynamics in self-assembled hybrid perovskite quantum wells. ACS Nano **11**(11), 10834–10843 (2017). 10.1021/acsnano.7b03984 29064668 10.1021/acsnano.7b03984

[87] B. Yu, C. Zhang, L. Chen, X. Huang, Z. Qin et al., Exciton linewidth broadening induced by exciton–phonon interactions in CsPbBr_3_ nanocrystals. J. Chem. Phys. **154**(21), 214502 (2021). 10.1063/5.0051611 34240983 10.1063/5.0051611

[88] Y. Jiang, C. Qin, M. Cui, T. He, K. Liu et al., Spectra stable blue perovskite light-emitting diodes. Nat. Commun. **10**, 1868 (2019). 10.1038/s41467-019-09794-7 31015430 10.1038/s41467-019-09794-7 PMC6478869

[89] J. Jiang, M. Shi, Z. Xia, Y. Cheng, Z. Chu et al., Efficient pure-red perovskite light-emitting diodes with strong passivation *via* ultrasmall-sized molecules. Sci. Adv. **10**(18), eadn5683 (2024). 10.1126/sciadv.adn5683 38701203 10.1126/sciadv.adn5683 PMC11067999

[90] F. Yuan, G. Folpini, T. Liu, U. Singh, A. Treglia et al., Bright and stable near-infrared lead-free perovskite light-emitting diodes. Nat. Photon. **18**(2), 170–176 (2024). 10.1038/s41566-023-01351-5

[91] Z. Xia, J. Jiang, A. Wang, D. An, Z. Li et al., Overall performance improvement of perovskite green LEDs by CsPbBr_3_&Cs_4_PbBr_6_ nanocrystals and molecular doping. Adv. Mater. **37**(34), 2506187 (2025). 10.1002/adma.202506187 10.1002/adma.202506187 40489126

[92] A. Swarnkar, A.R. Marshall, E.M. Sanehira, B.D. Chernomordik, D.T. Moore et al., Quantum dot–induced phase stabilization of α-CsPbI_3_perovskite for high-efficiency photovoltaics. Science **354**(6308), 92–95 (2016). 10.1126/science.aag2700 27846497 10.1126/science.aag2700

[93] A.D. Wright, C. Verdi, R.L. Milot, G.E. Eperon, M.A. Pérez-Osorio et al., Electron–phonon coupling in hybrid lead halide perovskites. Nat. Commun. **7**, 11755 (2016). 10.1038/ncomms11755 10.1038/ncomms11755 PMC4894981 27225329

[94] S.-D. Baek, W. Shao, W. Feng, Y. Tang, Y.H. Lee et al., Grain engineering for efficient near-infrared perovskite light-emitting diodes. Nat. Commun. **15**, 10760 (2024). 10.1038/s41467-024-55075-3 39737972 10.1038/s41467-024-55075-3 PMC11685452

[95] H. Cho, S.-H. Jeong, M.-H. Park, Y.-H. Kim, C. Wolf et al., Overcoming the electroluminescence efficiency limitations of perovskite light-emitting diodes. Science **350**(6265), 1222–1225 (2015). 10.1126/science.aad1818 26785482 10.1126/science.aad1818

[96] K. Lin, J. Xing, L.N. Quan, F.P.G. de Arquer, X. Gong et al., Perovskite light-emitting diodes with external quantum efficiency exceeding 20 per cent. Nature **562**(7726), 245–248 (2018). 10.1038/s41586-018-0575-3 30305741 10.1038/s41586-018-0575-3

[97] Z. Chu, W. Zhang, J. Jiang, Z. Qu, F. Ma et al., Blue light-emitting diodes based on quasi-two-dimensional perovskite with efficient charge injection and optimized phase distribution *via* an alkali metal salt. Nat. Electron. **6**(5), 360–369 (2023). 10.1038/s41928-023-00955-7

[98] Y.-H. Kim, C. Wolf, Y.-T. Kim, H. Cho, W. Kwon et al., Highly efficient light-emitting diodes of colloidal metal–halide perovskite nanocrystals beyond quantum size. ACS Nano **11**(7), 6586–6593 (2017). 10.1021/acsnano.6b07617 28587467 10.1021/acsnano.6b07617

[99] X. Li, Y. Wu, S. Zhang, B. Cai, Y. Gu et al., CsPbX3 quantum dots for lighting and displays: room-temperature synthesis, photoluminescence superiorities, underlying origins and white light-emitting diodes. Adv. Funct. Mater. **26**(15), 2435–2445 (2016). 10.1002/adfm.201600109

[100] Z. Xiao, R.A. Kerner, L. Zhao, N.L. Tran, K.M. Lee et al., Efficient perovskite light-emitting diodes featuring nanometre-sized crystallites. Nat. Photon. **11**(2), 108–115 (2017). 10.1038/nphoton.2016.269

[101] H. Wang, X. Zhang, Q. Wu, F. Cao, D. Yang et al., Trifluoroacetate induced small-grained CsPbBr_3_ perovskite films result in efficient and stable light-emitting devices. Nat. Commun. **10**(1), 665 (2019). 10.1038/s41467-019-08425-5 30737389 10.1038/s41467-019-08425-5 PMC6368619

[102] Y. Hassan, J.H. Park, M.L. Crawford, A. Sadhanala, J. Lee et al., Ligand-engineered bandgap stability in mixed-halide perovskite LEDs. Nature **591**(7848), 72–77 (2021). 10.1038/s41586-021-03217-8 33658694 10.1038/s41586-021-03217-8

[103] J. Zhang, T. Zhang, Z. Ma, F. Yuan, X. Zhou et al., A multifunctional “halide-equivalent” anion enabling efficient CsPb(Br/I)_3_ nanocrystals pure-red light-emitting diodes with external quantum efficiency exceeding 23%. Adv. Mater. **35**(8), 2209002 (2023). 10.1002/adma.202209002 10.1002/adma.202209002 36493461

[104] J. Zhang, B. Cai, X. Zhou, F. Yuan, C. Yin et al., Ligand-induced cation-π interactions enable high-efficiency, bright, and spectrally stable rec. 2020 pure-red perovskite light-emitting diodes. Adv. Mater. **35**(45), e2303938 (2023). 10.1002/adma.202303938 37464982 10.1002/adma.202303938

[105] X. Fu, M. Wang, Y. Jiang, X. Guo, X. Zhao et al., Mixed-halide perovskites with halogen bond induced interlayer locking structure for stable pure-red PeLEDs. Nano Lett. **23**(14), 6465–6473 (2023). 10.1021/acs.nanolett.3c01319 37413789 10.1021/acs.nanolett.3c01319

[106] Y. Liu, Y. Dong, T. Zhu, D. Ma, A. Proppe et al., Bright and stable light-emitting diodes based on perovskite quantum dots in perovskite matrix. J. Am. Chem. Soc. **143**(38), 15606–15615 (2021). 10.1021/jacs.1c02148 34542273 10.1021/jacs.1c02148

[107] X. Yang, L. Ma, M. Yu, H.-H. Chen, Y. Ji et al., Focus on perovskite emitters in blue light-emitting diodes. Light Sci. Appl. **12**(1), 177 (2023). 10.1038/s41377-023-01206-2 37482582 10.1038/s41377-023-01206-2 PMC10363551

[108] J. Zhang, L. Wang, X. Zhang, G. Xie, G. Jia et al., Blue light-emitting diodes based on halide perovskites: recent advances and strategies. Mater. Today **51**, 222–246 (2021). 10.1016/j.mattod.2021.10.023

[109] M. Aftabuzzaman, Y. Hong, S. Jeong, R. Levan, S.J. Lee et al., Colloidal perovskite nanocrystals for blue-light-emitting diodes and displays. Adv. Sci. **12**(15), 2409736 (2025). 10.1002/advs.202409736 10.1002/advs.202409736 PMC12005814 40059086

[110] Y. Deng, C.H. Van Brackle, X. Dai, J. Zhao, B. Chen et al., Tailoring solvent coordination for high-speed, room-temperature blading of perovskite photovoltaic films. Sci. Adv. **5**(12), eaax7537 (2019). 10.1126/sciadv.aax7537 31840067 10.1126/sciadv.aax7537 PMC6897546

[111] J. Yang, E.L. Lim, L. Tan, Z. Wei, Ink engineering in blade-coating large-area perovskite solar cells. Adv. Energy Mater. **12**(28), 2200975 (2022). 10.1002/aenm.202200975

[112] Z. Yang, C.-C. Chueh, F. Zuo, J.H. Kim, P.-W. Liang et al., High-performance fully printable perovskite solar cells *via* blade-coating technique under the ambient condition. Adv. Energy Mater. **5**(13), 1500328 (2015). 10.1002/aenm.201500328

[113] J. Li, R. Munir, Y. Fan, T. Niu, Y. Liu et al., Phase transition control for high-performance blade-coated perovskite solar cells. Joule **2**(7), 1313–1330 (2018). 10.1016/j.joule.2018.04.011

[114] Y. Xiao, C. Zuo, J.-X. Zhong, W.-Q. Wu, L. Shen et al., Large-area blade-coated solar cells: advances and perspectives. Adv. Energy Mater. **11**(21), 2100378 (2021). 10.1002/aenm.202100378

[115] W. Zhao, S. Zhang, Y. Zhang, S. Li, X. Liu et al., Environmentally friendly solvent-processed organic solar cells that are highly efficient and adaptable for the blade-coating method. Adv. Mater. **30**(4), 1704837 (2018). 10.1002/adma.201704837 10.1002/adma.201704837 29219217

[116] S.G.R. Bade, J. Li, X. Shan, Y. Ling, Y. Tian et al., Fully printed halide perovskite light-emitting diodes with silver nanowire electrodes. ACS Nano **10**(2), 1795–1801 (2016). 10.1021/acsnano.5b07506 26713348 10.1021/acsnano.5b07506

[117] Y. Liu, J. Cui, K. Du, H. Tian, Z. He et al., Efficient blue light-emitting diodes based on quantum-confined bromide perovskite nanostructures. Nat. Photonics **13**(11), 760–764 (2019). 10.1038/s41566-019-0505-4

[118] Z. Chen, Z. Li, C. Zhang, X.-F. Jiang, D. Chen et al., Recombination dynamics study on nanostructured perovskite light-emitting devices. Adv. Mater. **30**(38), 1801370 (2018). 10.1002/adma.201801370 10.1002/adma.201801370 30088297

[119] Q. Hu, L. Zhao, J. Wu, K. Gao, D. Luo et al., *In situ* dynamic observations of perovskite crystallisation and microstructure evolution intermediated from [PbI_6_]^4-^ cage nanoparticles. Nat. Commun. **8**, 15688 (2017). 10.1038/ncomms15688 28635947 10.1038/ncomms15688 PMC5482054

[120] Y.-H. Kim, Y. Zhai, E.A. Gaulding, S.N. Habisreutinger, T. Moot et al., Strategies to achieve high circularly polarized luminescence from colloidal organic–inorganic hybrid perovskite nanocrystals. ACS Nano **14**(7), 8816–8825 (2020). 10.1021/acsnano.0c03418 32644773 10.1021/acsnano.0c03418 PMC10906077

[121] J. Li, P. Du, S. Li, J. Liu, M. Zhu et al., High-throughput combinatorial optimizations of perovskite light-emitting diodes based on all-vacuum deposition. Adv. Funct. Mater. **29**(51), 1903607 (2019). 10.1002/adfm.201903607

[122] J. Li, L. Yang, Q. Guo, P. Du, L. Wang et al., All-vacuum fabrication of yellow perovskite light-emitting diodes. Sci. Bull. **67**(2), 178–185 (2022). 10.1016/j.scib.2021.09.003 10.1016/j.scib.2021.09.003 36546011

[123] S. Ji, S.-R. Bae, L. Hu, A.T. Hoang, M.J. Seol et al., Perovskite light-emitting diode display based on MoS_2_ backplane thin-film transistors. Adv. Mater. **36**(2), e2309531 (2024). 10.1002/adma.202309531 37985162 10.1002/adma.202309531

[124] J. Ávila, C. Momblona, P.P. Boix, M. Sessolo, H.J. Bolink, Vapor-deposited perovskites: the route to high-performance solar cell production? Joule **1**(3), 431–442 (2017). 10.1016/j.joule.2017.07.014

[125] R. Ilmi, D. Zhang, J.D.L. Dutra, N. Dege, L. Zhou et al., A tris β-diketonate europium(III) complex based OLED fabricated by thermal evaporation method displaying efficient bright red emission. Org. Electron. **96**, 106216 (2021). 10.1016/j.orgel.2021.106216

[126] C.B. Lee, A. Uddin, X. Hu, Anderssonb, Study of Alq3 thermal evaporation rate effects on the OLED. Mater. Sci. Eng. B **112**(1), 14–18 (2004). 10.1016/j.mseb.2004.05.009

[127] F.U. Kosasih, E. Erdenebileg, N. Mathews, S.G. Mhaisalkar, A. Bruno, Thermal evaporation and hybrid deposition of perovskite solar cells and mini-modules. Joule **6**(12), 2692–2734 (2022). 10.1016/j.joule.2022.11.004

[128] S.R. Bae, D.Y. Heo, S.Y. Kim, Recent progress of perovskite devices fabricated using thermal evaporation method: perspective and outlook. Mater. Today Adv. **14**, 100232 (2022). 10.1016/j.mtadv.2022.100232

[129] F. Mariano, A. Listorti, A. Rizzo, S. Colella, G. Gigli et al., Thermally evaporated hybrid perovskite for hetero-structured green light-emitting diodes. Appl. Phys. Lett. **111**(16), 163301 (2017). 10.1063/1.5001828

[130] J. Zhu, J. Li, Y. Huang, N. Liu, L. Sun et al., All-thermally evaporated blue perovskite light-emitting diodes for active matrix displays. Small Methods **8**(1), 2300712 (2024). 10.1002/smtd.202300712 10.1002/smtd.202300712 37821420

[131] I.J. Cleveland, M.N. Tran, A. Dey, E.S. Aydil, Vapor deposition of CsPbBr_3_ thin films by evaporation of CsBr and PbBr_2_. J. Vac. Sci. Technol. A Vac. Surf. Films **39**(4), 043415 (2021). 10.1116/6.0000875

[132] E.J. Juarez-Perez, L.K. Ono, Y. Qi, Thermal degradation of formamidinium based lead halide perovskites into sym-triazine and hydrogen cyanide observed by coupled thermogravimetry-mass spectrometry analysis. J. Mater. Chem. A **7**(28), 16912–16919 (2019). 10.1039/C9TA06058H

[133] S. He, L. Qin, Z. Liu, J.-W. Kang, J. Luo et al., Efficient thermally evaporated near-infrared perovskite light-emitting diodes *via* phase regulation. Nano-Micro Lett. **17**(1), 270 (2025). 10.1007/s40820-025-01776-3 10.1007/s40820-025-01776-3 PMC12098251 40402173

[134] S. Sanders, G. Simkus, J. Riedel, A. Ost, A. Schmitz et al., Showerhead-assisted chemical vapor deposition of CsPbBr_3_ films for LED applications. J. Mater. Res. **36**(9), 1813–1823 (2021). 10.1557/s43578-021-00239-w

[135] L. Qiu, S. He, L.K. Ono, Y. Qi, Progress of surface science studies on ABX3-based metal halide perovskite solar cells. Adv. Energy Mater. **10**(13), 1902726 (2020). 10.1002/aenm.201902726

[136] M. Shin, H.S. Lee, Y.C. Sim, Y.-H. Cho, K.C. Choi et al., Modulation of growth kinetics of vacuum-deposited CsPbBr_3_ films for efficient light-emitting diodes. ACS Appl. Mater. Interfaces **12**(1), 1944–1952 (2020). 10.1021/acsami.9b20094 31815412 10.1021/acsami.9b20094

[137] Q. Guesnay, F. Sahli, C. Ballif, Q. Jeangros, Vapor deposition of metal halide perovskite thin films: process control strategies to shape layer properties. APL Mater. **9**(10), 100703 (2021). 10.1063/5.0060642

[138] Y. Fu, Q. Zhang, D. Zhang, Y. Tang, L. Shu et al., Scalable all-evaporation fabrication of efficient light-emitting diodes with hybrid 2D–3D perovskite nanostructures. Adv. Funct. Mater. **30**(39), 2002913 (2020). 10.1002/adfm.202002913

[139] Y. Hu, Q. Wang, Y.-L. Shi, M. Li, L. Zhang et al., Vacuum-evaporated all-inorganic cesium lead bromine perovskites for high-performance light-emitting diodes. J. Mater. Chem. C **5**(32), 8144–8149 (2017). 10.1039/c7tc02477k

[140] N. Kim, M. Shin, S. Jun, B. Choi, J. Kim et al., Highly efficient vacuum-evaporated CsPbBr_3_ perovskite light-emitting diodes with an electrical conductivity enhanced polymer-assisted passivation layer. ACS Appl. Mater. Interfaces **13**(31), 37323–37330 (2021). 10.1021/acsami.1c05447 34337932 10.1021/acsami.1c05447

[141] L. Song, L. Huang, Y. Liu, X. Guo, C. Geng et al., Efficient thermally evaporated perovskite light-emitting devices *via* a bilateral interface engineering strategy. J. Phys. Chem. Lett. **12**(26), 6165–6173 (2021). 10.1021/acs.jpclett.1c01592 34184904 10.1021/acs.jpclett.1c01592

[142] Y. Lian, Y. Wang, Y. Yuan, Z. Ren, W. Tang et al., Downscaling micro- and nano-perovskite LEDs. Nature **640**(8057), 62–68 (2025). 10.1038/s41586-025-08685-w 40108467 10.1038/s41586-025-08685-w

[143] E.G. Dyrvik, J.H. Warby, M.M. McCarthy, A.J. Ramadan, K.-A. Zaininger et al., Reducing nonradiative losses in perovskite LEDs through atomic layer deposition of Al_2_O_3_ on the hole-injection contact. ACS Nano **17**(4), 3289–3300 (2023). 10.1021/acsnano.2c04786 36790329 10.1021/acsnano.2c04786 PMC9979650

[144] Y. Shen, L.-P. Cheng, Y.-Q. Li, W. Li, J.-D. Chen et al., High-efficiency perovskite light-emitting diodes with synergetic outcoupling enhancement. Adv. Mater. **31**(24), e1901517 (2019). 10.1002/adma.201901517 31012195 10.1002/adma.201901517

[145] K. Wang, Y. Du, J. Liang, J. Zhao, F.F. Xu et al., Wettability-guided screen printing of perovskite microlaser arrays for current-driven displays. Adv. Mater. **32**(29), 2001999 (2020). 10.1002/adma.202001999 10.1002/adma.202001999 32510677

[146] D. Li, J. Wang, M. Li, G. Xie, B. Guo et al., Inkjet printing matrix perovskite quantum dot light-emitting devices. Adv. Mater. Technol. **5**(6), 2000099 (2020). 10.1002/admt.202000099

[147] C. Zou, C. Chang, D. Sun, K.F. Böhringer, L.Y. Lin, Photolithographic patterning of perovskite thin films for multicolor display applications. Nano Lett. **20**(5), 3710–3717 (2020). 10.1021/acs.nanolett.0c00701 32324409 10.1021/acs.nanolett.0c00701

[148] C. Zheng, X. Zheng, C. Feng, S. Ju, Z. Xu et al., High-brightness perovskite quantum dot light-emitting devices using inkjet printing. Org. Electron. **93**, 106168 (2021). 10.1016/j.orgel.2021.106168

[149] J. Wang, D. Li, L. Mu, M. Li, Y. Luo et al., Inkjet-printed full-color matrix quasi-two-dimensional perovskite light-emitting diodes. ACS Appl. Mater. Interfaces **13**(35), 41773–41781 (2021). 10.1021/acsami.1c07526 34432410 10.1021/acsami.1c07526

[150] J. Zhao, L.-W. Lo, H. Wan, P. Mao, Z. Yu et al., High-speed fabrication of all-inkjet-printed organometallic halide perovskite light-emitting diodes on elastic substrates. Adv. Mater. **33**(48), 2102095 (2021). 10.1002/adma.202102095 10.1002/adma.202102095 34623708

[151] Y. Li, Z. Chen, D. Liang, J. Zang, Z. Song et al., Coffee-stain-free perovskite film for efficient printed light-emitting diode. Adv. Opt. Mater. **9**(17), 2100553 (2021). 10.1002/adom.202100553

[152] S.-Y. Liang, Y.-F. Liu, H.-J. Zhang, Z.-K. Ji, H. Xia, High-quality patterning of CsPbBr_3_ perovskite films through lamination-assisted femtosecond laser ablation toward light-emitting diodes. ACS Appl. Mater. Interfaces **14**(41), 46958–46963 (2022). 10.1021/acsami.2c11870 36094822 10.1021/acsami.2c11870

[153] Z. Li, S. Chu, Y. Zhang, W. Chen, J. Chen et al., Mass transfer printing of metal-halide perovskite films and nanostructures. Adv. Mater. **34**(35), e2203529 (2022). 10.1002/adma.202203529 35908154 10.1002/adma.202203529

[154] D.H. Kim, H.J. An, J.-M. Myoung, Red-emitting micro PeLEDs for UHD displays by using capillary force lithography. Chem. Eng. J. **448**, 137727 (2022). 10.1016/j.cej.2022.137727

[155] W. Bai, T. Xuan, H. Zhao, S. Shi, X. Zhang et al., Microscale perovskite quantum dot light-emitting diodes (micro-PeLEDs) for full-color displays. Adv. Opt. Mater. **10**(12), 2200087 (2022). 10.1002/adom.202200087

[156] G. Vescio, J. Sanchez-Diaz, J.L. Frieiro, R.S. Sánchez, S. Hernández et al., 2D PEA_2_SnI_4_ inkjet-printed halide perovskite LEDs on rigid and flexible substrates. ACS Energy Lett. **7**(10), 3653–3655 (2022). 10.1021/acsenergylett.2c01773 36277130 10.1021/acsenergylett.2c01773 PMC9578039

[157] C. Wei, W. Su, J. Li, B. Xu, Q. Shan et al., A universal ternary-solvent-ink strategy toward efficient inkjet-printed perovskite quantum dot light-emitting diodes. Adv. Mater. **34**(10), e2107798 (2022). 10.1002/adma.202107798 34990514 10.1002/adma.202107798

[158] D. Li, J. Wang, M. Li, B. Guo, L. Mu et al., Efficient red perovskite quantum dot light-emitting diode fabricated by inkjet printing. Mater. Futures **1**(1), 015301 (2022). 10.1088/2752-5724/ac3568

[159] V.R.F. Schröder, N. Fratzscher, F. Mathies, E.R. Nandayapa, F. Hermerschmidt et al., Large area inkjet-printed metal halide perovskite LEDs enabled by gas flow assisted drying and crystallization. Nanoscale **15**(12), 5649–5654 (2023). 10.1039/D3NR00565H 36857678 10.1039/d3nr00565h

[160] M.-S. Kim, P. Sadhukhan, J.-M. Myoung, High-performance blue perovskite films and micro-arrays for light-emitting diodes with ionic liquid interlayer. Adv. Funct. Mater. **34**(1), 2309436 (2024). 10.1002/adfm.202309436

[161] G. Jang, D.-Y. Jo, S. Ma, J. Lee, J. Son et al., Core–shell perovskite quantum dots for highly selective room-temperature spin light-emitting diodes. Adv. Mater. **36**(5), 2309335 (2024). 10.1002/adma.202309335 10.1002/adma.202309335 37996975

[162] H. Liu, G. Shi, R. Khan, S. Chu, Z. Huang et al., Large-area flexible perovskite light-emitting diodes enabled by inkjet printing. Adv. Mater. **36**(8), 2309921 (2024). 10.1002/adma.202309921 10.1002/adma.202309921 38016083

[163] B. Ren, D. Zhang, X. Qiu, Y. Ding, Q. Zhang et al., Full-color fiber light-emitting diodes based on perovskite quantum wires. Sci. Adv. **10**(20), eadn1095 (2024). 10.1126/sciadv.adn1095 38748790 10.1126/sciadv.adn1095 PMC11095450

[164] V.R.F. Schröder, N. Fratzscher, N. Zorn Morales, D.S. Rühl, F. Hermerschmidt et al., Bicolour, large area, inkjet-printed metal halide perovskite light emitting diodes. Mater. Horiz. **11**(8), 1989–1996 (2024). 10.1039/d3mh02025h 38353605 10.1039/d3mh02025h

[165] Y. Huo, C. Luo, C. Wu, Z. Ren, H. Wang et al., Ambient direct lithography patterning of ultra-stable perovskite quantum dots for high-resolution light-emitting diodes. Adv. Funct. Mater. (2025). 10.1002/adfm.202504261

[166] L. Thi Ngo, Y.-T. Huang, C.-C. Chang, H. Verma, Y.-H. Lin et al., High-efficiency and ultrastable solvent-free curable perovskite quantum dot inks for microLED and LED backlighting applications. Nano Energy **142**, 111230 (2025). 10.1016/j.nanoen.2025.111230

[167] Q. Zhang, K. Yang, C. Luo, Z. Lin, W. Chen et al., Nanosecond response perovskite quantum dot light-emitting diodes with ultra-high resolution for active display application. Light. Sci. Appl. **14**, 285 (2025). 10.1038/s41377-025-01959-y 40841515 10.1038/s41377-025-01959-y PMC12370927

[168] C. Wang, J.M. Myoung, Spatially confined synthesis of CsPbBr 3 quantum dots for high-performance pure-blue light-emitting diodes. Matter (2025). 10.1016/j.matt.2025.102416

[169] J. Harwell, J. Burch, A. Fikouras, M.C. Gather, A. Di Falco et al., Patterning multicolor hybrid perovskite films *via* top-down lithography. ACS Nano **13**(4), 3823–3829 (2019). 10.1021/acsnano.8b09592 30794382 10.1021/acsnano.8b09592

[170] X. Zhou, Z. Gao, J. Shi, T. Li, S. Wei et al., Direct synthesis of perovskite quantum dot photoresist for direct photolithography. Angew. Chem. Int. Ed. **64**(1), e202413741 (2025). 10.1002/anie.202413741 10.1002/anie.202413741 39289158

[171] P. Zhang, G. Yang, F. Li, J. Shi, H. Zhong, Direct *in situ* photolithography of perovskite quantum dots based on photocatalysis of lead bromide complexes. Nat. Commun. **13**, 6713 (2022). 10.1038/s41467-022-34453-9 36344550 10.1038/s41467-022-34453-9 PMC9640639

[172] C.H. Lin, B. Cheng, T.Y. Li, J.R.D. Retamal, T.C. Wei et al., Orthogonal lithography for halide perovskite optoelectronic nanodevices. ACS Nano (2018). 10.1021/acsnano.8b05859 10.1021/acsnano.8b05859 30588789

[173] N. Lamers, Z. Zhang, J. Wallentin, Perovskite-compatible electron-beam-lithography process based on nonpolar solvents for single-nanowire devices. ACS Appl. Nano Mater. **5**(3), 3177–3182 (2022). 10.1021/acsanm.2c00188 35372798 10.1021/acsanm.2c00188 PMC8961732

[174] D. Lyashenko, A. Perez, A. Zakhidov, High-resolution patterning of organohalide lead perovskite pixels for photodetectors using orthogonal photolithography. Phys. Status Solidi A **214**(1), 1600302 (2017). 10.1002/pssa.201600302

[175] G.-H. Dun, H. Zhang, K. Qin, X. Tan, R. Zhao et al., Wafer-scale photolithography-pixeled Pb-free perovskite X-ray detectors. ACS Nano **16**(7), 10199–10208 (2022). 10.1021/acsnano.2c01074 35622531 10.1021/acsnano.2c01074

[176] B. Xia, M. Tu, B. Pradhan, F. Ceyssens, M.L. Tietze et al., Flexible metal halide perovskite photodetector arrays *via* photolithography and dry lift-off patterning. Adv. Eng. Mater. **24**, 2100930 (2022). 10.1002/adem.202100930

[177] S. Wei, J. Yuan, G. Yang, H. Zhong, Y. Dong et al., A photoinitiator-grafted photoresist for direct *in situ* lithography of perovskite quantum dots. ACS Appl. Nano Mater. **7**(23), 26397–26404 (2024). 10.1021/acsanm.3c06297

[178] F.H. Dill, W.P. Hornberger, P.S. Hauge, J.M. Shaw, Characterization of positive photoresist. IEEE Trans. Electron Devices **22**(7), 445–452 (1975). 10.1109/T-ED.1975.18159

[179] J.M. Shaw, J.D. Gelorme, N.C. LaBianca, W.E. Conley, S.J. Holmes, Negative photoresists for optical lithography. IBM J. Res. Dev. **41**(1.2), 81–94 (1997). 10.1147/rd.411.0081

[180] T. Haeger, R. Heiderhoff, T. Riedl, Thermal properties of metal-halide perovskites. J. Mater. Chem. C **8**(41), 14289–14311 (2020). 10.1039/d0tc03754k

[181] G. Niu, X. Guo, L. Wang, Review of recent progress in chemical stability of perovskite solar cells. J. Mater. Chem. A **3**(17), 8970–8980 (2015). 10.1039/C4TA04994B

[182] M. Lai, G. Parish, Y. Liu, J.M. Dell, A.J. Keating, Development of an alkaline-compatible porous-silicon photolithographic process. J. Microelectromech. Syst. **20**(2), 418–423 (2011). 10.1109/JMEMS.2011.2111356

[183] A.W. Flounders, D.L. Brandon, A.H. Bates, Patterning of immobilized antibody layers *via* photolithography and oxygen plasma exposure. Biosens. Bioelectron. **12**(6), 447–456 (1997). 10.1016/S0956-5663(96)00064-4 9253151 10.1016/s0956-5663(96)00064-4

[184] S.-Y. Lien, C.-W. Wang, W.-R. Chen, C.-H. Liu, C.-C. Kang et al., The influence of oxygen plasma on methylammonium lead iodide (MAPbI_3_) film doped with lead cesium triiodide (CsPbI_3_). Molecules **26**(17), 5133 (2021). 10.3390/molecules26175133 34500566 10.3390/molecules26175133 PMC8434561

[185] X. Kong, X. Fan, Y. Wang, Y. Luo, Y. Chen et al., Recent advances of photolithography patterning of quantum dots for micro-display applications. Nano Mater. Sci. **7**(1), 49–64 (2025). 10.1016/j.nanoms.2024.03.005

[186] X. Fan, S. Wang, X. Yang, C. Zhong, G. Chen et al., Brightened bicomponent perovskite nanocomposite based on Förster resonance energy transfer for micro-LED displays. Adv. Mater. **35**(30), 2300834 (2023). 10.1002/adma.202300834 10.1002/adma.202300834 37080636

[187] Y. Wang, Y. Luo, X. Kong, T. Wu, Y. Lin et al., Patterning technologies of quantum dots for color-conversion micro-LED display applications. Nanoscale **17**(4), 1764–1789 (2025). 10.1039/d4nr03925d 39688022 10.1039/d4nr03925d

[188] C. Cueto, D. Nikolla, A. Ribbe, J. Chambers, T. Emrick, Exploiting photohalide generation in shape and multichromatic color patterning of polymer–perovskite nanocomposites. J. Am. Chem. Soc. **147**(11), 9774–9785 (2025). 10.1021/jacs.4c18454 40063987 10.1021/jacs.4c18454

[189] W. Sun, F. Li, J. Tao, P. Li, L. Zhu et al., Micropore filling fabrication of high resolution patterned PQDs with a pixel size less than 5 μm. Nanoscale **14**(16), 5994–5998 (2022). 10.1039/d2nr01115h 35389395 10.1039/d2nr01115h

[190] T. Li, P. Zhang, S. Wei, Y. Jing, J. Shi et al., Polymerizable monomer solvents enabled direct *in situ* photolithography of perovskite quantum dots. Adv. Opt. Mater. **12**(20), 2400486 (2024). 10.1002/adom.202400486

[191] J. Chen, Y. Wu, X. Li, F. Cao, Y. Gu et al., Simple and fast patterning process by laser direct writing for perovskite quantum dots. Adv. Mater. Technol. **2**(10), 1700132 (2017). 10.1002/admt.201700132

[192] K. Sun, D. Tan, X. Fang, X. Xia, D. Lin et al., Three-dimensional direct lithography of stable perovskite nanocrystals in glass. Science **375**(6578), 307–310 (2022). 10.1126/science.abj2691 35050658 10.1126/science.abj2691

[193] X. Huang, Q. Guo, D. Yang, X. Xiao, X. Liu et al., Reversible 3D laser printing of perovskite quantum dots inside a transparent medium. Nat. Photonics **14**(2), 82–88 (2020). 10.1038/s41566-019-0538-8

[194] P. You, G. Li, G. Tang, J. Cao, F. Yan, Ultrafast laser-annealing of perovskite films for efficient perovskite solar cells. Energy Environ. Sci. **13**(4), 1187–1196 (2020). 10.1039/c9ee02324k

[195] L. Zhang, Y. Liu, Z. Gan, J. Su, Y. Gao, *In situ* localized formation of cesium lead bromide nanocomposites for fluorescence micro-patterning technology achieved by organic solvent polymerization. J. Mater. Chem. C **8**(10), 3409–3417 (2020). 10.1039/C9TC06687J

[196] W. Zhan, L. Meng, C. Shao, X.-G. Wu, K. Shi et al., *In situ* patterning perovskite quantum dots by direct laser writing fabrication. ACS Photonics **8**(3), 765–770 (2021). 10.1021/acsphotonics.1c00118

[197] A. Zhizhchenko, S. Syubaev, A. Berestennikov, A.V. Yulin, A. Porfirev et al., Single-mode lasing from imprinted halide-perovskite microdisks. ACS Nano **13**(4), 4140–4147 (2019). 10.1021/acsnano.8b08948 30844247 10.1021/acsnano.8b08948

[198] S.J. Kim, J. Byun, T. Jeon, H.M. Jin, H.R. Hong et al., Perovskite light-emitting diodes *via* laser crystallization: systematic investigation on grain size effects for device performance. ACS Appl. Mater. Interfaces **10**(3), 2490–2495 (2018). 10.1021/acsami.7b15470 29285922 10.1021/acsami.7b15470

[199] Z. Wan, Z. Liu, Q. Zhang, Q. Zhang, M. Gu, Laser technology for perovskite: fabrication and applications. Adv. Mater. Technol. **9**(10), 2302033 (2024). 10.1002/admt.202302033

[200] A.Y. Zhizhchenko, P. Tonkaev, D. Gets, A. Larin, D. Zuev et al., Light-emitting nanophotonic designs enabled by ultrafast laser processing of halide perovskites. Small **16**(19), 2000410 (2020). 10.1002/smll.202000410 10.1002/smll.202000410 32309903

[201] C. Vieu, F. Carcenac, A. Pépin, Y. Chen, M. Mejias et al., Electron beam lithography: resolution limits and applications. Appl. Surf. Sci. **164**(1–4), 111–117 (2000). 10.1016/S0169-4332(00)00352-4

[202] C. Zhu, H. Ekinci, A. Pan, B. Cui, X. Zhu, Electron beam lithography on nonplanar and irregular surfaces. Microsyst. Nanoeng. **10**, 52 (2024). 10.1038/s41378-024-00682-9 38646064 10.1038/s41378-024-00682-9 PMC11031580

[203] H.S. Kim, B.H. Son, Y.C. Kim, Y.H. Ahn, Direct laser writing lithography using a negative-tone electron-beam resist. Opt. Mater. Express **10**(11), 2813 (2020). 10.1364/ome.409302

[204] Y. Chen, Nanofabrication by electron beam lithography and its applications: a review. Microelectron. Eng. **135**, 57–72 (2015). 10.1016/j.mee.2015.02.042

[205] W.Y.E. Ong, Y.Z.D. Tan, L.J. Lim, T.G. Hoang, Z.-K. Tan, Crosslinkable ligands for high-density photo-patterning of perovskite nanocrystals. Adv. Mater. **37**(25), e2409564 (2025). 10.1002/adma.202409564 39374000 10.1002/adma.202409564

[206] Y. Fukuta, T. Miyata, Y. Hamanaka, Fabrication of two-dimensional hybrid organic–inorganic lead halide perovskites with controlled multilayer structures by liquid-phase laser ablation. J. Mater. Chem. C **11**(3), 910–916 (2023). 10.1039/D2TC04395E

[207] X. Huang, Q. Guo, S. Kang, T. Ouyang, Q. Chen et al., Three-dimensional laser-assisted patterning of blue-emissive metal halide perovskite nanocrystals inside a glass with switchable photoluminescence. ACS Nano **14**(3), 3150–3158 (2020). 10.1021/acsnano.9b08314 31994861 10.1021/acsnano.9b08314

[208] T.R. Groves, D. Pickard, B. Rafferty, N. Crosland, D. Adam et al., Maskless electron beam lithography: prospects, progress, and challenges. Microelectron. Eng. **61–62**, 285–293 (2002). 10.1016/S0167-9317(02)00528-2

[209] Z. Li, Z. Gao, L. Liu, K. Zhang, R. Ma et al., 3D patterning of perovskite quantum dots *via* direct *in situ* femtosecond laser writing. Nano Lett. **25**(18), 7410–7418 (2025). 10.1021/acs.nanolett.5c00861 40268341 10.1021/acs.nanolett.5c00861

[210] D. Wei, C. Wang, H. Wang, X. Hu, D. Wei et al., Experimental demonstration of a three-dimensional lithium niobate nonlinear photonic crystal. Nat. Photonics **12**(10), 596–600 (2018). 10.1038/s41566-018-0240-2

[211] S.-Y. Liang, Y.-F. Liu, S.-Y. Wang, Z.-K. Ji, H. Xia et al., High-resolution patterning of 2D perovskite films through femtosecond laser direct writing. Adv. Funct. Mater. **32**(38), 0224957 (2022). 10.1002/adfm.202204957

[212] Z. Wang, J. Zheng, G. Chen, K. Zhang, T. Wei et al., Laser-assisted thermal exposure lithography: arbitrary feature sizes. Adv. Eng. Mater. **23**(5), 2001468 (2021). 10.1002/adem.202001468

[213] D. Tan, Z. Wang, B. Xu, J. Qiu, Photonic circuits written by femtosecond laser in glass: improved fabrication and recent progress in photonic devices. Adv. Photon. **3**(2), 024002 (2021). 10.1117/1.ap.3.2.024002

[214] B. Jeong, H. Han, C. Park, Micro- and nanopatterning of halide perovskites where crystal engineering for emerging photoelectronics meets integrated device array technology. Adv. Mater. **32**(30), 2000597 (2020). 10.1002/adma.202000597 10.1002/adma.202000597 32530144

[215] L.J. Guo, Nanoimprint lithography: methods and material requirements. Adv. Mater. **19**(4), 495–513 (2007). 10.1002/adma.200600882

[216] S.Y. Chou, P.R. Krauss, P.J. Renstrom, Nanoimprint lithography. J. Vac. Sci. Technol. B Microelectron. Nanometer Struct. Process. Meas. Phenom. **14**(6), 4129–4133 (1996). 10.1116/1.588605

[217] Y. Yang, K. Mielczarek, M. Aryal, A. Zakhidov, W. Hu, Nanoimprinted polymer solar cell. ACS Nano **6**(4), 2877–2892 (2012). 10.1021/nn3001388 22394246 10.1021/nn3001388

[218] D.-Y. Khang, H. Kang, T.-I. Kim, H.H. Lee, Low-pressure nanoimprint lithography. Nano Lett. **4**(4), 633–637 (2004). 10.1021/nl049887d

[219] S.V. Makarov, V. Milichko, E.V. Ushakova, M. Omelyanovich, A. Cerdan Pasaran et al., Multifold emission enhancement in nanoimprinted hybrid perovskite metasurfaces. ACS Photonics **4**(4), 728–735 (2017). 10.1021/acsphotonics.6b00940

[220] H. Han, J.W. Oh, J. Park, H. Lee, C. Park et al., Hierarchically ordered perovskites with high photo-electronic and environmental stability *via* nanoimprinting guided block copolymer self-assembly. Adv. Mater. Interfaces **9**(16), 2200082 (2022). 10.1002/admi.202200082

[221] A. Cherala, P.N. Pandya, K.M. Liechti, S.V. Sreenivasan, Extending the resolution limits of nanoshape imprint lithography using molecular dynamics of polymer crosslinking. Microsyst. Nanoeng. **7**, 13 (2021). 10.1038/s41378-020-00225-y 34567728 10.1038/s41378-020-00225-y PMC8433368

[222] B. Jeong, H. Han, H.H. Kim, W.K. Choi, Y.J. Park et al., Polymer-assisted nanoimprinting for environment- and phase-stable perovskite nanopatterns. ACS Nano **14**(2), 1645–1655 (2020). 10.1021/acsnano.9b06980 31951365 10.1021/acsnano.9b06980

[223] K. Deng, Z. Liu, M. Wang, L. Li, Nanoimprinted grating-embedded perovskite solar cells with improved light management. Adv. Funct. Mater. **29**(19), 1900830 (2019). 10.1002/adfm.201900830

[224] M.G. Kang, L.J. Guo, Nanoimprinted semitransparent metal electrodes and their application in organic light-emitting diodes. Adv. Mater. **19**(10), 1391–1396 (2007). 10.1002/adma.200700134

[225] S. Wang, X. Dou, L. Chen, Y. Fang, A. Wang et al., Enhanced light out-coupling efficiency of quantum dot light emitting diodes by nanoimprint lithography. Nanoscale **10**(24), 11651–11656 (2018). 10.1039/C8NR02082E 29896589 10.1039/c8nr02082e

[226] R. Schmager, I.M. Hossain, F. Schackmar, B.S. Richards, G. Gomard et al., Light coupling to quasi-guided modes in nanoimprinted perovskite solar cells. Sol. Energy Mater. Sol. Cells **201**, 110080 (2019). 10.1016/j.solmat.2019.110080

[227] H. Wang, R. Haroldson, B. Balachandran, A. Zakhidov, S. Sohal et al., Nanoimprinted perovskite nanograting photodetector with improved efficiency. ACS Nano **10**(12), 10921–10928 (2016). 10.1021/acsnano.6b05535 28024335 10.1021/acsnano.6b05535

[228] S. Guo, Y.-S. Liu, X.-L. Zhang, Y.-F. Liu, Y.-G. Bi et al., Improved light extraction in all-inorganic perovskite light-emitting devices with periodic nanostructures by nanoimprinting lithography. Opt. Lett. **45**(18), 5156–5159 (2020). 10.1364/OL.404873 32932476 10.1364/OL.404873

[229] J. Mao, W.E.I. Sha, H. Zhang, X. Ren, J. Zhuang et al., Novel direct nanopatterning approach to fabricate periodically nanostructured perovskite for optoelectronic applications. Adv. Funct. Mater. **27**(10), 1606525 (2017). 10.1002/adfm.201606525

[230] N. Pourdavoud, S. Wang, A. Mayer, T. Hu, Y. Chen et al., Photonic nanostructures patterned by thermal nanoimprint directly into organo-metal halide perovskites. Adv. Mater. **29**(12), 1605003 (2017). 10.1002/adma.201605003 10.1002/adma.201605003 28102599

[231] T.-H. Kim, K.-S. Cho, E.K. Lee, S.J. Lee, J. Chae et al., Full-colour quantum dot displays fabricated by transfer printing. Nat. Photonics **5**(3), 176–182 (2011). 10.1038/nphoton.2011.12

[232] C. Wang, C. Linghu, S. Nie, C. Li, Q. Lei et al., Programmable and scalable transfer printing with high reliability and efficiency for flexible inorganic electronics. Sci. Adv. **6**(25), eabb2393 (2020). 10.1126/sciadv.abb2393 32596472 10.1126/sciadv.abb2393 PMC7299632

[233] T. Meng, Y. Zheng, D. Zhao, H. Hu, Y. Zhu et al., Ultrahigh-resolution quantum-dot light-emitting diodes. Nat. Photon. **16**(4), 297–303 (2022). 10.1038/s41566-022-00960-w

[234] T.W. Nam, M. Kim, Y. Wang, G.Y. Kim, W. Choi et al., Thermodynamic-driven polychromatic quantum dot patterning for light-emitting diodes beyond eye-limiting resolution. Nat. Commun. **11**(1), 3040 (2020). 10.1038/s41467-020-16865-7 32546822 10.1038/s41467-020-16865-7 PMC7297963

[235] C.K.W. Lee, Y. Pan, R. Yang, M. Kim, M.G. Li, Laser-induced transfer of functional materials. Top. Curr. Chem. **381**(4), 18 (2023). 10.1007/s41061-023-00429-6 10.1007/s41061-023-00429-6 PMC10200697 37212928

[236] M.A. Meitl, Z.-T. Zhu, V. Kumar, K.J. Lee, X. Feng et al., Transfer printing by kinetic control of adhesion to an elastomeric stamp. Nat. Mater. **5**(1), 33–38 (2006). 10.1038/nmat1532

[237] A. Carlson, A.M. Bowen, Y. Huang, R.G. Nuzzo, J.A. Rogers, Transfer printing techniques for materials assembly and micro/nanodevice fabrication. Adv. Mater. **24**(39), 5284–5318 (2012). 10.1002/adma.201201386 22936418 10.1002/adma.201201386

[238] J. Yoo, K. Lee, U.J. Yang, H.H. Song, J.H. Jang et al., Highly efficient printed quantum dot light-emitting diodes through ultrahigh-definition double-layer transfer printing. Nat. Photonics **18**(10), 1105–1112 (2024). 10.1038/s41566-024-01496-x

[239] J. Jang, Y.-G. Park, E. Cha, S. Ji, H. Hwang et al., 3D heterogeneous device arrays for multiplexed sensing platforms using transfer of perovskites. Adv. Mater. **33**(30), e2101093 (2021). 10.1002/adma.202101093 34142400 10.1002/adma.202101093

[240] Y. Yin, Z. Hu, M.U. Ali, M. Duan, L. Gao et al., Full-color micro-LED display with CsPbBr_3_ perovskite and CdSe quantum dots as color conversion layers. Adv. Mater. Technol. **5**(8), 2000251 (2020). 10.1002/admt.202000251

[241] Y. Li, F. Zhang, S. Wang, Regulatable interfacial adhesion between stamp and ink for transfer printing. Interdiscip. Mater. **3**(1), 29–53 (2024). 10.1002/idm2.12139

[242] J. McPhillimy, D. Jevtics, B.J.E. Guilhabert, C. Klitis, A. Hurtado et al., Automated nanoscale absolute accuracy alignment system for transfer printing. ACS Appl. Nano Mater. **3**(10), 10326–10332 (2020). 10.1021/acsanm.0c02224 33134883 10.1021/acsanm.0c02224 PMC7590505

[243] M. Kędziora, A. Opala, R. Mastria, L. De Marco, M. Król et al., Predesigned perovskite crystal waveguides for room-temperature exciton–polariton condensation and edge lasing. Nat. Mater. **23**(11), 1515–1522 (2024). 10.1038/s41563-024-01980-3 39160353 10.1038/s41563-024-01980-3

[244] G. Wang, D. Li, H.-C. Cheng, Y. Li, C.-Y. Chen et al., Wafer-scale growth of large arrays of perovskite microplate crystals for functional electronics and optoelectronics. Sci. Adv. **1**(9), e1500613 (2015). 10.1126/sciadv.1500613 26601297 10.1126/sciadv.1500613 PMC4646811

[245] Z. Xu, X. Han, W. Wu, F. Li, R. Wang et al., Controlled on-chip fabrication of large-scale perovskite single crystal arrays for high-performance laser and photodetector integration. Light Sci. Appl. **12**(1), 67 (2023). 10.1038/s41377-023-01107-4 36882401 10.1038/s41377-023-01107-4 PMC9992671

[246] M. Yuan, J. Feng, H. Li, H. Gao, Y. Qiu et al., Remote epitaxial crystalline perovskites for ultrahigh-resolution micro-LED displays. Nat. Nanotechnol. **20**(3), 381–387 (2025). 10.1038/s41565-024-01841-9 39815067 10.1038/s41565-024-01841-9

[247] M.K. Gangishetty, R.W.J. Scott, T.L. Kelly, Effect of relative humidity on crystal growth, device performance and hysteresis in planar heterojunction perovskite solar cells. Nanoscale **8**(12), 6300–6307 (2016). 10.1039/c5nr04179a 26411485 10.1039/c5nr04179a

[248] X. Duan, X. Li, L. Tan, Z. Huang, J. Yang et al., Controlling crystal growth *via* an autonomously longitudinal scaffold for planar perovskite solar cells. Adv. Mater. **32**(26), 2000617 (2020). 10.1002/adma.202000617 10.1002/adma.202000617 32449256

[249] T. Zhou, Z. Xu, R. Wang, X. Dong, Q. Fu et al., Crystal growth regulation of 2D/3D perovskite films for solar cells with both high efficiency and stability. Adv. Mater. **34**(17), 2200705 (2022). 10.1002/adma.202200705 10.1002/adma.202200705 35233866

[250] S. Wang, H. Luo, Z. Gu, R. Zhao, L. Guo et al., Crystal growth regulation of α-FAPbI3 perovskite films for high-efficiency solar cells with long-term stability. Adv. Funct. Mater. **33**(26), 2214834 (2023). 10.1002/adfm.202214834

[251] J.-W. Lee, D.-K. Lee, D.-N. Jeong, N.-G. Park, Control of crystal growth toward scalable fabrication of perovskite solar cells. Adv. Funct. Mater. **29**(47), 1807047 (2019). 10.1002/adfm.201807047

[252] H. Hu, M. Singh, X. Wan, J. Tang, C.-W. Chu et al., Nucleation and crystal growth control for scalable solution-processed organic–inorganic hybrid perovskite solar cells. J. Mater. Chem. A **8**(4), 1578–1603 (2020). 10.1039/c9ta11245f

[253] J. Yu, G. Liu, C. Chen, Y. Li, M. Xu et al., Perovskite CsPbBr_3_ crystals: growth and applications. J. Mater. Chem. C **8**(19), 6326–6341 (2020). 10.1039/d0tc00922a

[254] W. Chen, X. Li, Y. Li, Y. Li, A review: crystal growth for high-performance all-inorganic perovskite solar cells. Energy Environ. Sci. **13**(7), 1971–1996 (2020). 10.1039/d0ee00215a

[255] G. Hu, J. Guo, J. Jiang, L. Wang, J. Zhang et al., Capillary condensation-driven growth of perovskite nanowire arrays for multi-functional photodetector. Light Sci. Appl. **14**(1), 61 (2025). 10.1038/s41377-024-01680-2 39856055 10.1038/s41377-024-01680-2 PMC11761479

[256] R. Abe, A. Suzuki, K. Watanabe, A. Kikuchi, Fabrication of CH_3_NH_3_PbBr 3-based perovskite single-crystal arrays by spin-coating method using hydrophobic patterned substrate. Phys. Status Solidi A **217**(3), 1900511 (2020). 10.1002/pssa.201900511

[257] S. Jariwala, H. Sun, G.W.P. Adhyaksa, A. Lof, L.A. Muscarella et al., Local crystal misorientation influences non-radiative recombination in halide perovskites. Joule **3**(12), 3048–3060 (2019). 10.1016/j.joule.2019.09.001

[258] W. Chen, H. Chen, G. Xu, R. Xue, S. Wang et al., Precise control of crystal growth for highly efficient CsPbI_2_Br perovskite solar cells. Joule **3**(1), 191–204 (2019). 10.1016/j.joule.2018.10.011

[259] X. Zhou, Y. Cai, M. Xu, J. Li, C. Sheng et al., Dewetting-assisted patterning of organic semiconductors for micro-OLED arrays with a pixel size of 1 µm. Small Methods **6**(4), 2101509 (2022). 10.1002/smtd.202101509 10.1002/smtd.202101509 35170861

[260] J. Hu, Z. Li, P. Huang, L. Huang, S. Xu, *In situ* dewetting assisted plasma etching of large-scale uniform nanocones on arbitrarily structured glass elements. Adv. Funct. Mater. **34**(51), 2410563 (2024). 10.1002/adfm.202410563

[261] J. Zhang, Y. Yang, W. Li, Z. Tang, Z. Hu et al., Precise arraying of perovskite single crystals through droplet-assisted self-alignment. Sci. Adv. **10**(28), eado0873 (2024). 10.1126/sciadv.ado0873 38985869 10.1126/sciadv.ado0873 PMC11235166

[262] L. Shi, L. Meng, F. Jiang, Y. Ge, F. Li et al., *In situ* inkjet printing strategy for fabricating perovskite quantum dot patterns. Adv. Funct. Mater. **29**(37), 1903648 (2019). 10.1002/adfm.201903648

[263] H. Eggers, F. Schackmar, T. Abzieher, Q. Sun, U. Lemmer et al., Inkjet-printed micrometer-thick perovskite solar cells with large columnar grains. Adv. Energy Mater. **10**(6), 1903184 (2020). 10.1002/aenm.201903184

[264] C. Liang, P. Li, H. Gu, Y. Zhang, F. Li et al., One-step inkjet printed perovskite in air for efficient light harvesting. Sol. RRL **2**(2), 1700217 (2018). 10.1002/solr.201700217

[265] J. Zhao, L.-W. Lo, Z. Yu, C. Wang, Handwriting of perovskite optoelectronic devices on diverse substrates. Nat. Photon. **17**(11), 964–971 (2023). 10.1038/s41566-023-01266-1

[266] M. Duan, Z. Feng, Y. Wu, Y. Yin, Z. Hu et al., Inkjet-printed micrometer-thick patterned perovskite quantum dot films for efficient blue-to-green photoconversion. Adv. Mater. Technol. **4**(12), 1900779 (2019). 10.1002/admt.201900779

[267] S. Wang, X. Kong, S. Cai, Y. Luo, Y. Gu et al., Solvent engineering in perovskite nanocrystal colloid inks for super-fine electrohydrodynamic inkjet printing of color conversion microstructures in micro-LED displays. Chin. Chem. Lett. **36**(8), 110976 (2025). 10.1016/j.cclet.2025.110976

[268] F. Schackmar, H. Eggers, M. Frericks, B.S. Richards, U. Lemmer et al., Perovskite solar cells with all-inkjet-printed absorber and charge transport layers. Adv. Mater. Technol. **6**(2), 2000271 (2021). 10.1002/admt.202000271

[269] B. Derby, Inkjet printing of functional and structural materials: fluid property requirements, feature stability, and resolution. Annu. Rev. Mater. Res. **40**, 395–414 (2010). 10.1146/annurev-matsci-070909-104502

[270] X. Peng, J. Yuan, S. Shen, M. Gao, A.S.R. Chesman et al., Perovskite and organic solar cells fabricated by inkjet printing: progress and prospects. Adv. Funct. Mater. **27**(41), 1703704 (2017). 10.1002/adfm.201703704

[271] D. Lohse, Fundamental fluid dynamics challenges in inkjet printing. Annu. Rev. Fluid Mech. **54**, 349–382 (2022). 10.1146/annurev-fluid-022321-114001

[272] N. Reis, C. Ainsley, B. Derby, Ink-jet delivery of particle suspensions by piezoelectric droplet ejectors. J. Appl. Phys. **97**(9), 094903 (2005). 10.1063/1.1888026

[273] O.A. Basaran, H. Gao, P.P. Bhat, Nonstandard inkjets. Annu. Rev. Fluid Mech. **45**, 85–113 (2013). 10.1146/annurev-fluid-120710-101148

[274] L. Zhang, S. Chen, J. Zeng, Z. Jiang, Q. Ai et al., Inkjet-printing controlled phase evolution boosts the efficiency of hole transport material free and carbon-based CsPbBr_3_ perovskite solar cells exceeding 9%. Energy Environ. Mater. **7**(2), e12543 (2024). 10.1002/eem2.12543

[275] F. Mathies, E.J.W. List-Kratochvil, E.L. Unger, Advances in inkjet-printed metal halide perovskite photovoltaic and optoelectronic devices. Energy Technol. **8**(4), 1900991 (2020). 10.1002/ente.201900991

[276] J.E. Fromm, Numerical calculation of the fluid dynamics of drop-on-demand jets. IBM J. Res. Dev. **28**(3), 322–333 (1984). 10.1147/rd.283.0322

[277] N. Reis, B. Derby, Ink jet deposition of ceramic suspensions: modeling and experiments of droplet formation. MRS Online Proc. Libr. **625**(1), 117 (2000). 10.1557/PROC-625-117

[278] J. Eggers, Universal pinching of 3D axisymmetric free-surface flow. Phys. Rev. Lett. **71**(21), 3458–3460 (1993). 10.1103/PhysRevLett.71.3458 10054982 10.1103/PhysRevLett.71.3458

[279] J. Eggers, Nonlinear dynamics and breakup of free-surface flows. Rev. Mod. Phys. **69**(3), 865–930 (1997). 10.1103/revmodphys.69.865

[280] T.A. Cohen, D. Sharp, K.T. Kluherz, Y. Chen, C. Munley et al., Direct patterning of perovskite nanocrystals on nanophotonic cavities with electrohydrodynamic inkjet printing. Nano Lett. **22**(14), 5681–5688 (2022). 10.1021/acs.nanolett.2c00473 35819950 10.1021/acs.nanolett.2c00473

[281] Y. Chen, X. Yang, X. Fan, A. Kang, X. Kong et al., Electrohydrodynamic inkjet printing of three-dimensional perovskite nanocrystal arrays for full-color micro-LED displays. ACS Appl. Mater. Interfaces **16**(19), 24908–24919 (2024). 10.1021/acsami.4c02594 38706177 10.1021/acsami.4c02594

[282] X. Yang, S. Wang, Y. Hou, Y. Wang, T. Zhang et al., Dual-ligand red perovskite ink for electrohydrodynamic printing color conversion arrays over 2540 dpi in near-eye micro-LED display. Nano Lett. **24**(12), 3661–3669 (2024). 10.1021/acs.nanolett.3c04927 38408021 10.1021/acs.nanolett.3c04927

[283] F. Hermerschmidt, F. Mathies, V.R.F. Schröder, C. Rehermann, N.Z. Morales et al., Finally, inkjet-printed metal halide perovskite LEDs–utilizing seed crystal templating of salty PEDOT: PSS. Mater. Horiz. **7**(7), 1773–1781 (2020). 10.1039/d0mh00512f

[284] J. Philip, P.D. Shima, B. Raj, Enhancement of thermal conductivity in magnetite based nanofluid due to chainlike structures. Appl. Phys. Lett. **91**(20), 203108 (2007). 10.1063/1.2812699

[285] Z. Li, P. Li, G. Chen, Y. Cheng, X. Pi et al., Ink engineering of inkjet printing perovskite. ACS Appl. Mater. Interfaces **12**(35), 39082–39091 (2020). 10.1021/acsami.0c09485 32805912 10.1021/acsami.0c09485

[286] Y. Cheng, H. Wu, J. Ma, P. Li, Z. Gu et al., Droplet manipulation and crystallization regulation in inkjet-printed perovskite film formation. CCS Chem. **4**(5), 1465–1485 (2022). 10.31635/ccschem.022.202101583

[287] Z. Zhang, Z. Li, Y. Chen, Z. Zhang, K. Fan et al., Progress on inkjet printing technique for perovskite films and their optoelectronic and optical applications. ACS Photonics **10**(10), 3435–3450 (2023). 10.1021/acsphotonics.3c00897

[288] Y.-J. Choi, K. Eun, R. Bail, C. Doo, Systematic development of a novel ternary solvent system for uniform inkjet printing of organic light-emitting diodes. ACS Appl. Mater. Interfaces **17**(17), 25546–25561 (2025). 10.1021/acsami.5c00941 40234244 10.1021/acsami.5c00941

[289] J. Stringer, B. Derby, Limits to feature size and resolution in ink jet printing. J. Eur. Ceram. Soc. **29**(5), 913–918 (2009). 10.1016/j.jeurceramsoc.2008.07.016

[290] F.F. Jackson, K.J. Kubiak, M.C.T. Wilson, M. Molinari, V. Stetsyuk, Droplet misalignment limit for inkjet printing into cavities on textured surfaces. Langmuir **35**(29), 9564–9571 (2019). 10.1021/acs.langmuir.9b00649 31287703 10.1021/acs.langmuir.9b00649

[291] M. Rump, U. Sen, R. Jeurissen, H. Reinten, M. Versluis et al., Selective evaporation at the nozzle exit in piezoacoustic inkjet printing. Phys. Rev. Appl. **19**(5), 054056 (2023). 10.1103/physrevapplied.19.054056

[292] J.-U. Park, M. Hardy, S.J. Kang, K. Barton, K. Adair et al., High-resolution electrohydrodynamic jet printing. Nat. Mater. **6**(10), 782–789 (2007). 10.1038/nmat1974 17676047 10.1038/nmat1974

[293] Y. Jang, J. Kim, D. Byun, Invisible metal-grid transparent electrode prepared by electrohydrodynamic (EHD) jet printing. J. Phys. D Appl. Phys. **46**(15), 155103 (2013). 10.1088/0022-3727/46/15/155103

[294] C. Wei, H. Qin, N.A. Ramírez-Iglesias, C.-P. Chiu, Y.-S. Lee et al., High-resolution ac-pulse modulated electrohydrodynamic jet printing on highly insulating substrates. J. Micromech. Microeng. **24**(4), 045010 (2014). 10.1088/0960-1317/24/4/045010

[295] Y. Duan, R. Yu, H. Zhang, W. Yang, W. Xie et al., Programmable, high-resolution printing of spatially graded perovskites for multispectral photodetectors. Adv. Mater. **36**(24), 2313946 (2024). 10.1002/adma.202313946 10.1002/adma.202313946 38582876

[296] Q. Wang, G. Zhang, H. Zhang, Y. Duan, Z. Yin et al., High-resolution, flexible, and full-color perovskite image photodetector *via* electrohydrodynamic printing of ionic-liquid-based ink. Adv. Funct. Mater. **31**(28), 2100857 (2021). 10.1002/adfm.202100857

[297] X. Yang, Z.-J. Yan, C.-M. Zhong, H. Jia, G.-L. Chen et al., Electrohydrodynamically printed high-resolution arrays based on stabilized CsPbBr_3_ quantum dot inks. Adv. Opt. Mater. **11**(9), 2202673 (2023). 10.1002/adom.202202673

[298] S. Wang, D. Chen, K. Xu, J. Hu, S. Liang et al., Boosting stability and inkjet printability of pure-red CsPb(Br/I)_3_ quantum dots through dual-shell encapsulation for micro-LED displays. ACS Energy Lett. **9**(6), 2517–2526 (2024). 10.1021/acsenergylett.4c00803

[299] M.H. Saba, S. Mukherjee, S. Dutta, P. Kumar Mallisetty, N. Chandra Murmu, Electrohydrodynamic jet printing for desired print diameter. Mater. Today Proc. **46**, 1749–1754 (2021). 10.1016/j.matpr.2020.07.570

[300] Y. Guan, M. Wang, S. Wu, Y. Sha, Y. Tian et al., The internal flow behaviors during Taylor cone formation of pulsating electrohydrodynamic jet printing. Phys. Fluids **34**(12), 122007 (2022). 10.1063/5.0124688

[301] B.W. Wagoner, P.M. Vlahovska, M.T. Harris, O.A. Basaran, Electrohydrodynamics of lenticular drops and equatorial streaming. J. Fluid Mech. **925**, A36 (2021). 10.1017/jfm.2021.651

[302] S.N. Reznik, E. Zussman, Capillary-dominated electrified jets of a viscous leaky dielectric liquid. Phys. Rev. E **81**(2), 026313 (2010). 10.1103/physreve.81.026313 10.1103/PhysRevE.81.026313 20365657

[303] J. Beroz, A.J. Hart, J.W.M. Bush, Stability limit of electrified droplets. Phys. Rev. Lett. **122**(24), 244501 (2019). 10.1103/physrevlett.122.244501 31322400 10.1103/PhysRevLett.122.244501

[304] M. Firouznia, M.J. Miksis, P.M. Vlahovska, D. Saintillan, Instability of a planar fluid interface under a tangential electric field in a stagnation point flow. J. Fluid Mech. **931**, A25 (2022). 10.1017/jfm.2021.967

[305] Y. Liu, F. Li, L. Qiu, K. Yang, Q. Li et al., Fluorescent microarrays of *in situ* crystallized perovskite nanocomposites fabricated for patterned applications by using inkjet printing. ACS Nano **13**(2), 2042–2049 (2019). 10.1021/acsnano.8b08582 30735353 10.1021/acsnano.8b08582

[306] R. Yu, W. Xie, W. Yang, X. Yang, Y. Duan, Precise *in-situ* fabrication of perovskite single crystal arrays *via* cosolvent based electrohydrodynamic printing. J. Micromech. Microeng. **34**(2), 025008 (2024). 10.1088/1361-6439/ad1b1b

[307] G. Zhang, H. Zhang, R. Yu, Y. Duan, Y. Huang et al., Critical size/viscosity for coffee-ring-free printing of perovskite micro/nanopatterns. ACS Appl. Mater. Interfaces **14**(12), 14712–14720 (2022). 10.1021/acsami.1c23630 35297596 10.1021/acsami.1c23630

[308] Z. Yin, D. Wang, Y. Guo, Z. Zhao, L. Li et al., Electrohydrodynamic printing for high resolution patterning of flexible electronics toward industrial applications. InfoMat **6**(2), e12505 (2024). 10.1002/inf2.12505

[309] A. Speidel, I. Bisterov, K.K. Saxena, M. Zubayr, D. Reynaerts et al., Electrochemical jet manufacturing technology: from fundamentals to application. Int. J. Mach. Tools Manuf **180**, 103931 (2022). 10.1016/j.ijmachtools.2022.103931

[310] N. Mkhize, H. Bhaskaran, Electrohydrodynamic jet printing: introductory concepts and considerations. Small Science **2**(2), 2100073 (2022). 10.1002/smsc.202100073 40213534 10.1002/smsc.202100073 PMC11935840

[311] Y. Li, G. Zhang, J. Zhang, D. Song, C. Guo et al., Advanced multi-nozzle electrohydrodynamic printing: mechanism, processing, and diverse applications at micro/nano-scale. Int. J. Extreme Manuf. **7**(1), 012008 (2025). 10.1088/2631-7990/ad8d22

[312] W. Yang, Y. Duan, J. Gao, Z. Yin, Crosstalk elimination for large-scale, high-density electrohydrodynamic printing *via* optimization of nozzle material and structure. Addit. Manuf. **77**, 103815 (2023). 10.1016/j.addma.2023.103815

[313] L. Peng, Y. Pan, Z. Wang, Y. Feng, Z. Liu, Design and evaluation of a linear nozzle array with double auxiliary electrodes for restraining cross-talk effect in parallel electrohydrodynamic jet printing. J. Micromech. Microeng. **32**(10), 105009 (2022). 10.1088/1361-6439/ac8f53

[314] M. Wojasiński, J. Goławski, T. Ciach, Blow-assisted multi-jet electrospinning of poly-L-lactic acid nanofibers. J. Polym. Res. **24**(5), 76 (2017). 10.1007/s10965-017-1233-4

[315] J.D. Regele, M.J. Papac, M.J.A. Rickard, D. Dunn-Rankin, Effects of capillary spacing on EHD spraying from an array of cone jets. J. Aerosol Sci. **33**(11), 1471–1479 (2002). 10.1016/S0021-8502(02)00093-9

[316] A. Khan, K. Rahman, S. Ali, S. Khan, B. Wang et al., Fabrication of circuits by multi-nozzle electrohydrodynamic inkjet printing for soft wearable electronics. J. Mater. Res. **36**(18), 3568–3578 (2021). 10.1557/s43578-021-00188-4

[317] M. Chen, H. Lee, J. Yang, Z. Xu, N. Huang et al., Parallel, multi-material electrohydrodynamic 3D nanoprinting. Small **16**(13), e1906402 (2020). 10.1002/smll.201906402 32101385 10.1002/smll.201906402

[318] M. Chen, J. Yang, Z. Wang, Z. Xu, H. Lee et al., 3D nanoprinting of perovskites. Adv. Mater. **31**(44), 1904073 (2019). 10.1002/adma.201904073 10.1002/adma.201904073 31544295

[319] M. Chen, S. Hu, Z. Zhou, N. Huang, S. Lee et al., Three-dimensional perovskite nanopixels for ultrahigh-resolution color displays and multilevel anticounterfeiting. Nano Lett. **21**(12), 5186–5194 (2021). 10.1021/acs.nanolett.1c01261 34125558 10.1021/acs.nanolett.1c01261

[320] M. Chen, Z. Zhou, S. Hu, N. Huang, H. Lee et al., 3D printing of arbitrary perovskite nanowire heterostructures. Adv. Funct. Mater. **33**(15), 2212146 (2023). 10.1002/adfm.202212146

[321] R. Patidar, D. Burkitt, K. Hooper, D. Richards, T. Watson, Slot-die coating of perovskite solar cells: an overview. Mater. Today Commun. **22**, 100808 (2020). 10.1016/j.mtcomm.2019.100808

[322] Z. Yang, W. Zhang, S. Wu, H. Zhu, Z. Liu et al., Slot-die coating large-area formamidinium-cesium perovskite film for efficient and stable parallel solar module. Sci. Adv. **7**(18), eabg3749 (2021). 10.1126/sciadv.abg3749 33931458 10.1126/sciadv.abg3749 PMC8087413

[323] L. Tan, J. Zhou, X. Zhao, S. Wang, M. Li et al., Combined vacuum evaporation and solution process for high-efficiency large-area perovskite solar cells with exceptional reproducibility. Adv. Mater. **35**(13), 2205027 (2023). 10.1002/adma.202205027 10.1002/adma.202205027 36681866

[324] Y. Gong, Z. Gong, Laser-based micro/nano-processing techniques for microscale LEDs and full-color displays. Adv. Mater. Technol. **8**(5), 2200949 (2023). 10.1002/admt.202200949

[325] S.-J. Woo, J.S. Kim, T.-W. Lee, Characterization of stability and challenges to improve lifetime in perovskite LEDs. Nat. Photonics **15**(9), 630–634 (2021). 10.1038/s41566-021-00863-2

[326] H. Tsai, W. Nie, J.-C. Blancon, C.C. Stoumpos, C.M.M. Soe et al., Stable light-emitting diodes using phase-pure ruddlesden-popper layered perovskites. Adv. Mater. **30**(6), 1704217 (2018). 10.1002/adma.201704217 10.1002/adma.201704217 29314326

[327] H. Kim, J.S. Kim, J.-M. Heo, M. Pei, I.-H. Park et al., Proton-transfer-induced 3D/2D hybrid perovskites suppress ion migration and reduce luminance overshoot. Nat. Commun. **11**(1), 3378 (2020). 10.1038/s41467-020-17072-0 32632144 10.1038/s41467-020-17072-0 PMC7338442

[328] S. Bai, P. Da, C. Li, Z. Wang, Z. Yuan et al., Planar perovskite solar cells with long-term stability using ionic liquid additives. Nature **571**(7764), 245–250 (2019). 10.1038/s41586-019-1357-2 31292555 10.1038/s41586-019-1357-2

[329] Y. Zou, L. Cai, T. Song, B. Sun, Recent progress on patterning strategies for perovskite light-emitting diodes toward a full-color display prototype. Small Sci. **1**(8), 2000050 (2021). 10.1002/smsc.202000050 40213164 10.1002/smsc.202000050 PMC11935914

[330] W. Fu, A.G. Ricciardulli, Q.A. Akkerman, R.A. John, M.M. Tavakoli et al., Stability of perovskite materials and devices. Mater. Today **58**, 275–296 (2022). 10.1016/j.mattod.2022.06.020

[331] B. Guo, R. Lai, S. Jiang, L. Zhou, Z. Ren et al., Ultrastable near-infrared perovskite light-emitting diodes. Nat. Photon. **16**(9), 637–643 (2022). 10.1038/s41566-022-01046-3

[332] X. Zhao, L.J. Lim, S.S. Ang, Z.-K. Tan, Efficient short-wave infrared light-emitting diodes based on heavy-metal-free quantum dots. Adv. Mater. **34**(45), 2206409 (2022). 10.1002/adma.202206409 10.1002/adma.202206409 36097727

[333] M. Wang, W. Wang, B. Ma, W. Shen, L. Liu et al., Lead-free perovskite materials for solar cells. Nano-Micro Lett. **13**(1), 62 (2021). 10.1007/s40820-020-00578-z 10.1007/s40820-020-00578-z PMC8187519 34138241

[334] X. Li, X. Gao, X. Zhang, X. Shen, M. Lu et al., Lead-free halide perovskites for light emission: recent advances and perspectives. Adv. Sci. **8**(4), 2003334 (2021). 10.1002/advs.202003334 10.1002/advs.202003334 PMC7887601 33643803

[335] H. Tang, Y. Xu, X. Hu, Q. Hu, T. Chen et al., Lead-free halide double perovskite nanocrystals for light-emitting applications: strategies for boosting efficiency and stability. Adv. Sci. **8**(7), 2004118 (2021). 10.1002/advs.202004118 10.1002/advs.202004118 PMC8025037 33854898

[336] W.-L. Hong, Y.-C. Huang, C.-Y. Chang, Z.-C. Zhang, H.-R. Tsai et al., Efficient low-temperature solution-processed lead-free perovskite infrared light-emitting diodes. Adv. Mater. **28**(36), 8029–8036 (2016). 10.1002/adma.201601024 27376676 10.1002/adma.201601024

[337] H. Min, N. Wang, N. Chen, Y. Tong, Y. Wang et al., Spin coating epitaxial heterodimensional tin perovskites for light-emitting diodes. Nat. Nanotechnol. **19**(5), 632–637 (2024). 10.1038/s41565-023-01588-9 38216685 10.1038/s41565-023-01588-9

[338] H. Liang, F. Yuan, A. Johnston, C. Gao, H. Choubisa et al., High color purity lead-free perovskite light-emitting diodes *via* Sn stabilization. Adv. Sci. **7**(8), 1903213 (2020). 10.1002/advs.201903213 10.1002/advs.201903213 PMC7175260 32328423

[339] T.C. Jellicoe, J.M. Richter, H.F.J. Glass, M. Tabachnyk, R. Brady et al., Synthesis and optical properties of lead-free cesium tin halide perovskite nanocrystals. J. Am. Chem. Soc. **138**(9), 2941–2944 (2016). 10.1021/jacs.5b13470 26901659 10.1021/jacs.5b13470

[340] S. Lin, Z. Ma, X. Ji, Q. Zhou, W. Chu et al., Efficient large-area (81 cm^2^) ternary copper halides light-emitting diodes with external quantum efficiency exceeding 13% *via* host-guest strategy. Adv. Mater. **36**(29), 2313570 (2024). 10.1002/adma.202313570 10.1002/adma.202313570 38693828

[341] S. Lin, Z. Ma, X. Ji, W. Chu, Q. Zhou et al., Efficient non-doped cluster light-emitting diodes based on semiconducting copper iodide hybrids. J. Lumin. **271**, 120609 (2024). 10.1016/j.jlumin.2024.120609

[342] Z. Ma, X. Ji, S. Lin, X. Chen, D. Wu et al., Recent advances and opportunities of eco-friendly ternary copper halides: a new superstar in optoelectronic applications. Adv. Mater. **35**(44), 2300731 (2023). 10.1002/adma.202300731 10.1002/adma.202300731 36854310

[343] M. Leng, Y. Yang, K. Zeng, Z. Chen, Z. Tan et al., All-inorganic bismuth-based perovskite quantum dots with bright blue photoluminescence and excellent stability. Adv. Funct. Mater. **28**(1), 1704446 (2018). 10.1002/adfm.201704446

[344] N.K. Noel, S.D. Stranks, A. Abate, C. Wehrenfennig, S. Guarnera et al., Lead-free organic–inorganic tin halide perovskites for photovoltaic applications. Energy Environ. Sci. **7**(9), 3061–3068 (2014). 10.1039/c4ee01076k

[345] X. Xiao, M. Wang, S. Chen, Y. Zhang, H. Gu et al., Lead-adsorbing ionogel-based encapsulation for impact-resistant, stable, and lead-safe perovskite modules. Sci. Adv. **7**(44), eabi8249 (2021). 10.1126/sciadv.abi8249 34714678 10.1126/sciadv.abi8249 PMC8555895

[346] B. Chen, C. Fei, S. Chen, H. Gu, X. Xiao et al., Recycling lead and transparent conductors from perovskite solar modules. Nat. Commun. **12**, 5859 (2021). 10.1038/s41467-021-26121-1 34615875 10.1038/s41467-021-26121-1 PMC8494795

[347] X. Feng, Q. Guo, J. Xiu, Z. Ying, K.W. Ng et al., Close-loop recycling of perovskite solar cells through dissolution-recrystallization of perovskite by butylamine. Cell Rep. Phys. Sci. **2**(2), 100341 (2021). 10.1016/j.xcrp.2021.100341

[348] M. Zhang, X. Ma, J.L. Esguerra, H. Yu, O. Hjelm et al., Towards sustainable perovskite light-emitting diodes. Nat. Sustain. **8**(3), 315–324 (2025). 10.1038/s41893-024-01503-7

